# Emergence of Novel RNA-Editing Sites by Changes in the Binding Affinity of a Conserved PPR Protein

**DOI:** 10.1093/molbev/msac222

**Published:** 2022-10-13

**Authors:** F Vanessa Loiacono, Dirk Walther, Stefanie Seeger, Wolfram Thiele, Ines Gerlach, Daniel Karcher, Mark Aurel Schöttler, Reimo Zoschke, Ralph Bock

**Affiliations:** Department of Organelle Biology, Biotechnology and Molecular Ecophysiology, Max-Planck-Institut für Molekulare Pflanzenphysiologie, Am Mühlenberg 1, D-14476 Potsdam-Golm, Germany; Department of Organelle Biology, Biotechnology and Molecular Ecophysiology, Max-Planck-Institut für Molekulare Pflanzenphysiologie, Am Mühlenberg 1, D-14476 Potsdam-Golm, Germany; Department of Organelle Biology, Biotechnology and Molecular Ecophysiology, Max-Planck-Institut für Molekulare Pflanzenphysiologie, Am Mühlenberg 1, D-14476 Potsdam-Golm, Germany; Department of Organelle Biology, Biotechnology and Molecular Ecophysiology, Max-Planck-Institut für Molekulare Pflanzenphysiologie, Am Mühlenberg 1, D-14476 Potsdam-Golm, Germany; Department of Organelle Biology, Biotechnology and Molecular Ecophysiology, Max-Planck-Institut für Molekulare Pflanzenphysiologie, Am Mühlenberg 1, D-14476 Potsdam-Golm, Germany; Department of Organelle Biology, Biotechnology and Molecular Ecophysiology, Max-Planck-Institut für Molekulare Pflanzenphysiologie, Am Mühlenberg 1, D-14476 Potsdam-Golm, Germany; Department of Organelle Biology, Biotechnology and Molecular Ecophysiology, Max-Planck-Institut für Molekulare Pflanzenphysiologie, Am Mühlenberg 1, D-14476 Potsdam-Golm, Germany; Department of Organelle Biology, Biotechnology and Molecular Ecophysiology, Max-Planck-Institut für Molekulare Pflanzenphysiologie, Am Mühlenberg 1, D-14476 Potsdam-Golm, Germany; Department of Organelle Biology, Biotechnology and Molecular Ecophysiology, Max-Planck-Institut für Molekulare Pflanzenphysiologie, Am Mühlenberg 1, D-14476 Potsdam-Golm, Germany

**Keywords:** RNA editing, chloroplast, PPR protein, co-evolution, RNA-binding protein, PPR code

## Abstract

RNA editing converts cytidines to uridines in plant organellar transcripts. Editing typically restores codons for conserved amino acids. During evolution, specific C-to-U editing sites can be lost from some plant lineages by genomic C-to-T mutations. By contrast, the emergence of novel editing sites is less well documented. Editing sites are recognized by pentatricopeptide repeat (PPR) proteins with high specificity. RNA recognition by PPR proteins is partially predictable, but prediction is often inadequate for PPRs involved in RNA editing. Here we have characterized evolution and recognition of a recently gained editing site. We demonstrate that changes in the RNA recognition motifs that are not explainable with the current PPR code allow an ancient PPR protein, QED1, to uniquely target the *ndhB*-291 site in Brassicaceae. When expressed in tobacco, the *Arabidopsis* QED1 edits 33 high-confident off-target sites in chloroplasts and mitochondria causing a spectrum of mutant phenotypes. By manipulating the relative expression levels of QED1 and *ndhB*-291, we show that the target specificity of the PPR protein depends on the RNA:protein ratio. Finally, our data suggest that the low expression levels of PPR proteins are necessary to ensure the specificity of editing site selection and prevent deleterious off-target editing.

## Introduction

In the DNA-containing organelles (mitochondria and chloroplasts) of land plants, RNA editing by cytidine-to-uridine (C-to-U) transitions occurs at specific transcript positions. With a few exceptions, almost all embryophytes possess C-to-U editing in both chloroplasts and mitochondria (reviewed in (Small et al. 2020)). Most of the edited Cs occur at first or second codon positions and alter the information encoded by the edited triplet. In this way, RNA editing in plant organelles usually restores codons that are evolutionally conserved and essential for protein function (Maier et al. 1996).

Because of the strong mutation bias towards C-to-T transitions in DNA, editing sites were frequently lost during the evolution of flowering plants by reversions to genomic Ts (Sloan et al. 2010; Fujii and Small 2011; Loiacono et al. 2019). Up to several thousand sites are edited in the organelles of hornworts, ferns, and lycopods (Freyer et al. 1997; Kugita et al. 2003; Wolf et al. 2004; Tillich et al. 2006; Smith 2009; Oldenkott et al. 2014). By contrast, a typical angiosperm species edits only 30–40 sites in the chloroplasts and around 500 in the mitochondria. Early-branching angiosperms such as *Amborella trichopoda*, possess a higher number of sites in their organelles than modern flowering plants (Hein et al. 2016). The latter are believed to have lost most of the ancient sites, without gaining editing at new positions (reviewed in Fujii and Small (2011)).

The cytidines that undergo editing are recognized by *trans*-acting RNA-binding proteins of the pentatricopeptide repeat (PPR) family. PPR proteins are organized in an array of repeats of ∼35 amino acids that interact with the target RNA in a modular one repeat:one nucleobase fashion (Lurin et al. 2004; Barkan et al. 2012). The criteria for the annotation of PPR motifs have been redefined several times since the discovery of PPR proteins (Aubourg et al. 2000; Small and Peeters 2000; Lurin et al. 2004; Cheng et al. 2016). Initially, PPR motifs were classified based on length: “pure” P-type motifs are comprised of 35 amino acids, “longer” L-type motifs extend up to 37 amino acids and “shorter” S-type motifs can vary in length between 31 and 34 amino acids (Lurin et al. 2004). Currently, PPR motifs are classified depending on the presence of conserved motif sequences rather than their sheer length, and are further distinguished into multiple subgroups (e.g., P1, P2; L1, L2, LL; S1, S2, SS; (Cheng et al. 2016; Gutmann et al. 2020)). In addition to the array of PLS-type motifs, which directly bind to the RNA, PPR proteins involved in RNA editing contain an extension at their C-terminus composed of the so-called E1, E2, and DYW domain. The DYW domain contains a conserved cytidine-deaminase signature that is essential for editing and harbors the deamination activity (Wagoner et al. 2015; Oldenkott et al. 2019; Hayes and Santibanez 2020; Takenaka et al. 2021). Some PPR editing factors lack the DYW domain (Chateigner-Boutin et al. 2008; Boussardon et al. 2012) and it is thought that in these cases, the editing activity can be provided in *trans* by small DYW-containing proteins (Boussardon et al. 2012; Andres-Colas et al. 2017; Guillaumot et al. 2017). The function of the E domains is still largely unclear. In some instances, the E motifs can interact with known non-PPR editing factors (e.g., multiple organellar RNA-editing factors/RNA-editing factor interacting proteins, dubbed MORFs or RIPs; reviewed in Sun et al. (2016)) or can affect binding affinity to positions adjacent to the edited cytidine (Ruwe et al. 2019). In addition to MORF/RIP proteins, several other non-PPR factors have been implicated in C-to-U RNA editing in seed plants (reviewed in Sun et al. (2016)). These include, but are not limited to, members of the organelle RRM protein (ORRM) and organelle zinc-finger (OZ) family. Non-PPR editing factors are required for editing at multiple sites, but their role in the hypothetical editosome is yet to be determined.

PPR proteins recognize RNA sequences upstream of the editing site. The amino acids in the fifth and last position of each P, L, or S-type motif jointly contact the RNA by forming hydrogen bonds with the aligned nucleobase (Barkan et al. 2012; Shen et al. 2016). Different amino acid combinations have different affinities towards specific bases. For example, a repeat with a threonine in position 5 and an asparagine in the final position preferentially binds to adenosine in the RNA. Therefore, it is possible to predict the RNA sequence recognized by a PPR protein based on its amino acid sequence and motif organization (Barkan et al. 2012; Takenaka et al. 2013; Yagi et al. 2013; Shen et al. 2016; Kobayashi et al. 2019; Yan et al. 2019). This “PPR code”, however, is rather degenerated and only partially explains experimental observations (Oldenkott et al. 2019). L-type motifs, for instance, cannot be reliably predicted using the fifth and last position alone (Takenaka et al. 2013), suggesting that other determinants are involved in target recognition or that these motifs might not directly participate in binding (Yan et al. 2017). A three-amino acid code, that additionally incorporates position 2, was shown to improve binding prediction (Takenaka et al. 2013). However, whether all L-type motifs interact with the target RNA and follow any particular code is still unclear.

In general, PPR editing factors in seed plants are highly specific in that they recognize a single site or at most a few sites, if those share similar *cis*-elements. Examples of PPR editing factors that target more than one cytidine in the chloroplast transcriptome are CLB19 (Chateigner-Boutin et al. 2008), CRR28 (Okuda et al. 2009), OTP82 (Hammani et al. 2009), CRR22 (Okuda et al. 2009), OTP84 (Hammani et al. 2009), VAC1 (Tseng et al. 2010), AEF1 (Hein et al. 2016), and QED1 (Wagoner et al. 2015). *Arabidopsis* QED1 (previously known as OTP81; (Hammani et al. 2009)) recognizes as many as five target sites in chloroplasts. Editing at three of the targets causes non-synonymous changes: editing at *ndhB*-291 and *rpoB*-811 converts serine into leucine codons, and editing at *matK*-214 converts a histidine into a tyrosine-encoding triplet. The other two target sites are located in the 3´ untranslated region of *accD* (*accD*_3UTR) and the first intron of *rps12* (*rps12*_i1). Whether these editing events have any effect on structure or folding of the 3´ UTR of *accD* or *trans*-splicing of the *rps12* intron, has not been investigated so far. Site *rps12*_i1 lies within an unstructured loop of Domain I and is not expected to impact the folding of this group II intron (supplementary fig. S1, Supplementary Material online). Among the five targets, only *matK*-214 and *rpoB*-811 are present and edited in non-Brassicaceae species ((Tillich et al. 2009) and this study). Non-coding regions are affected by higher mutation rates and can largely differ even between species of the same family. Therefore, restriction of editing at the *accD*_3UTR and *rps12*_i1 sites to Brassicaceae can be a consequence of the absence of the sequence requirements for editing in non-Brassicaceae species. By contrast, the sequence surrounding the *ndhB*-291 target, including the predicted binding site of QED1, is highly conserved in flowering plants. An “editable” cytidine is present at the analogous position in several other angiosperms, but editing of this C was not detected in any species outside the Brassicaceae family. It is furthermore remarkable that editing of *ndhB*-291 causes a missense mutation in that a serine codon is encoded at this position in almost all species of monocotyledonous and dicotyledonous plants (with the only exceptions among the 3,683 spermatophytes species with available *ndhB* sequences in the CpGDB-database (Singh et al. 2020) being *Chimonanthus nitens*, *Carex neurocarpa*, *Carex siderosticta,* and *Lens culinaris*; see supplementary Materials and Methods and supplementary table S1, Supplementary Material online). In Brassicaceae, RNA editing converts the conserved serine codon to a leucine codon (Tillich et al. 2005). Why editing at *ndhB*-291 site occurs, and why it is nearly completely restricted to the Brassicaceae family is enigmatic.

In this study, we investigated the evolution of the unusual *ndhB*-291 site and its corresponding PPR protein, QED1. Our data suggest that *ndhB*-291 is the first case of a recently gained editing site in seed plant chloroplasts, and that the ability to process this site is specific to the *Arabidopsis* QED1 protein. By constructing chimeric versions of the QED1 protein, we show that changes in the PPR motifs of *Arabidopsis* QED1 allow recognition of *ndhB*-291 in a way that is not predictable by the current PPR code. Moreover, we detected multiple off-targets edited by QED1 in the organellar transcriptomes of transgenic QED1-expressing tobacco plants. By manipulating the expression level of QED1 and one of its targets (*ndhB*-291), we uncovered that the target specificity of PPR proteins is strongly dependent on the RNA:protein stoichiometry. We propose that the expression of PPR-type editing factors needs to be kept to low levels to ensure site specificity and avoid deleterious off-target editing of the transcriptome.

## Results

### PPR Protein QED1 and RNA-Editing Site *ndhB*-291 are Widespread in Embryophyta, but Editing at *ndhB*-291 is Restricted to Brassicaceae

**Fig. 1.**
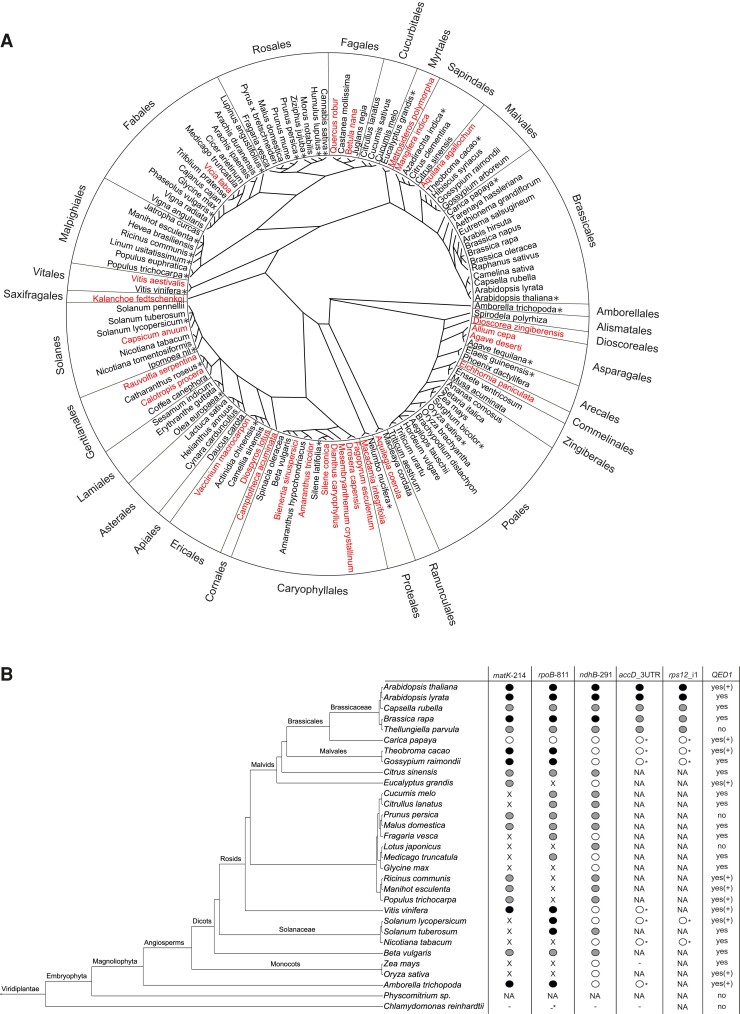
Phylogenetic distribution of *QED1* and its targets in Viridiplantae. (*A*) Phylogenetic analysis of putative *QED1* orthologs (see Materials and Methods for details). Phylogenetic analysis was performed as in Oldenkott et al. (2020). *: expression supported by hits in the OneKP dataset (One Thousand Plant Transcriptomes Initiative 2019); red: no QED1 ortholog hit found. Species and their phylogenetic information (including plant orders) are as in (Oldenkott et al. 2020). Tree was drawn with the plol.phylo function, radial layout, R package “ape” (Paradis and Schliep 2019). Complete information is provided in supplementary table S2, Supplementary Material online. (*B*) Information on the editing status of the *matK*-214, *rpoB*-811, *ndhB*-291, *accD*_3UTR, and *rps12*_i1 sites and the presence of *QED1* orthologs from 31 selected Viridiplantae species. Data were compiled from Kahlau and Bock 2008; Tillich et al. 2009; Ishibashi et al. 2019, and this work. Circle-fill-color-code as in supplementary table S2, Supplementary Material online. Black circle: C-to-U conversion, confirmed by Sanger sequencing and/or NGS data; white circle: unedited C, confirmed by Sanger sequencing and/or NGS data; grey circle: cytidine encoded in the DNA, no information about the editing status; X: thymidine encoded in the DNA; NA: no information, no sequence or no hit to editing site sequence (via blast); white circle*: region sequenced, although not conserved, and no editing observed; -: gene absent from the plastid genome; -*: *C. reinhardtii* encodes two closely linked reading frames (*rpoB*-1 and *rpoB*-2); yes: *QED1* ortholog identified; yes(+): *QED1* ortholog expressed; no: no *QED1* ortholog. Presence/absence of *QED1* orthologs is reported as in panel *A*. The phylogenetic tree was manually drawn as shown in PLAZA 3.0 (Proost et al. 2015) using TreeGraph 2.0.47 (Stover and Muller 2010) to represent the taxonomy.

To investigate the phylogenetic distribution of QED1, we searched for QED1-like sequences in publicly available genomic and transcriptomic datasets (see Materials and Methods for details; (Van Bel et al. 2018; Carpenter et al. 2019; One Thousand Plant Transcriptomes Initiative 2019)). After a careful selection of only high-quality sequencing data, we identified orthologous genes of *Arabidopsis QED1* in 97 species covering a wide range of angiosperm phylogeny (fig. 1*A* and supplementary table S2, Supplementary Material online). Remarkably, a putative ortholog of QED1 was found to be expressed in *Amborella trichopoda* (fig. 1*A* and supplementary table S2, Supplementary Material online), a basal angiosperm species that is believed to be sister to all other angiosperms (Amborella Genome Project 2013). Interestingly, *Nicotiana tabacum* appears to encode a QED1 ortholog in its nuclear genome, but no expression could be detected in either the One Thousands Plant Transcriptome (One Thousand Plant Transcriptomes Initiative 2019) or in-house next-generation sequencing (NGS) data (POTbase; (Moreno et al. 2018)). QED1 orthologs were identified exclusively in angiosperm species, supporting recent reports that PPR editing factors are phylogenetically restricted to individual clades (Gutmann et al. 2020).

PPR-type editing factors are known to tightly co-evolve with their corresponding target(s) in the organellar genomes (Rudinger et al. 2011; Hayes et al. 2012; Hein et al. 2016; Loiacono et al. 2019). Single-target editing factors generally degenerate from the nuclear genome after loss of the editing site (i.e., reversion to a genomic T in the plastid genome; (Rudinger et al. 2011; Hayes et al. 2012; Hein et al. 2016; Loiacono et al. 2019)). By contrast, multiple-target editing PPRs such as CLB19 and CRR28, which target two distinct sites each (Chateigner-Boutin et al. 2008; Okuda et al. 2009), are often maintained across larger phylogenetic distances, because they usually become dispensable only if all target sites are lost (Hein and Knoop 2018). The PPR protein QED1 is unique in that it edits as many as five sites in *Arabidopsis* chloroplasts, which makes it exceptionally well suited to investigate the co-evolution of a PPR protein with its target sites. We, therefore, analyzed the distribution of QED1 targets in the green plant clade (Viridiplantae) using publicly available genomic and transcriptomic datasets (see Materials and Methods; fig. 1*B* and supplementary table S2, Supplementary Material online). Two of the QED1 targets affect coding sequences of essential genes: *matK*-214 and *rpoB*-811. Both *matK*-214 and *rpoB*-811 are present and edited in *Amborella trichopoda*, in agreement with the presence of a functional QED1 ortholog identified by our phylogenetic analysis (fig. 1*A*and*B*). The phylogenetic distribution of *matK*-214 and *rpoB*-811 suggests that these two sites (along with QED1) may have appeared early in the evolution of angiosperms following independent losses in several dicotyledonous lineages. Reversion of both the *matK*-214 and the *rpoB*-811 site to genomic Ts is generally accompanied by the loss of a confident *QED1* gene (supplementary table S2, Supplementary Material online). Notably, *N. tabacum* is among those species that harbor a T in the DNA at the positions corresponding to *matK*-214 and *rpoB*-811, but yet encode a QED1-like protein (although the loss of these two editing sites should make it dispensable). However, the putative QED1 ortholog identified in *N. tabacum* shares only 42% identity with *Arabidopsis* QED1, and the tobacco gene seems not to be expressed, as evidenced by the absence of reads from both the One Thousands Plant Transcriptome database (One Thousand Plant Transcriptomes Initiative 2019) and our in-house next-generation sequencing (NGS) datasets (POTbase; (Moreno et al. 2018)). By contrast, the closely related Solanaceous species tomato (*Solanum lycopersicum*) and potato (*Solanum tuberosum*) possess QED1 orthologs that are 61% similar to *Arabidopsis*. In addition, in a previous study that made use of cybrids generated by combining the nuclear genome of *N. tabacum* and the plastome of the deadly nightshade *Atropa belladonna*, it was shown that tobacco does not possess the editing activity for *rpoB*-809 (the *A. belladonna* homolog of the *Arabidopsis rpoB*-811 site), in that the site remained unedited in the cybrid (Schmitz-Linneweber et al. 2005). Together, all this evidence suggests that the QED1 ortholog identified in *N. tabacum* by our bioinformatic pipeline is a non-functional pseudogene.

Two other editing sites targeted by *Arabidopsis thaliana* QED1, *accD*_3UTR and *rps12*_i1, are located within a 3' UTR (position *accD*_C1568) and a group II intron (position *rps12*_i1C58), respectively, and are only edited to ∼50–60% and ∼10–30%, respectively (Wagoner et al. 2015). Intergenic sequences often differ substantially even between closely related species. Additionally, *rps12* intron 1 is encoded by two separated loci and excised by *trans*-splicing. The exact termini of the two *trans*-spliced RNA pieces have been mapped only in *Arabidopsis* (Aryamanesh et al. 2017). We selected a subset of representative angiosperm species to determine if the *accD*_3UTR and *rps12*_i1 sites are edited in species other than *Arabidopsis*. Based on sequence alignments (supplementary figs. S2 and S3, Supplementary Material online) and targeted Sanger sequencing, both editing sites appear to be restricted to the Brassicaceae branch (fig. 1*B*). However, the low conservation of the upstream *cis*-elements of *accD*_3UTR and *rps12*_i1 does not allow us to confidentially reconstruct their phylogenetic distribution in Viridiplantae.

**Fig. 2.**
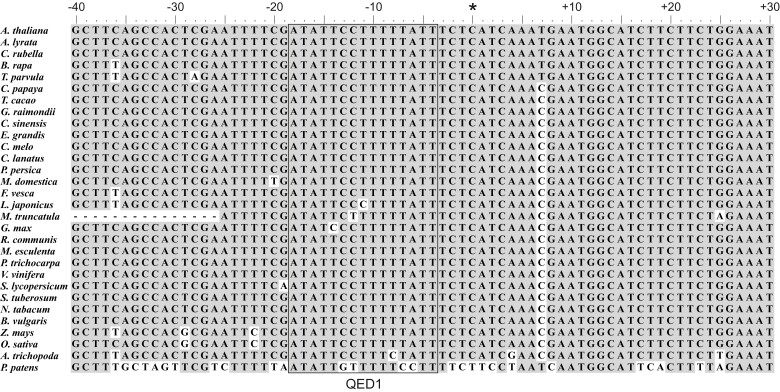
Alignment of sequences harboring the *cis*-element for *ndhB*-291 editing. The alignment represents the sequence surrounding the *ndhB*-291 site (marked by an asterisk) from position −40 to +30. The predicted binding site of QED1 (with the last S2 motif aligned to position −4 with respect to the editing site) is boxed. Positions identical to those of the *A. thaliana* sequence are shaded in grey.

The fifth editing site recognized by *Arabidopsis* QED1, *ndhB*-291, also appears to be specific to the Brassicaceae (fig. 1*B* and supplementary table S2, Supplementary Material online). The sequence surrounding *ndhB*-291 is strikingly conserved, with only minor differences between *Arabidopsis* and *Amborella*, which are separated by more than 100 million years of evolution (fig. 2). Among the species investigated, *ndhB*-291 is edited only in the Brassicaceae, although the site is widespread in angiosperm evolution (fig. 1*B* and 2).

To identify differences that potentially could explain why only the QED1 proteins of Brassicaceae are capable of editing *ndhB*-291, orthologs identified by our pipeline were submitted to the PPR Gene Database (Cheng et al. 2016) for prediction of PPR motifs and RNA-binding sites. We selected only those orthologs that shared at least 55% sequence identity with *Arabidopsis* QED1. We set this threshold based on the sequence identity of *Amborella* QED1 (55%), that we consider the most ancient angiosperm ortholog in our dataset. At the level of domain structure, most QED1 proteins are identical to *Arabidopsis* QED1 in number and type of motifs, and show the same basic structure of 15 PPR motifs (L1-S1-P1-L1-S1-P1-L1-S1-SS-P1-L1-S1-P2-L2-S2) and a C-terminal region composed of the E1, E2, and DYW domains (supplementary table S3, Supplementary Material online). The fifth and last positions of each PPR motif (formerly known as positions 6 and 1´, respectively) directly interact with the RNA target and define which nucleobase is preferentially recognized. Given the nearly universally conserved *cis*-element of *ndhB*-291 (fig. 2), it seems possible that changes in the PPR motifs and, consequently, in the RNA-binding affinity of QED1 facilitated the recognition and editing of *ndhB*-291 in Brassicaceae. Although some differences in the fifth and last positions can be identified between Brassicaceae and non-Brassicaceae (supplementary table S3, Supplementary Material online), they result in only minor changes in the predicted binding site and cannot be correlated to a better recognition of *ndhB*-291 by the QED1 of Brassicaceae.

**Fig. 3.**
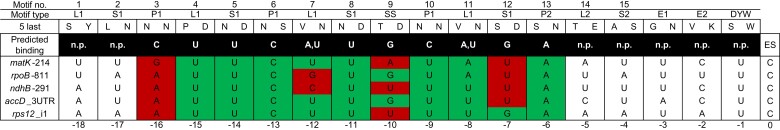
*Cis*-elements of *Arabidopsis* QED1 targets and their recognition by PPR motifs. The putative *cis*-elements of the five *Arabidopsis* targets of QED1 are aligned. The editing site (ES) represents position 0. Each position was evaluated with respect to binding of *Arabidopsis* QED1 based on the PPR code ((Cheng et al. 2016); supplementary table S3, Supplementary Material online). Amino acids responsible for binding (5th and last) of each PPR repeat are shown in the row “5 last”. The final PPR motif corresponds to position −4. Green: match with the code; red: mismatch; n.p.: not predictable.

By aligning the *cis*-elements of all *Arabidopsis* targets of QED1, we identified two positions that are specific to *ndhB*-291. A cytidine at position −12 (editing site: position 0) is only present upstream of *ndhB*-291, while the same position is occupied by a guanine in *rpoB*-811 and a uracil in *matK*-214, *accD*_3UTR and *rps12*_i1 (fig. 3). Similarly, a uracil is found at position −10 upstream of *ndhB*-291 (and *rps12*_i1), but not in the other sites, where guanine (*rpoB*-811 and *accD*_3UTR) or adenine (*matK*-214; fig. 3) are present at position −10. The nucleotides at position −12 and −10 align to PPR motifs 7 (L1) and 9 (SS) of QED1, respectively. According to the PPR code (Cheng et al. 2016), the VN (valine/asparagine) combination in the L1 motif is predicted to recognize adenine or uracil, but not cytosine, while the TD (threonine/aspartate) pair in SS is predicted to specifically bind to guanine. Interestingly, species closely related to the Brassicaceae, such as *Theobroma cacao* (fig. 1), encode the same amino acids at these positions as *Arabidopsis*, but do not edit *ndhB-291*, despite the presence of an editable C (fig. 1). Therefore, if PPR motifs 7 and/or 9 underwent changes in the Brassicaceae to accommodate the specific nucleotide residues present upstream of *ndhB*-291, these changes do not involve (or are not restricted to) the fifth and final positions of the motifs.

Consequently, the current version of the PPR code (Cheng et al. 2016) does not explain why QED1 edits the *ndhB*-291 site only in the Brassicaceae, despite the high sequence similarity of the *cis*-element of *ndhB*-291 with the *cis*-elements of the other QED1 target sites and the widespread presence of a conserved QED1 gene in all major eudicotyledonous clades.

### *Arabidopsis* QED1 Fully Edits Site *ndhB*-291 in Tobacco

To test if the editing capacity for *ndhB*-291 is associated with the QED1 protein, *Arabidopsis* QED1 and its close ortholog from *Theobroma cacao* were stably expressed in tobacco (*N. tabacum*) plants. Tobacco encodes a C at *ndhB*-291, but does not edit it. The possible *QED1* ortholog harbored in the tobacco nuclear genome is very likely non-functional (see above). *Theobroma cacao* belongs to the Malvales, a sister clade of the Brassicales, and is the closest non-Brassicaceae species that contains a functional *QED1* gene. Cacao QED1 edits the endogenous sites *matK*-214 and *rpoB*-811, but not *ndhB*-291 (fig. 1*B*), even though the protein is identical in its PPR structure to the *Arabidopsis* QED1 (supplementary tables S2 and S3, Supplementary Material online).

**Fig. 4.**
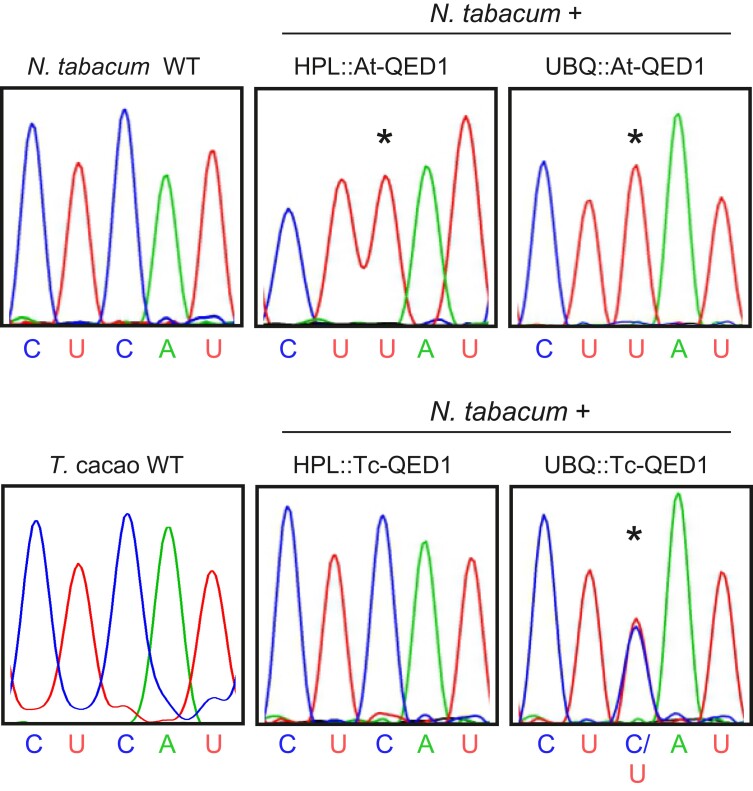
Editing activity of *Arabidopsis* and cacao QED1 on *ndhB*-291 in tobacco chloroplasts. The editing status of *ndhB*-291 was assessed by sequencing of the amplified cDNA population from *N. tabacum* wild type (WT), *T. cacao* wild type and transgenic tobacco plants expressing the *QED1* gene from *Arabidopsis* (At-QED1) or cacao (Tc-QED1) under the *HYDROPEROXIDE LYASE1* (HPL) or *UBIQUITIN10* (UBQ) promoter. C-to-U conversion at the *ndhB*-291 site is marked by asterisks.

Previously, we showed that the relatively weak promoter from the *HYDROPEROXIDE LYASE1* (*HPL*) gene and the strong promoter from the *UBIQUITIN10* (*UBQ*) gene can be used to express an editing PPR from *Arabidopsis* to reconstitute heterologous editing at a tobacco site (Loiacono et al. 2019). The same promoters were used to express the QED1 orthologs in tobacco. Generation of stable QED1-expressing transgenic tobacco plants revealed that the *Arabidopsis* QED1 (At-QED1) triggered editing of the tobacco *ndhB*-291 site irrespective of the promoter used (fig. 4). Importantly, in all independent transgenic HPL::At-QED1 (*n* = 8) and UBQ::At-QED1 (*n* = 3) lines analyzed, *ndhB*-291 was always fully edited (100% C-to-U conversion). By contrast, expression of the *T. cacao* QED1 (Tc-QED1) did not result in any detectable C-to-U conversion at *ndhB*-291 when the *HPL* promoter was used for transgene expression (HPL::Tc-QED1, *n* = 5; fig. 4). However, partial editing of *ndhB*-291 (∼50% C-to-U conversion) was detected upon transgene expression from the strong *UBQ* promoter (UBQ::Tc-QED1, *n* = 3; fig. 4). Thus, cacao QED1 seems to possess, to a certain extent, the ability to edit *ndhB*-291 in tobacco. However, the cacao ortholog is likely less active on this site than the *Arabidopsis* QED1.

In summary, the *Arabidopsis* QED1 protein fully edits the *ndhB*-291 site in *Arabidopsis* chloroplasts and, upon heterologous expression (and independent of the promoter used to drive the transgene), also fully edits the normally unedited *ndhB*-291 site in tobacco plastids. Cacao QED1 does not naturally edit *ndhB*-291 to detectable levels, but partial editing at this site in tobacco can be induced by expression from the strong *UBQ* promoter (fig. 4).

### Changes in the PPR Motifs of QED1 Facilitate Recognition of *ndhB*-291

Since our bioinformatic analysis did not hint at a specific PPR motif(s) that could be associated to the competence of editing *ndhB*-291, we decided to generate a set of chimeric QED1 versions. To this end, distinct parts of the cacao QED1 were replaced by the corresponding fragments from *Arabidopsis*. Swapped parts included the C-terminus (comprising the E and DYW domains) and the PPR tract. The PPR array was further divided into two fragments comprising the first eight and the last seven PPR motifs. To exclude possible effects of the chloroplast targeting signals, the N-terminal region upstream of the first predicted PPR motif was replaced in all constructs by the transit peptide of the plastid-targeted small subunit of RuBisCO (*RBCS*) followed by a short glycine linker (GGG). It should be noted that these constructs were generated before the most recent PPR annotation was released (Cheng et al. 2016) and, therefore, were designed based on the previous version of PPR annotation (Lurin et al. 2004). The chimeric constructs were stably expressed from either the moderate *HPL* or the strong *UBQ* promoter in the genetic background of the *qed1-2 Arabidopsis* knock-out mutant, in which editing at all five QED1 targets is abolished (Wagoner et al. 2015). Complementation of the editing defect at *ndhB*-291 is expected to occur only when the chimeric cacao protein carries the motif(s) from the *Arabidopsis* protein, which are required for recognition of *ndhB*-291. By testing the chimeric QED1 variants in the *Arabidopsis* knock-out background, we could control for the activities of all designed PPRs by assessing the editing status at the other four sites (*matK*-214, *rpoB*-811, *accD*_3UTR, and *rps12*_i1). The editing data obtained for all chimeric constructs are listed in supplementary table S4, Supplementary Material online and summarized in table 1.

**Table 1.** Editing Activities of Chimeric QED1 Variants Tested in the *Arabidopsis qed1* Mutant.

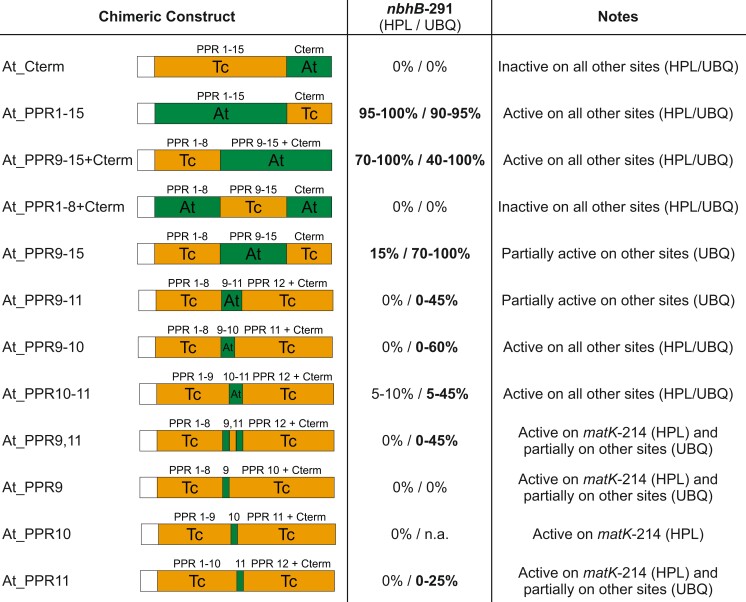					

The table summarizes the complementation of QED1 targets in the *Arabidopsis qed1* mutant by chimeric variants of QED1. A schematic representation of each construct used to generate transgenic plants is shown in the first column. Parts of the *T. cacao* (Tc; orange boxes) QED1 protein were replaced by the corresponding fragments from *A. thaliana* (At; green boxes). Construct names refer to the *Arabidopsis* portion contained in each chimeric QED1 variant. PPR motifs are indicated as predicted by Cheng et al. (2016). Note that constructs At_Cterm, At_PPR1-15 + Cterm, At_PPR9-15 + Cterm, At_PPR1-8 + Cterm and At_PPR9-15 were designed based on the PPR annotation from Lurin et al. (2004). C-term comprises the E (E1/E2) and DYW motifs. In all constructs, the N-terminal sequence upstream of the first annotated PPR motif was replaced by the transit peptide of the plastid-imported small subunit of RuBisCO (*RBCS*) followed by the short glycine linker GGG (white boxes). Each chimera was expressed from the *HPL* or the *UBQ* promoter in the *qed1-2* knock-out background. The editing status of *ndhB*-291 was assessed by bulk sequencing of cDNA or iPLEX/MassARRAY® (complete data are shown in supplementary table S4, Supplementary Material online). The editing efficiency (determined as C-to-U conversion rate) at *ndhB*-291 is given in % (in bold). n.a.: no transgenic plants were obtained for At_PPR10 expressed from the *UBQ* promoter.

Editing at *ndhB*-291 was completely restored when the chimera harboring the PPR tract of *Arabidopsis* QED1 and the C-terminus of the cacao protein (At_PPR1-15) was expressed from the moderate *HPL* promoter, indicating that the changes responsible for the gain of *ndhB* editing activity are associated with the PPR tract. When the first eight PPR motifs from the cacao protein were retained, the editing defect at *ndhB*-291 was still fully rescued, provided that the C-terminal E and DYW domains were also derived from *Arabidopsis* (At_PPR9-15 + Cterm). Interestingly, the presence of the C-terminus from cacao downstream of the *Arabidopsis* PPR motifs 9–15 strongly reduced editing activity for *ndhB*-291 upon expression from the *HPL* promoter but not upon overexpression from the *UBQ* promoter (At_PPR9-15). Importantly, all constructs that restored editing at *ndhB*-291 also complemented the editing defects of the *qed1-2* mutant at the other four targets (supplementary table S4, Supplementary Material online), demonstrating that these chimeric proteins are capable of supplying full editing capacity.

The results described above suggest that the ability to edit *ndhB*-291 correlates with the presence of PPR motifs 9–15 of the *Arabidopsis* QED1 protein. Consistent with this interpretation, transgenic lines generated with chimeric constructs that carry either the whole PPR tract (At_Cterm) from cacao or only the last seven PPR motifs of the cacao QED1 (At_PPR1-8 + Cterm) failed to edit *ndhB*-291 when expressed from either the moderate *HPL* or the strong *UBQ* promoter (table 1). Surprisingly, these constructs also failed to edit any other QED1 target (supplementary table S4, Supplementary Material online). In the absence of specific antibodies, it is currently not possible to investigate the expression of these chimeras at the protein level, to rule out the possibility that the fusion between the PPR tract of cacao and the C-terminus of the *Arabidopsis* QED1 results in protein instability. However, we propose that the At_Cterm and At_PPR1-8 + Cterm proteins are non-functional due to differences in the E domain (supplementary fig. S4, Supplementary Material online) revealed by the most recent PPR motif annotation (Cheng et al. 2016) and recent domain-swapping experiments (Ichinose and Sugita 2018).

To further narrow down on the region within the *Arabidopsis* QED1 that confers to the cacao protein the competence to edit *ndhB*-291, we generated an additional set of constructs. Based on the observations that changes responsible for the gain of function of *ndhB*-291 editing lie within motifs 9 and 15 and that SS motif 9 aligns to one of the only two positions specific to *ndhB*-291, we selected the triplet SS-P1-L1 (motif 9, 10, and 11) as candidate for the substitution strategy. Similar to the previous set of chimeras, the cacao motifs 9, 10, and 11 were replaced by the corresponding PPR repeats from *Arabidopsis.* This new set of constructs was generated based on the most recent PPR annotation (Cheng et al. 2016). As the previous set, all chimeric constructs were stably expressed from the *HPL* and *UBQ* promoters in the *qed1-2 Arabidopsis* knock-out background. Surprisingly, none of the *HPL* constructs resulted in detectable editing at *ndhB*-291 and the degree of complementation at other QED1 target sites varied substantially between individual transgenic lines (table 1 and supplementary table S4, Supplementary Material online). When expressed from the *UBQ* promoter, chimeras harboring the complete triplet (At_PPR9-11) or the three possible combinations of repeat pairs from *Arabidopsis* (At_PPR9-10, At_PPR10-11, and At_PPR9,11) were able to at least partially edit *ndhB*-291 (up to ∼60% C-to-U conversion). The replacement of single motifs (At_PPR9, At_PPR10, At_PPR11) did not restore editing, with the exception of motif 11 from *Arabidopsis* that resulted in low-level complementation of the *ndhB*-291 editing defect (of up to ∼25%; table 1 and supplementary table S4, Supplementary Material online).

In conclusion, the large set of chimeric constructs tested in this work by stable transformation shows that, although PPR proteins are believed to function in a modular fashion, our current knowledge is insufficient to design fully functional chimeric PPRs with altered target site specificities. Nonetheless, we succeeded with reprogramming the cacao QED1 to edit *ndhB*-291 by introducing specific motifs from *Arabidopsis*. Our data suggest that the triplet SS-P1-L1 (PPR motifs 9, 10, and 11) is involved in the recognition of *ndhB*-291 by QED1.

### Overexpression of *Arabidopsis* QED1 Causes a Severe Growth Phenotype in Tobacco

**Fig. 5.**
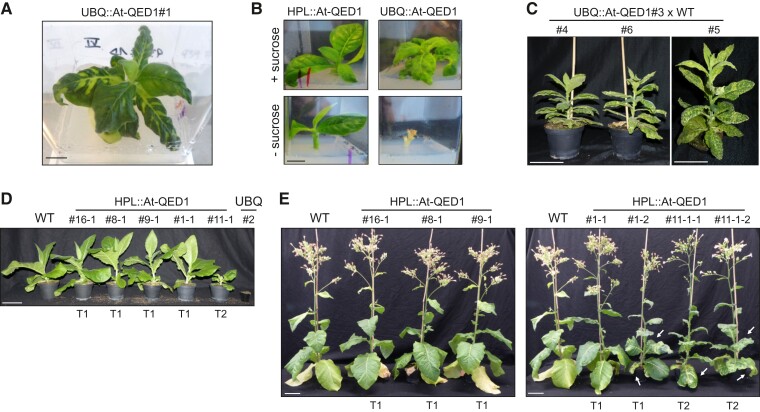
Phenotype of transgenic tobacco lines stably expressing the *Arabidopsis* QED1 protein. (*A*) A UBQ::At-QED1 plant growing in aseptic conditions on sucrose-containing medium. Scale bar: 1 cm. (*B*) Test for autotrophic growth of HPL::At-QED1 and UBQ::At-QED1 lines. Plants were grown in the presence (+) or the absence (−) of sucrose in the medium. Scale bar: 1 cm. (*C*) Phenotype of the progeny of UBQ::At-QED1#3 crossed to wild type (WT) photographed 18 weeks after sowing. Scale bars: 13 cm. (*D*) Phenotype of wild-type tobacco (WT) and the progeny (T1 or T2 generation) of five independent HPL::At-QED1 lines and one UBQ::At-QED1 (UBQ) line grown under standard greenhouse conditions and photographed six weeks after sowing. Note that the UBQ::At-QED1 plant did not survive in soil under these conditions. Scale bar: 13 cm. (*E*) Phenotype of a wild-type tobacco plant (WT) and the progeny (T1 or T2 generation) of HPL::At-QED1 lines 11 weeks after sowing. The progeny of lines #16, #8, and #9 (left panel) was indistinguishable from the wild type. Line #1 segregated in wild-type-like (#1–1) and variegated plants (#1–2) in the T1. The progeny of the variegated HPL::At-QED1#11 exhibited a strong leaf variegation (#11–1–1 and #11–1–2). Arrows indicate variegated leaves. Scale bars: 13 cm.

Editing at the *ndhB*-291 site could be induced in tobacco by expression of the *Arabidopsis* PPR protein QED1. Thus, the QED1 PPR protein and the *ndhB*-291 site represent another example of a successful transfer of editing activities between species (Okuda et al. 2009; Loiacono et al. 2019; Oldenkott et al. 2020). In the case of the *Arabidopsis* LPA66 protein and editing site *psbF*-26 (Loiacono et al. 2019), expression of the heterologous PPR in tobacco restored a wild type-like phenotype, fully complementing the photosynthetic defect associated with the unedited *psbF*-26 site. Importantly, even overexpression of *Arabidopsis* LPA66 produced transgenic tobacco plants that were indistinguishable from the wild type (Loiacono et al. 2019). By contrast, strong expression of *Arabidopsis* QED1 caused a severe mutant phenotype. When the transgene was expressed from the *UBQ* promoter, the transgenic tobacco plants displayed variegated leaves and were retarded in growth. When primary transformants were propagated under aseptic conditions, all UBQ::At-QED1 lines (*n* = 3) were significantly retarded in growth compared to the HPL::At-QED1 lines (*n* = 8). Also, UBQ::At-QED1 transformants developed variegated leaves with patches of interveinal areas turning pale-green (fig. 5*A*). When transferred to soil and grown under standard greenhouse conditions (12 h photoperiod, 20°C day/18°C night, 55% humidity), the overexpression lines did not survive. Similarly, these lines did not develop roots when grown in aseptic conditions in the absence of sucrose in the medium (fig. 5*B*). In conclusion, the UBQ::At-QED1 lines were incapable of growing autotrophically and had to be maintained in aseptic conditions on sucrose-containing medium. After intensive efforts, one of the three UBQ::At-QED1 lines (UBQ::At-QED1#3) could be maintained in soil for an extended period of time in permissive conditions (shaded, natural photoperiod, 20°C day/18°C night, 60% day/50% night humidity). However, the plants grew extremely slowly and did not produce seeds from self-pollination. Interestingly, when crossed to the wild type, the kanamycin-resistant progeny of UBQ::At-QED1#3 was severely variegated and retarded in growth, but survived in soil under standard greenhouse conditions (UBQ::At-QED1#3×WT; fig. 5*C*). UBQ::At-QED1#3 is, therefore, considered a “weak” UBQ line, since it can grow autotrophically in permissive conditions or when crossed with the wild type (which, for unknown reasons, may result in somewhat lower transgene expression levels in the progeny).

When transferred to soil in standard greenhouse conditions, HPL::At-QED1 primary transformants were viable and produced seeds. The progeny of six out of eight obtained lines uniformly displayed a wild type-like phenotype (HPL::At-QED1#6, #8 and #9 in fig. 5*D*and*E*). Two lines, HPL::At-QED1#1 and #11, segregated into wild type-like plants and plants that showed pale-green variegated leaves (HPL::At-QED1#1 in fig. 5*E*). The next generation from one of the variegated HPL segregants was significantly retarded in growth early in development compared to the wild type and the other HPL lines (HPL::At-QED1#11-4 T2; fig. 5*E*). However, upon continued growth, the plants recovered, became similar in size to wild-type plants, but still exhibited severe leaf variegation (fig. 5*E*).

In summary, expression of the *Arabidopsis* PPR protein QED1 in tobacco affects plant growth and development. The severity of the phenotype depends on the promoter used to drive the transgene, suggesting an inverse relationship between QED1 expression levels and plant growth.

### Moderate Expression of *Arabidopsis* QED1 Affects Accumulation of the Cytochrome *b_6_f* Complex

The leaf variegation phenotype observed in the UBQ::At-QED1 and, to a lesser extent, in the HPL::At-QED1 lines, suggested that the expression of *Arabidopsis* QED1 may interfere with chloroplast function.

**Fig. 6.**
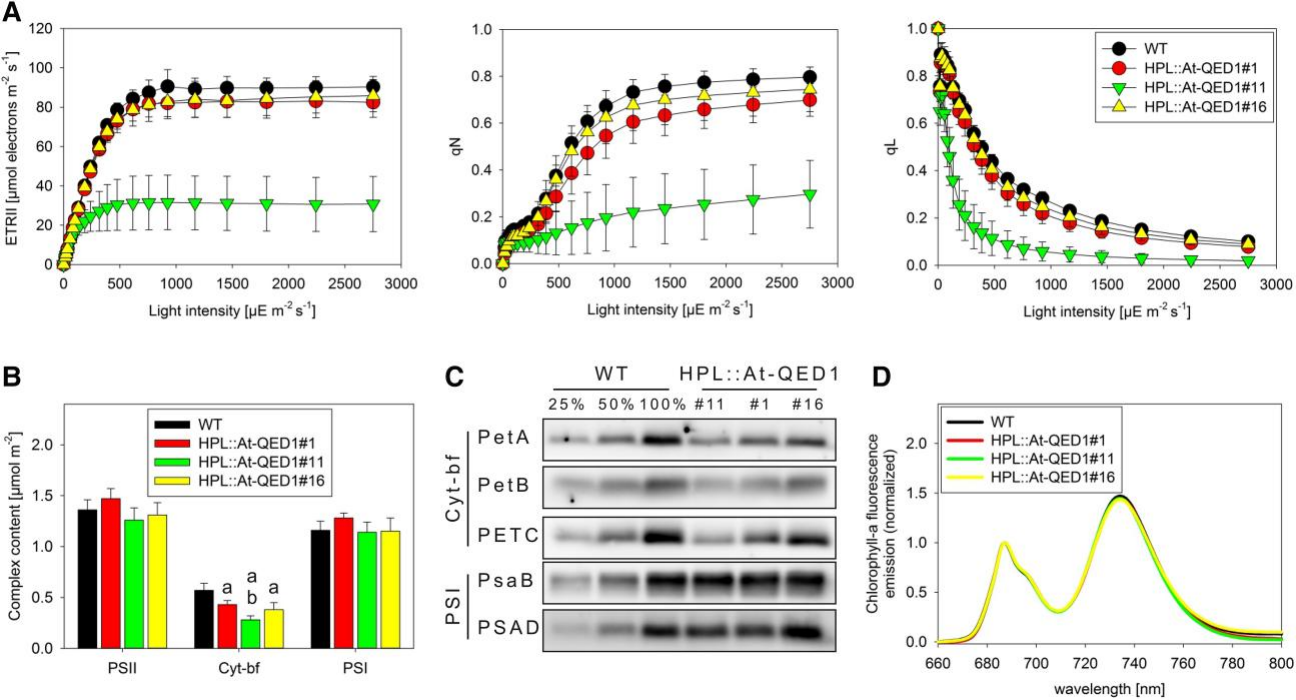
Status of the photosynthetic apparatus in HPL::At-QED1 transgenic plants. (*A*) *In vivo* measurements of the electron transport rate (ETR), the non-photochemical quenching capacity (qN), and the fraction of open PSII centers (qL). Error bars indicate the standard deviation of the biological replicates indicated in supplementary table S5, Supplementary Material online. (*B*) *In vitro* complex content quantification in purified thylakoids for photosystem II (PSII), the cytochrome *b_6_f* complex (Cyt-bf) and photosystem I (PSI). Error bars indicate the standard deviation of the biological replicates indicated in supplementary table S5, Supplementary Material online. a = significantly different between wild type and mutants; b = significantly different between mutant lines. One-way ANOVA, Holm–Sidak method, *P* ≤ 0.05. (*C*) Immunoblot analyses with antibodies against diagnostic subunits of cytochrome *b_6_f* (Cyt-bf; PetA, PetB, and PETC) and PSI (PsaB and PsaD). Thylakoid extracts were loaded on equal chlorophyll basis. For semiquantitative assessment, a dilution series of the wild-type sample was loaded (25%, 50%, and 100%). (*D*) 77*K* chlorophyll-*a* fluorescence emission measurements normalized to PSII emission at 686 nm.

To characterize the mutant phenotype of the QED1 transformants in more detail, a number of photosynthetic parameters were measured (see Materials and Methods) in two independent HPL::At-QED1 lines, both of which produced variegated individuals in the T1 generation (#1 and #11), and one HPL::At-QED1 line that produced a uniform wild type-like progeny (#16). Total chlorophyll content, chlorophyll-*a*/*b* ratio, leaf absorptance, thylakoid membrane conductivity for protons (a measure of chloroplast ATP synthase activity) and the maximum quantum efficiency of photosystem II in the dark-adapted state (*F*_v_/*F*_m_) were unaltered or only mildly affected in all HPL lines (supplementary table S5, Supplementary Material online). The light saturation curves of linear electron flux (ETR; fig. 6*A*) showed that the linear electron transport capacity was strongly reduced in HPL::At-QED1#11, being saturated already at light intensities below 500 μmol photons/(m^2^ s). The other two lines did not significantly differ from the wild type. Induction of non-photochemical quenching (qN) was severely impaired in HPL::At-QED1#11, suggesting a problem in generating a sufficient proton motive force across the thylakoid membrane to activate this photoprotective mechanism, possibly in line with the impaired linear electron transport. Only minor differences in qN were seen in HPL::At-QED1#1 and #16. The light response curve of qL is a measure for the redox state of the PSII acceptor side, with a value of one meaning that the PSII acceptor side is fully oxidized, while zero indicates its complete reduction. In HPL::At-QED1#11, the reduction of the PSII acceptor side was shifted to much lower light intensities compared to the wild type (fig. 6*A*), suggesting a strong limitation in electron transport downstream of PSII, while the other two lines were indistinguishable from wild type. Taken together, these data strongly resemble the behavior of knock-down mutants of the cytochrome *b_6_f* complex, which catalyzes the rate-limiting step of linear electron transport (Schottler et al. 2007; Hojka et al. 2014).

Therefore, we next quantified photosynthetic complexes in isolated thylakoid membranes using spectroscopic techniques, and then re-normalized complex contents to a leaf area basis (fig. 6*B*). These measurements revealed a specific and significant reduction of cytochrome *b_6_f* complex accumulation in all HPL mutants. The most affected line (HPL::At-QED1#11) possessed only about 50% of the cytochrome *b_6_f* amounts of the wild type (fig. 6*B*), whereas lines HPL::At-QED1#1 and #16 showed an approximately 25% and 35% reduction in cytochrome *b_6_f* content, respectively. Accumulation of both photosystems was unaltered in all HPL mutants relative to wild type. These results were confirmed by immunoblots against essential subunits of the cytochrome *b_6_f* complex and PSI (fig. 6*C*). Immunoblots were loaded based on equal amounts of chlorophyll, but because chlorophyll content per leaf area was very similar between the wild type and all HPL::At-QED1 lines (see above), data are directly comparable to the photosynthetic complex contents quantified on a leaf area basis. Accumulation of the two plastid-encoded subunits PetA and PetB was most strongly reduced in HPL::At-QED1#11, and this was also observed for the nucleus-encoded PETC subunit, the Rieske protein. For PSI, the plastid-encoded reaction center subunit PsaB and the nucleus-encoded subunit PSAD involved in ferredoxin reduction at the PSI acceptor side were tested, and both proteins accumulated to similar levels in the wild type and all three HPL mutants. Finally, in line with the unaltered photosystem contents, 77K chlorophyll-*a* fluorescence measurements revealed very similar emission spectra of the HPL::At-QED1 lines and the wild type (fig. 6*D*), suggesting unaltered antenna sizes of both photosystems in the mutants. Therefore, all photosynthetic data point to a highly specific defect in the accumulation of the cytochrome *b_6_f* complex in the HPL mutants, with the most severe defects occurring in line #11.

### Overexpression of *Arabidopsis* QED1 Does not Affect Editing at Endogenous Sites in Tobacco

PPR editing factors are believed to be highly specific in that they recognize only one (or at most a few) sites in chloroplasts. By contrast, non-PPR components of the editosome (e.g., MORF/RIP, OZ, and ORRM proteins; reviewed in Sun et al. (2016)) are shared by multiple sites. For instance, two MORF/RIP proteins, MORF2/RIP2 and MORF9/RIP9, are required for complete editing at almost all chloroplast sites in *Arabidopsis* (Takenaka et al. 2012). We, therefore, speculated that the presence of excess quantities of QED1 in tobacco chloroplasts might limit the availability of essential auxiliary factors required for editing at other sites. If this were the case, editing defects would be expected to occur at numerous endogenous sites in tobacco chloroplasts. To test this possibility, editing at all the known tobacco sites was determined using the iPLEX/MassARRAY® technology (see Materials and Methods) in two independent HPL (HPL::At-QED1#8 and #16) and two independent UBQ lines (UBQ::At-QED1#1 and #2) that have up to 12-fold differences in *QED1* expression levels (see sections below). Additionally, pale, variegated, and green material from an individual leaf of each UBQ::At-QED1 line was separately harvested and analyzed. To control for potential editing defects caused by paleness, the complete editotype of three independent pale mutants was also determined (Δ*atpB*, Δ*ycf3*, and WX7; see Materials and Methods for a description of these lines).

The chloroplast editotype of the QED1-expressing transgenic lines largely resembled that of the wild type, regardless of the growth condition. Only in the pale tissue of the QED1-overexpressing UBQ lines, editing at several sites was strongly reduced (e.g., *ndhB*-204, *ndhB*-277, *ndhD*-128, and *ndhD*-200; supplementary fig. S5, Supplementary Material online). However, reductions at these sites resembled those observed in the pale mutants that served as controls. Hence, the editing defects observed at these sites are most likely caused by the pale phenotype (i.e., the photosynthetic deficiency) and not by the overexpression of QED1. Editing at site *ndhB*-204, for example, was previously shown to be severely reduced in pale tissues upon compromised chloroplast translation in tobacco (Karcher and Bock 1998) and in non-photosynthetic mutants (Karcher and Bock 2002). Our data support previous reports that RNA editing is little, if at all, regulated by environmental and growth conditions, but can be strongly affected by the developmental state of the plastid (Bock et al. 1993; Hirose et al. 1996; Chateigner-Boutin and Hanson 2003).

Thus, expression of the foreign *Arabidopsis* PPR QED1 does not significantly alter editing efficiency at any of the endogenous tobacco chloroplast sites, despite the use of the strong *UBQ* promoter that results in an accumulation of *QED1* up to 12-fold higher than in the HPL lines (see below). Consequently, potential editing defects caused by the introduction of QED1 in tobacco can be excluded as the cause of the observed mutant phenotypes.

### *Arabidopsis* QED1 Targets Novel Editing Sites in Tobacco

Given the RNA-binding properties of PPR proteins, another possible explanation for the mutant phenotypes observed in the transgenic lines is that strong expression of *Arabidopsis* QED1 results in binding to and induction of editing in essential tobacco chloroplast transcript(s). Off-target editing by QED1 could cause C-to-U changes that result in nonsense or missense mutations in coding regions. Off-target binding (not necessarily editing any cytidine) could interfere with maturation (e.g., splicing, 5´ or 3´ processing) or translation of the bound RNA molecule(s).

To characterize the organellar transcriptome of the QED1 tobacco mutants, we performed next-generation strand-specific RNA sequencing (RNA-seq) on two HPL::At-QED1 (#1 and #6) lines, two UBQ::At-QED1 (#1 and #3) lines and two wild-type tobacco plants. The analyses were focused on the detection of potential additional sites edited by *Arabidopsis* QED1. Since the QED1-expressing lines were grown under very different conditions (HPL::At-QED1 plants and wild type autotrophically, UBQ lines heterotrophically on sucrose-containing medium or in permissive conditions; see Materials and Methods), a direct quantitative comparison of their transcriptomes is not possible.

We selected stringent criteria for defining SNPs in the obtained RNA-seq libraries (see Materials and Methods) to confidentially identify only authentic off-target editing sites of *Arabidopsis* QED1. Our analysis, therefore, may underestimate the actual off-target editing capacity of *Arabidopsis* QED1. Based on our criteria, we found 31 high-confidence off-target sites edited by *Arabidopsis* QED1 (in addition to *ndhB*-291) in tobacco chloroplasts. Four sites occurred in HPL::At-QED1#6, five in HPL::At-QED1#1, 15 in the “weak” overexpression line UBQ::At-QED1#3 and 26 in the severely variegated UBQ::At-QED1#1 line (table 2). Generally, sites edited in the HPL lines also occurred in both the weak and the strong UBQ line. Similarly, SNPs identified in the weak UBQ::At-QED1#3 line were also found in the severely affected UBQ::At-QED1#1 line. Fourteen sites were exclusively edited in UBQ::At-QED1#1. Editing efficiencies were generally higher in the UBQ overexpression lines compared to the moderate HPL lines. For instance, a site occurring in the intron of *petB* (*petB*_intr-mid) was edited to 40.1% and 48.0% in the HPL::At-QED1 lines (#6 and #1, respectively), and to 86.5% and 87.4% in the UBQ::At-QED1 lines (#3 and #1, respectively). In some cases, editing efficiency was lower in the strong UBQ::At-QED1#1 line compared to the weak UBQ::At-QED1#3 line. However, it needs to be borne in mind that UBQ::At-QED1#1 plants showed the most severe phenotype and needed to be grown in very different conditions.

**Table 2.** *Arabidopsis* QED1 Off-targets in Tobacco Identified by RNA-seq.

	SNP position	Editing %							
		HPL::At-QED1 #6	HPL::At-QED1 #1	UBQ::At-QED1#3 weak	UBQ::At-QED1#1 strong	WT	Site	Location	Impact
**Cp**	97,711	80.3	84.3	88.9	91.8	0	*ndhB*-291	CDS	S->L
	77,612	40.1	48.0	86.5	87.4	0	*petB*_intr-mid	intron	–
	31,530	30.6	31.2	36.3	19.3	0	*trnD-psbM*_intergenic	intergenic	–
	16,335	30.2	38.8	72.2	68.7	0	*rps2*-203	CDS	S->L
	7359	10.4	13.9	0	32.9	0	*trnQ*_3as	intergenic, antisense	–
	128,607	0	5.2	0	0	0	*ycf1*_as	antisense	–
	119,671	0	0	30.0	44.2	0	*ndhE*-96	CDS	S->L
	33,880	0	0	24.6	37.9	0	*psbD*_5UTR	5´ UTR	–
	78,993	0	0	17.6	7.8	0	*petB*-*petD*_intergenic	intergenic	–
	98,961	0	0	16.6	26.6	0	*ndhB*-101	CDS	Q->STOP
	77,495	0	0	16.2	34.0	0	*petB*_intr	intron	–
	124,713	0	0	14.9	14.7	0	*ndhH*-136	CDS	R->STOP
	64,242	0	0	13.7	0	0	*ycf10*-*petA*_intergenic	intergenic	–
	29,881	0	0	10.4	15.0	0	*petN*_3UTR	3´ UTR	–
	12,570	0	0	8.4	20.3	0	*atpF*-65	CDS	R->STOP
	32,653	0	0	7.7	0	0	*trnE*_intergenic-upstream	intergenic	–
	93,937	0	0	6.8	0	0	*ycf2*_as2	antisense	–
	115,955	0	0	5.0	0	0	*rpl32-trnL*_intergenic-as	intergenic, antisense	–
	116,881	0	0	0	26.8	0	*ccsA*-182	CDS	S->L
	129,614	0	0	0	14.9	0	*ycf1*-661	CDS	synonymous
	114,743	0	0	0	14.7	0	*rpl32*_5UTR	5´ UTR	–
	74,956	0	0	0	11.8	0	*psbB*-27	CDS	T->I
	69,550	0	0	0	11.5	0	*psaJ*_5UTR	5´ UTR	–
	83,340	0	0	0	9.7	0	*rpl14*-69	CDS	synonymous
	5800	0	0	0	9.5	0	*rps16*_intr	intron	–
	95,383	0	0	0	6.7	0	*ycf2*-2167	CDS	Q->STOP
	120,048	0	0	0	5.6	0	*ndhG*-*ndhE*_intergenic	intergenic	–
	90,236	0	0	0	5.4	0	*ycf2*-451	CDS	P->L
	90,203	0	0	0	5.4	0	*ycf2*-440	CDS	T->M
	90,244	0	0	0	5.1	0	*ycf2*-454	CDS	L->F
	90,230	0	0	0	5.1	0	*ycf2*_as	antisense	–
	61,515	0	0	0	5.0	0	*accD*_3UTR	3´ UTR	–
**mt**	19,520	0	10.6	34.8	46.7	0	*ccmC*-83	CDS	H->Y
	**QED1 reads**	1.8	2.8	24.3	24.7	0			

Unexpectedly, one off-target site was identified in the tobacco mitochondrial transcriptome. Editing at this site converts a histidine (CAU) into a tyrosine (UAU) codon at position 83 of the *ccmC* gene, which encodes a component of the cytochrome *c* biogenesis system. 10.6% C-to-U conversion at this site was detected in one HPL line (HPL::At-QED1#1; table 2). Similar to what had been observed for the chloroplast sites, the overexpression of QED1 resulted in increased editing efficiency at *ccmC*-83 (34.8% and 46.7% in the weak and strong UBQ::At-QED1 lines, respectively; table 2). The mitochondrial *ccmC*-83 and seven chloroplast QED1 off-targets (*rps2*-203, *psbD*_5UTR, *petN*_3UTR, *ndhE*-96, *ccsA*-182, *petB*_intr, and *petB*_intr-mid) were further validated by bulk sequencing (supplementary fig. S6, Supplementary Material online).

Around 30–40% of the obtained reads did not map to the organellar genomes (supplementary table S6, Supplementary Material online), and mainly represented reads of transcripts encoded in the nuclear genome. These reads could be exploited to estimate the expression of the *QED1* transgene in the analyzed tobacco lines (see Materials and Methods). We found that the expression of *QED1* was between 8- and 13-fold higher in the weak UBQ mutant (UBQ::At-QED1#3) than in the HPL lines (HPL::At-QED1#6 and #1, respectively). Surprisingly, the relative expression of *QED1* in the two overexpression lines (UBQ::At-QED1#3 and #1; table 2) was very similar, despite the two lines showing very different molecular and physiological phenotypes. Because our RNA-seq analysis was optimized for the identification of off-target editing sites in organellar transcripts, we employed quantitative real-time PCR (qRT-PCR; see Materials and Methods) for a more precise quantification of the abundance of nuclear *QED1* transcripts. We found that line HPL::At-QED1#16-4 had the lowest relative expression of *QED1* relative to the housekeeping gene *ACTIN* (supplementary fig. S7 and table S7, Supplementary Material online). This line, therefore, served as reference for calculating the relative expression levels of the *QED1* transgene in the individual transformants. The variation of relative expression of *QED1* among the HPL lines was rather low, with HPL::At-QED1#11-4 having the highest *QED1* expression level (2.7-fold higher than in the reference line HPL::At-QED1#16-4). By contrast, *QED1* expression was 9- and 12-fold higher in the “weak” UBQ::At-QED1#3 and “strong” UBQ::At-QED1#1 overexpression line, respectively (supplementary fig. S7 and table S7, Supplementary Material online). qRT-PCR and RNA-seq analyses confirmed the different expression levels of *QED1*, as expected from the use of the moderate HPL versus the strong UBQ promoter and the different molecular and physiological phenotypes of individual UBQ transgenic lines.

The RNA-seq data revealed that the *Arabidopsis* QED1 protein targets at least 33 sites in tobacco chloroplasts and mitochondria. Importantly, increased expression of QED1 in tobacco resulted in increased editing efficiency and an increased number of target sites. Two distinct sets of QED1 targets in tobacco can be distinguished. The first set includes sites which are edited by QED1 when expressed at moderate levels. This class includes, in addition to *ndhB*-291 and the endogenous *Arabidopsis* sites, targets identified in the RNA-seq libraries of the HPL plants: *petB*_intr-mid, *trnD*-*psbM*_intergenic, *rps2*-203, *trnQ*_3as, and *ycf1*_as in the chloroplast, and *ccmC*-83 in the mitochondria. The second class includes sites that can be edited only upon QED1 overexpression (sites uniquely identified in the UBQ lines). We, therefore, designated these two sets as high- and low-affinity targets, respectively.

### QED1 Editing Targets Share Sequence Similarity and are Rich in A and U Residues

**Fig. 7.**
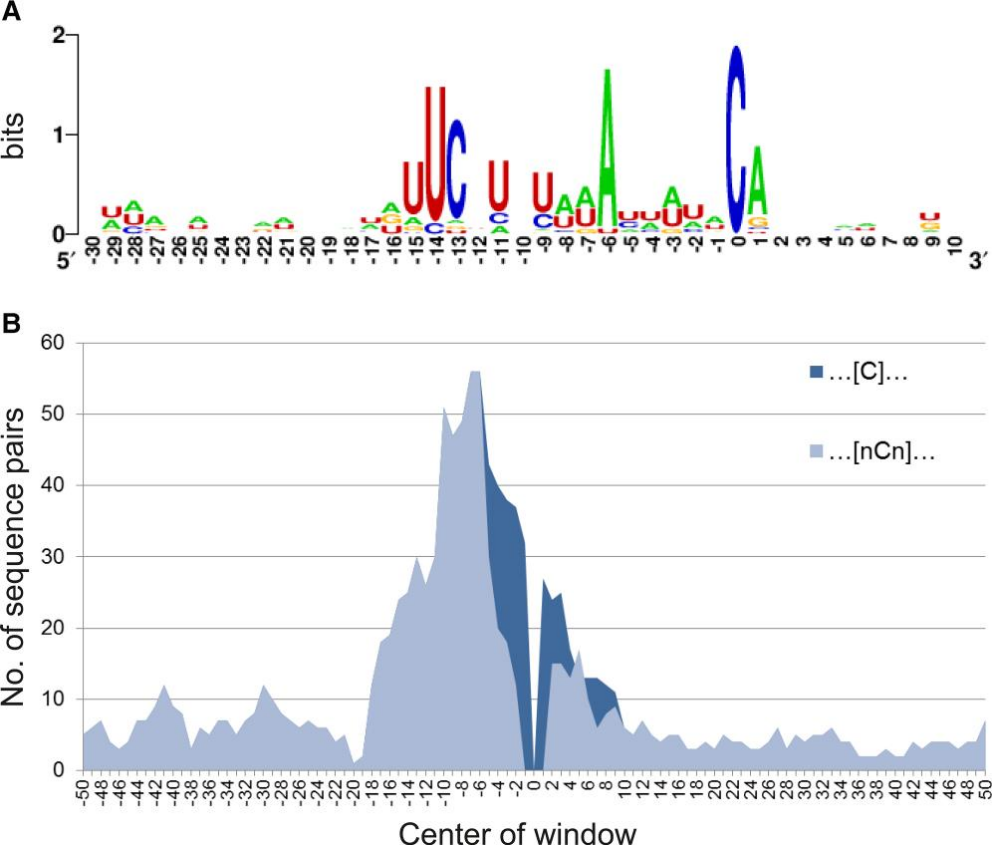
Analysis of the *cis*-elements of QED1 targets. (*A*) Sequence logos created with WebLogo (Crooks et al. 2004) for the regions surrounding the 24 QED1 target sites showing ≥10% C-to-U conversion (five targets in *Arabidopsis* and 19 in tobacco). The sequences from −30 to +10 with respect to the edited C were analyzed. The height of the stack indicates overall sequence conservation at each position. The height of each nucleotide within the stack indicates its relative frequency of occurrence at a particular position. (*B*) Mean pairwise sequence similarity calculated per window of 14 nt for the 23 target sites of *Arabidopsis* QED1 using Lola (Tillich et al. 2006). A 100 nt region surrounding the editing sites (from −50 to +50) was considered. Graphs represent the sequence pair similarities calculated by excluding only the editing site (position 0; [C], dark blue) or the positions from −1 to +1 ([nCn], light blue).

The total number of sites targeted by *Arabidopsis* QED1 rises to 38 if the five endogenous *Arabidopsis* targets (*ndhB*-291, *matK*-214, *rpoB*-811, *accD*_3UTR, and *rps12*_i1) are added to the 33 off-target sites identified in tobacco (table 2). It is intriguing that a single PPR protein is capable of recognizing such a large number of targets in the organelles. To determine whether the identified QED1 targets share particular sequence features, the regions containing the *cis*-elements for editing site recognition (i.e., the nucleotide sequences from position −20 to +5 with respect to the editing site for all 38 sites) were aligned to the *Arabidopsis* QED1 protein based on the current PPR code (table 3). Only those 24 target sites whose editing efficiency exceeded 10% in at least one RNA-seq library were considered for statistical analysis (table 3). Since the sequence surrounding *ndhB*-291 is identical in *Arabidopsis* and tobacco, this target was counted only once. The 24 targets showed an average of five mismatches to the RNA sequence predicted to be recognized by QED1 based on the PPR code (table 3). The *Arabidopsis* site *rps12*_i1 and the tobacco *trnD*-*psbM*_intergenic site are the best-matching targets with only two mismatches each, followed by two of the genuine QED1 targets (*Arabidopsis matK*-214 and *accD*_3UTR) and two tobacco off-targets (*petB*_intr-mid and *ndhH*-136) with three mismatches each. Interestingly, some PPR motifs seem to allow a considerably higher number of mismatches compared with others (e.g., motif 12 (S1), 22/24 mismatches; motif 14 (L2), 21/24 mismatches). Most targets carry a nucleotide at these two positions (−7 corresponding to PPR motif 12 and −5 corresponding to motif 14) that differs from the one predicted by the code. By contrast, some other motifs barely tolerate mismatches such as motif 5 (S1), motif 10 (P1), and motif 13 (P2), where non-predicted nucleobases are very rare (2/24 at −14, 2/24 at −9, 1/24 at −6; table 3). When only the most frequently occurring nucleotides are considered, the binding sites of *Arabidopsis* QED1 are highly AU-rich (AUAUUCUUAUAAAUUAUAC, with the underlined C being the editing site). On the other hand, the overall sequence consensus recognized by *Arabidopsis* QED1 is highly degenerated (NNNNDNYNNHNNNDWNNDNNCNNNNN, from −20 to +5; fig. 7*A*). Nonetheless, in a few positions, specific nucleotides are remarkably overrepresented, in particular at −14, −13, and −6 (fig. 7*A*). These positions correspond to PPR motifs that appear to allow the lowest number of mismatches (table 3). We further calculated the pairwise sequence similarities for the 100 nt-long stretch (from −50 to +50) surrounding the 24 target sites (see Materials and Methods). As expected, the region harboring the putative *cis*-elements for recognition of the target cytidine (from position −18 to +8) shares high sequence similarity ([C]; fig. 7*B*). In particular, the sequences upstream of the editing sites are more similar to each other than the sequences downstream. The highly similar sequence stretch from position −18 to −3 perfectly coincides with the predicted binding site of QED1 (table 3). The removal of positions −1 and +1 from the analysis decreased to some extent the degree of similarity of the immediate surrounding sequences ([nCn]; fig. 7*B*). In fact, *Arabidopsis* QED1 targets mostly occur within an ACA or UCA context (with the underlined C being the editing site; supplementary table S8, Supplementary Material online). A particularly strong bias is observed with respect to position +1. Nineteen out of the 24 analyzed target editing sites are followed by an A (position +1), regardless of the identity of the upstream base. This bias is not caused by the overall AT-richness of the plastome, in that U is significantly underrepresented at this position. Only one of the targets edited to greater than 10% is followed by a U (and is edited at very low efficiency; *petN*_3UTR, 15%; table 3). The bias at position −1 is less pronounced, but overall, A and U are considerably overrepresented. Surprisingly, three off-targets (*ccsA*-182, *petB*-*petD*_intergenic and *ycf10*-*petA*_intergenic) possessed a G at −1, a nucleotide that is generally associated with inefficient editing when present immediately upstream of the editing site (Farre et al. 2001).

**Table 3.** Alignment of *Arabidopsis* QED1 Target Sequences.

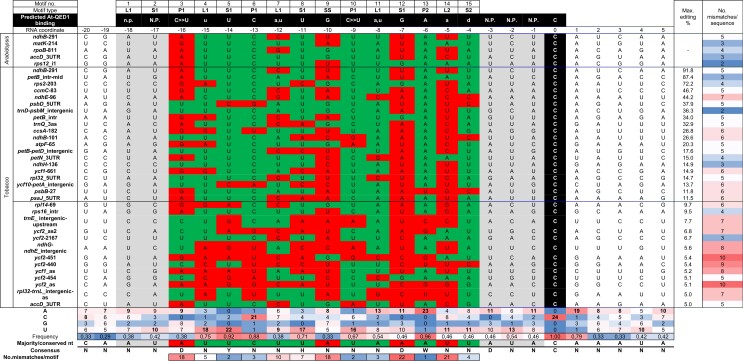																													

Sequences surrounding the five *Arabidopsis* and 33 tobacco QED1 editing sites from position −20 to +5 were extracted and aligned to the predicted QED1 binding site. The last predicted PPR motif (no. 15) is aligned to position −4 from the editing site. Each position is scored for match (green) or mismatch (red) to the predicted nucleotide based on (Barkan et al. 2012) (upper-case) and (Takenaka et al. 2013) (lower-case). Grey: not predicted (n.p. or N.P.). The number of total mismatches occurring in each sequence or at individual positions is indicated. For the tobacco sites, maximum editing conversion detected by RNA-seq (see table 2) is reported, with the black horizontal line indicating the 10% significance threshold applied. Site *ndhB*-291 is considered only once. The occurrence of each base (A, C, G, or U) is calculated for each position of the analyzed sequence interval. Frequencies and number of mismatches are color coded from lowest (blue) to highest (red). Conserved nucleotides among all QED1 targets are reported in the row “Majority/conserved nt”. The nomenclature used for the off-targets is as in table 2.

In conclusion, *Arabidopsis* QED1 edits (to >10%) at least 24 sites in *Arabidopsis* and tobacco and is the editing factor with the by far highest number of targets characterized so far. QED1 target sites share high sequence similarity in their *cis*-elements, which are rich in A and U residues. Alignment of the sequences recognized by QED1 revealed that the PPR code only partially predicts the actually occurring nucleobases. It, therefore, seems possible that particular PPR motifs recognize nucleotides different from the ones assumed by current prediction tools, and that the two amino acid code provides an incomplete description of the recognition mode of PPR proteins.

### *Arabidopsis* QED1 Expression Does not Affect *petB* Splicing, but Hampers *petA* Translation in Tobacco

Measurements of photosynthetic parameters and western blot analyses had revealed a significant reduction in cytochrome *b_6_f* complex content caused by the expression of *Arabidopsis* QED1 from the *HPL* promoter in tobacco (fig. 6). In addition to *ndhB*-291, four chloroplast and one mitochondrial site are edited by QED1 in the HPL lines. Among the two individual plants that were analyzed by RNA-seq, editing at the mitochondrial *ccmC*-83 site was detected only in one of the two, but both plants were deficient in cytochrome *b_6_f* complex. Hence, *ccmC*-83 can be excluded as possible cause of the cytochrome *b_6_f* defect. Among the sites edited by QED1 in the HPL lines, *petB*_intr-mid is the target that could directly affect cytochrome *b_6_f*. The *petB* gene encodes the 25 kDa cytochrome *b_6_*, one of the essential subunits of the cytochrome *b_6_f* complex (Zito et al. 1997; Monde et al. 2000). *petB* is expressed together with *psbB*, *psbT*, *psbH,* and *petD* as a polycistronic transcript of about 6 kb (Sugiura 1992). The *psbB* operon (*psbB*-*psbT*-*psbH*-*petB*-*petD* gene cluster) undergoes intensive processing, including removal of the intron sequences that interrupt the *petB* and *petD* coding sequences, and intercistronic cleavage in at least three different positions (Barkan et al. 1986; Westhoff and Herrmann 1988; Meierhoff et al. 2003; Stoppel et al. 2011; Chevalier et al. 2015). Expression of this pentacistronic operon results in at least 13 different mRNA species detectable by northern blot analysis. A number of protein factors have been identified that are involved in the stability, processing, and translation of specific mRNA species originating from the *psbB* polycistronic transcript (Baker et al. 1998; Stoppel et al. 2011). However, a complete understanding of all processing steps and their interdependence is still lacking.

To investigate the possible effect of the *petB*_intr-mid off-target on splicing of *petB* and/or the accumulation of *petB*-containing transcripts, each gene within the *psbB* operon was probed by RNA gel blot hybridization (supplementary fig. S8, Supplementary Material online). No significant changes in the abundance or pattern of the analyzed transcripts could be detected in the HPL lines compared with the wild type (supplementary fig. S8, Supplementary Material online).

To further confirm the *petB* mRNA maturation is unaffected by the off-target editing in the intron, we calculated the rate of *petB* splicing using our RNA-seq datasets. To this end, the total reads that had passed quality filtering (QF) were specifically mapped to either the spliced (i.e., reads extending over the exon1–exon2 junction) or unspliced *petB* sequence (extending over the exon1–intron or intron–exon2 borders), and normalized to the total number of mapped organellar reads. The rate of splicing was then calculated as ratio of normalized spliced/unspliced mapped *petB* transcripts. Neither the relative accumulation of *petB* transcripts nor the ratio of *petB* splicing significantly differed between the wild type and the HPL lines. In both HPL lines, around 72% of the *petB*-containing reads corresponded to the spliced mRNA. Wild-type tobacco showed a similar ratio of spliced to unspliced *petB* transcripts (of approximately 3:1; supplementary table S9, Supplementary Material online). In conclusion, editing at the *petB*_intr-mid site does not result in any detectable impairment of *petB* splicing or accumulation of *petB*-containing transcripts in the QED1-expressing HPL lines. This finding suggests that none of the newly identified off-target editing sites is directly linked to cytochrome *b_6_f* complex abundance (fig. 6).

**Fig. 8.**
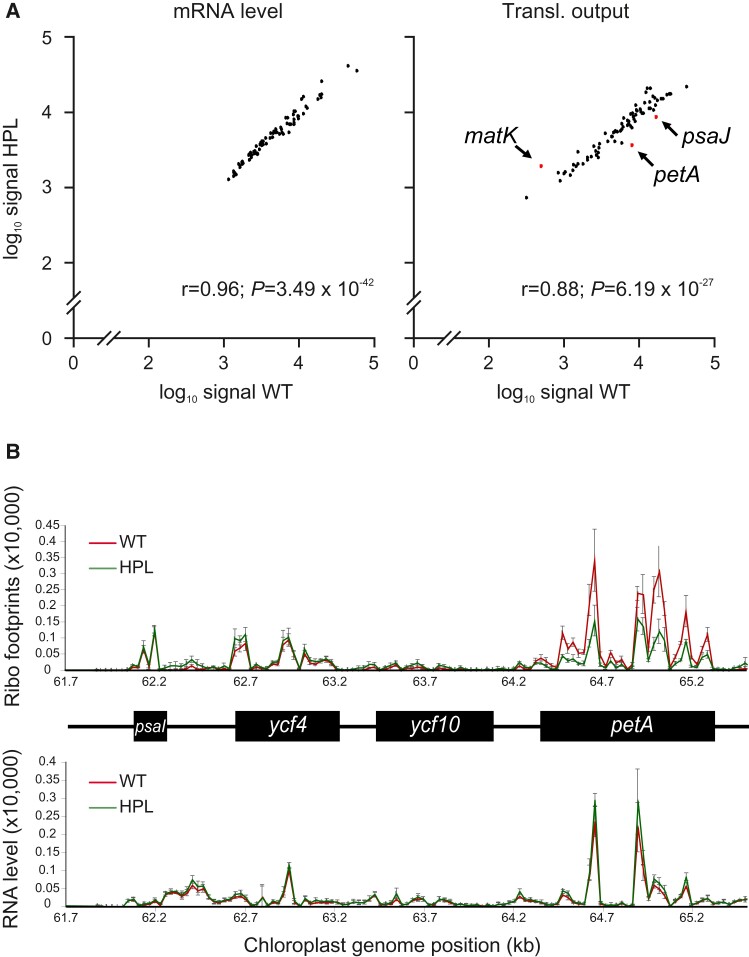
Ribosome profiling of tobacco wild-type and HPL lines. (*A*) Scatter plots of mRNA abundance (left) and translational output (right) of all chloroplast reading frames in wild-type tobacco (WT; *x*-axis) and HPL::At-QED1 mutants (HPL; *y*-axis). Values are plotted in log_10_ scale against each other. Graphs share the same *y*-axis scale. Reading frames whose translational output changes 2-fold or more are highlighted in red. (*B*) Relative ribosome occupancy (top) and RNA accumulation (bottom) within the *psaI*-*ycf4*-*ycf10*-*petA* transcriptional unit in wild-type tobacco (WT; red), and HPL::At-QED1 lines (HPL; green). Error bars represent the standard deviation of three biological replicates. Genomic positions are indicated on the *x*-axis in both graphs according to the database entry of the tobacco chloroplast genome (NC_001879).

Alternatively, it seems possible that, by binding to additional transcripts in tobacco chloroplasts, *Arabidopsis* QED1 inhibits RNA processing or translation, without necessarily alternating the coding sequence by RNA editing. To test this possibility, we analyzed the translational output of all the chloroplast-encoded reading frames by ribosome profiling ((Zoschke et al. 2013; Trösch et al. 2018); Supplementary Dataset S1, Supplementary Material online; see Materials and Methods). In the HPL lines, protein synthesis levels were significantly reduced only for *matK*, *psaJ,* and *petA* (fig. 8*A*). The strongest reduction was detected for *petA*, which encodes the essential cytochrome *f* subunit of the *b_6_f* complex (Schottler et al. 2015). While the abundance of *petA* mRNAs in the HPL lines is virtually identical to the wild type (fig. 8), the *petA* translation rate is reduced about 3-fold, as evidenced by the reduced number of ribosomal footprints covering the *petA* reading frame (fig. 8*B*). The reduced number of footprints along the entire coding region argues against ribosome stalling at specific positions within the *petA* mRNA. Instead, a defect in translation initiation more likely explains the overall reduction in translating ribosomes associated with *petA* in the HPL lines. Interestingly, a QED1 binding site is present in the intergenic region between *ycf10* (also named *cemA*) and *petA*, as evidenced by detection of off-target editing in our RNA-seq analyses (at nucleotide position 64,242 in our customized reference *N. tabacum* plastome; table 2). Editing at this site was only detected in one of the UBQ lines, and barely passed our stringent cut-off applied (13.7% C-to-U conversion; table 2). No other potential QED1 binding sites could be identified in the intergenic *ycf10*-*petA* region, not even by using a highly degenerated consensus (supplementary fig. S9, Supplementary Material online). It, therefore, appears possible that QED1 binds to the *petA* 5' UTR, but does not (efficiently) edit the target cytidine in the HPL lines (possibly due to the presence of unfavorable neighboring nucleotides such as the 3' C; cf. fig. 7*A*).

Although we currently cannot ultimately distinguish between off-target editing and off-target binding (without editing), the pronounced and specific defect in *petA* translation detected by ribosome profiling in the HPL lines strongly suggests that the reduction in cytochrome *b_6_f* complex accumulation detected by our spectroscopic measurements and immunoblots (fig. 6) is likely caused by impaired synthesis of PetA. Cytochrome *f* is an essential component of the *b_6_f* complex and is known to affect stability and assembly of all other subunits (Monde et al. 2000)

### Cacao QED1 Edits a Subset of off-target Sites in Tobacco

In addition to *ndhB*-291, *Arabidopsis* QED1 edited 32 sites in tobacco, as revealed by our RNA-seq analysis. The cacao ortholog was able to partially edit *ndhB*-291 in tobacco only when expressed from the strong UBQ promoter (fig. 4), indicating that the cacao protein may be less competent for *ndhB*-291 editing than the *Arabidopsis* QED1. To further explore possible functional differences between the *Arabidopsis* protein and the cacao protein, we wanted to determine the competence of the cacao QED1 to edit any of the additional sites targeted by the *Arabidopsis* QED1 in tobacco (tables 2 and 3).

Due to the limited sensitivity of Sanger sequencing for detection of low-level editing (∼5–10% detection limit), a customized iPLEX/MassARRAY® assay was designed to assess editing at off-target sites in the transgenic tobacco lines expressing the cacao QED1 (see Materials and Methods). We focused on sites with editing efficiencies ≥10% in at least one RNA-seq library (table 3) and limited this analysis to the UBQ lines, which partially edited *ndhB*-291 (UBQ::Tc-QED1#2, #4, and #5).

A total of seven sites were found to be edited above 5% by cacao QED1 in tobacco chloroplasts (table 4). The three analyzed UBQ::Tc-QED1 transformants showed differences in both editing efficiency and number of targets, with line UBQ::Tc-QED1#5 showing the highest editing activity (six sites edited above 5%). Editing of *ndhB*-291 was also higher in UBQ::Tc-QED1#5 (66%) compared with lines #2 (50%) and #4 (53%), suggesting that the expression level of the cacao *QED1* transgene may be higher in this transgenic lines. In addition to *ndhB*-291, all tested UBQ::Tc-QED1 lines edited *petB*_intr-mid and *rps2*-203, which were defined as high-affinity targets of off-target editing by *Arabidopsis* QED1. Importantly, in all analyzed transformants, editing efficiency at *petB*_intr-mid was higher than at *ndhB*-291. By contrast, in the *Arabidopsis* QED1 lines, *ndhB*-291 was always the site edited at the highest efficiency, independent of the promoter used (table 2). These observations strengthen the hypothesis that the cacao QED1, although possessing some editing capacity for *ndhB*-291 in tobacco, is significantly less active on this site than the *Arabidopsis* protein.

**Table 4.** Additional Sites Targeted by Cacao QED1 in Tobacco as Identified by iPLEX/MassARRAY® Analyses.

		UBQ::Tc-QED1			WT
	Site	#2	#4	#5	
High affinity	***ndhB*-291**	**50.3**	**52.6**	**66.2**	0
	***petB*_intr-mid**	**68.6**	**68**	**83.8**	0
	***rps2*-203**	**26.2**	**27.1**	**48.1**	0
	*trnQ*_3as	0	3.1	0	0
Low affinity	*ndhE*-96	0	0	3.9	0
	*psbD*_5UTR	4.3	2.8	0	0
	*petB*-*petD*_intergenic	0	0	0	0
	***ndhB*-101**	0	**6**	0	0
	***petB*_intr**	**5.5**	**9.9**	**26.2**	0
	*atpF*-65	0	0	0	0
	***ccsA*-182**	0	1.7	**16.1**	0
	*ycf1*-661	0	0	0	0
	*rpl32*_5UTR	0	0	0	0
	***psbB*-27**	0	0	**9.3**	0
Control sites	*ndhA*-358	100	100	100	100
	*ndhB*-50	100	100	100	100
	*ndhD*-225	91.6	97.5	83.9	100
	*rpoB*-184	63.3	61.9	58	53
	*rps16*_intr	100	96.7	100	100

The editing status of QED1 off-target sites in wild-type tobacco (WT) and UBQ::Tc-QED1 transgenic lines was determined using a customized iPLEX/MassARRAY® assay. Values represent editing efficiencies (measured as T/C ratios) in %. Sites are classified in high-affinity and low-affinity targets based on the criteria described in the text. Five endogenous tobacco editing sites are included as control. Sites edited to at least 5% by the cacao QED1 are marked in bold. The nomenclature used for the off-targets is as in table 2.

Site *trnQ*_3as was barely edited by cacao QED1 (table 4). However, this site had been identified only in the RNA-seq library of the strong UBQ::At-QED1#1 line (table 2). Among the low-affinity targets, cacao QED1 edited up to four sites: *ndhB*-101, *petB*_intr, *ccsA*-182, and *psbB*-27. Editing efficiency at these sites largely differed depending on the individual transgenic line (table 4). *petB*_intr was the only low-affinity site edited above 5% in all three analyzed UBQ::Tc-QED1 lines.

The mitochondrial *ccmC*-83 was not included in the MassARRAY® assays and, therefore, was analyzed separately by Sanger sequencing. Surprisingly, similar to *Arabidopsis* QED1, also the cacao protein edited *ccmC*-83, although at significantly lower efficiency and only when expressed from the strong *UBQ* promoter (supplementary fig. S10, Supplementary Material online). Only the most editing-active UBQ::Tc-QED1#5 line, which edits the highest number of chloroplast sites based on the iPLEX/MassARRAY® assay, edited *ccmC*-83 to a level detectable by bulk sequencing of amplified cDNAs.

In conclusion, like *Arabidopsis* QED1, a fraction of the cacao QED1 protein appears to be targeted to mitochondria and is capable of editing the mitochondrial site *ccmC*-83 in tobacco. In addition, cacao QED1 is able to edit at least seven of the 14 off-target sites that the *Arabidopsis* QED1 edits in tobacco plastids.

### Overexpression of the *ndhB*-291 Site Alleviates the QED1 Overexpression Phenotype

Genes encoding PPR-type editing factors are expressed at low to medium levels compared with most other proteins, including non-editing PPRs (Lurin et al. 2004; Loiacono et al. 2019; Fuchs et al. 2020). The overexpression of QED1 in tobacco resulted in the appearance of additional editing sites which are not normally edited by QED1 in species encoding it, or upon heterologous QED1 expression from a promoter of moderate strength (*HPL*). Our RNA-seq analysis revealed a clear dependence of both the number of targets and the editing efficiency on the expression strength of QED1 (table 2). We defined two categories of off-target sites: high-affinity sites, which are edited by QED1 when expressed at a moderate level, and low-affinity targets, whose editing is achievable only upon overexpression of QED1. We hypothesized that the abundance of a high-affinity target may influence the editing activity of QED1 towards other sites, especially low-affinity sites. To test this idea, the same constructs previously transformed into wild-type tobacco plants (HPL::At-QED1 and UBQ::At-QED1) were introduced into a transplastomic line (pRB58; (Bock et al. 1996)) which overexpresses *ndhB*-291. Line pRB58 was previously generated to defining the *cis*-acting sequence determinants for editing of six endogenous sites in the tobacco *ndhB* mRNA. Fragments harboring pairs of editing site pairs were ectopically inserted into the 3´ UTR of the spectinomycin-resistance gene *aadA* (Bock et al. 1996). The chimeric *aadA* construct was expressed from the strong ribosomal operon (P*rrn*) promoter, resulting in overexpression of the ectopic editing sites. For none of the six *ndhB* sites analyzed, the presence of the additional copy did affect the editing efficiency at the endogenous sites in the *ndhB* mRNA. In one of the generated transplastomic lines (pRB58), *ndhB*-291 is included in a fragment that was designed to examine editing at the *ndhB*-279 site.

**Fig. 9.**
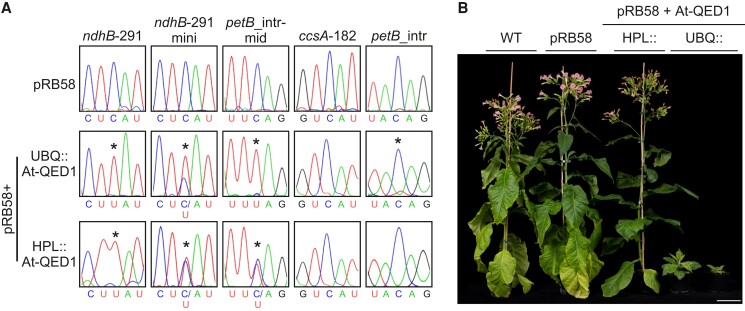
Expression of *Arabidopsis* QED1 in pRB58 transplastomic tobacco plants. (*A*) Editing status of selected QED1 targets in transplastomic pRB58 plants and supertransformed pRB58 lines that express *Arabidopsis* QED1 from the *HPL* promoter (pRB58 + HPL::At-QED1) or the *UBQ* promoter (pRB58 + UBQ::At-QED1). RNA editing was analyzed by Sanger sequencing of the amplified cDNA population. Asterisks indicate sites of C-to-U conversion. (*B*) Phenotype of wild-type tobacco (WT), the transplastomic line pRB58 and the supertransformed plants that express QED1 from the nuclear genome in the pRB58 transplastomic background (pRB58 + HPL::At-QED1 and pRB58 + UBQ::At-QED1) upon autotrophic growth in soil. Sixteen weeks after sowing. Scale bar: 12 cm.

When *Arabidopsis* QED1 is expressed (by nuclear supertransformation) in transplastomic line pRB58 from either the *HPL* or *UBQ* promoter, the *ndhB*-291 site in the *ndhB* mRNA was fully edited (pRB58 + HPL::At-QED1 and pRB58 + UBQ::At-QED1, respectively; fig. 9*A*). The ectopic *ndhB*-291 site residing as a “minigene” in the *aadA* 3' UTR (referred to as *ndhB*-291mini here), however, was only partially edited by QED1 when expressed from the *HPL* promoter (∼50% C-to-U conversion in pRB58 + HPL::At-QED1; fig. 9*A*). Overexpression of QED1 also did not result in full editing at *ndhB*-291mini (pRB58 + UBQ::At-QED1; fig. 9*A*), although it yielded higher editing efficiencies. It is important to note that, in the pRB58 construct, only six nucleotides of the sequence downstream of *ndhB*-291 are present. This sequence is then followed by the 3´ UTR from the tobacco *psbA* mRNA (Bock et al. 1996), which is known to fold into a compact secondary structure that triggers transcript processing and acts as a strong mRNA stabilizing element (Stern and Gruissem 1987; Katz and Danon 2002). It, therefore, seems possible that the proximity of *ndhB*-291 to the 3´ UTR reduces its accessibility to the editosome, thus preventing complete editing of this site.

Surprisingly, all obtained transgenic lines displayed a wild type-like phenotype when grown in aseptic conditions on sugar-containing medium. Notably, the overexpression of *Arabidopsis* QED1 did not result in the variegated phenotype we had observed when the same construct was expressed in the wild type (fig. 5*A*). In standard greenhouse conditions, the HPL lines were indistinguishable from wild-type plants during the entire vegetative state (pRB58 + HPL::At-QED1; fig. 9*B*). More importantly, the UBQ overexpression lines, although significantly retarded in growth, could be grown autotrophically (pRB58 + UBQ::At-QED1; fig. 9*B*). Leaves of greenhouse-grown plants displayed necrotic spots and showed pale-green variegation, although to a lesser extent than the UBQ::At-QED1 plants expressing the transgene in the wild-type background (fig. 9*B* and supplementary fig. S11, Supplementary Material online).

The capability of the overexpression lines in the transplastomic pRB58 background to grow autotrophically suggested that the *Arabidopsis* QED1 may edit only a subset of its targets, when its genuine RNA target *ndhB*-291 is overexpressed. To test this hypothesis, three off-target sites were analyzed by bulk sequencing: the high-affinity *petB*_intr-mid site and the two low-affinity sites *ccsA*-182 and *petB*_intr. Consistent with our previous observations (table 2 and supplementary fig. S6, Supplementary Material online), *Arabidopsis* QED1 partially edited *petB*_intr-mid when expressed from the *HPL* promoter in the pRB58 background, while no editing was detected at *ccsA*-182 and *petB*_intr (pRB58 + HPL::At-QED1; fig. 9*A*). Overexpression of QED1 resulted in complete editing at *petB*_intr-mid in the pRB58 background, similarly to the wild-type background. However, the overexpression of *ndhB*-291 in supertransformed pRB58 plants resulted in a significant decrease in editing efficiency at the low-affinity targets *ccsA*-182 and *petB*_intr in the UBQ lines (pRB58 + UBQ::At-QED1; fig. 9*A*). We then used the iPLEX/MassARRAY® assay (see Materials and Methods) to investigate if overaccumulation of *ndhB*-291 affects also other QED1 targets. In general, the *Arabidopsis* QED1 edited the high-affinity targets in the pRB58 background with similar efficiency as in the wild-type background (table 5). By contrast, the number of low-affinity targets edited by *Arabidopsis* QED1 decreased in the presence of the ectopic *ndhB*-291 site. Only five of the 10 tested low-affinity targets were edited by QED1 overexpressed in the pRB58 background (pRB58 + UBQ::At-QED1; table 5).

**Table 5.** Editing Efficiency of Selected QED1 Targets in the Transplastomic pRB58 Background.

		pRB58 + At-QED1			
		HPL		UBQ	
	Site	#12	#17	#3	pRB58
High affinity	***ndhB*-291**	**88.2**	**84.3**	**87.5**	0
	***petB*_intr-mid**	**54.9**	**49.5**	**70**	0
	***rps2*-203**	**52.8**	**46.1**	**58.2**	0
	***trnQ*_3as**	**14.3**	**24.5**	**13.2**	0
Low affinity	*ndhE*-96	0	0	3.5	0
	***psbD*_5UTR**	3.8	3.9	8	0
	*petB*-*petD*_intergenic	0	0	2.1	0
	***ndhB*-101**	0	0	**10.5**	0
	***petB*_intr**	0	0	**11.3**	0
	*atpF*-65	1.8	1.9	1.8	0
	*ccsA*-182	0	0	3.2	0
	***ycf1*-661**	0	0	**6.1**	0
	***rpl32*_5UTR**	2.4	**5.4**	**7.3**	0
	*psbB*-27	0	0	0	0
Control sites	*ndhA*-358	96.9	87.3	89.1	100
	*ndhB*-50	100	100	97.2	100
	*ndhD*-225	91.8	86.9	82.7	100
	*rpoB*-184	67.6	74	80.1	64.3
	*rps16*_intr	96.8	96.8	97.7	98.3

The editing status of QED1 off-target sites in the pRB58 background was analyzed by iPLEX/MassARRAY® assays. The values given represent the editing efficiency (measured as T/C ratio in %). Sites are classified in high-affinity and low-affinity targets based on the criteria described in the text. Five endogenous tobacco editing sites are included as control. Sites edited to at least 5% are marked in bold. The nomenclature used for the off-targets is as in table 2.

In conclusion, the overexpression of *ndhB*-291 did not affect the capacity of *Arabidopsis* QED1 to edit its high-affinity targets: *ndhB*-291, *petB*_intr-mid, *rps2*-203, and *trnQ*_3as. However, high-level expression of a high-affinity target (*ndhB*-291) reduces or even abolishes editing at most low-affinity off-target sites. This finding strengthens our hypothesis that the phenotype observed upon overexpression of *Arabidopsis* QED1 in tobacco was caused by a molecular defect associated with binding to a low-affinity target in the chloroplast. Moreover, our data suggest that the abundance of high-affinity targets affects the number of editing sites recognized and, in this way, the editing capacity and specificity of PPR-type editing factors.

## Discussion

### Going Against the Flow: Extension of Editing to a Novel Site by Changes in the RNA-binding Domains of a PPR Protein

Chloroplast editing sites are generally lost during evolution of angiosperms by C-to-T mutations in the DNA and, consequently, C-to-U editing is believed to be largely on the way out in seed plant chloroplasts. The emergence of a new editing site by the reverse transition (T-to-C) has been documented in several early-branching land plant clades, including ferns, lycophytes, and hornworts (Small et al. 2020). Our study provides strong evidence of *ndhB*-291 in Brassicaceae being a newly evolved editing site. Interestingly, the emergence of the *ndhB*-291 site in Brassicaceae is not caused by the appearance of a novel PPR editing factor, but rather by changes in the recognition motifs of a pre-existing PPR protein, QED1. The QED1 protein is widespread in all major eudicotyledonous clades, including *Amborella trichopoda*, the most ancient species in our dataset that possesses a *QED1* gene and edits the *matK*-214 and *rpoB*-811 sites (fig. 1 and supplementary table S2, Supplementary Material online). Thus, *matK*-214 and *rpoB*-811 are likely the ancient targets of QED1. The two sites show a typical phylogenetic pattern characterized by multiple losses in distinct angiosperm families (fig. 1*B* and supplementary table S2, Supplementary Material online). By contrast, editing of *ndhB*-291 and the two intergenic sites, *accD*_3UTR and *rps12*_i1, is restricted to the Brassicaceae family (fig. 1*B* and supplementary table S2, Supplementary Material online). Intergenic regions can largely vary among different species and, therefore, it is not possible to unequivocally determine if analogues of *accD*_3UTR and *rps12*_i1 are present and potentially “editable” in non-Brassicaceae species. However, since *rps12*_i1 resides within an unstructured loop of Domain I of the group II intron, it seems unlikely that this site has a strong impact on secondary structure formation of the intron (supplementary fig. S1, Supplementary Material online). By contrast, the sequence surrounding *ndhB*-291, including the predicted binding site of QED1, is strikingly conserved in all the eudicotyledonous species we have analyzed (fig. 2). These observations raise the interesting question whether changes in the QED1 protein facilitated recognition of *ndhB*-291 in Brassicaceae. With few exceptions, structure and motif organization are conserved in the QED1 orthologs (supplementary table S3, Supplementary Material online). Moreover, we could not identify any differences in the binding affinity as predicted by the current PPR code that could explain why QED1 recognizes and edits *ndhB*-291 specifically in Brassicaceae. On the contrary, based on the code, the close QED1 ortholog from *T. cacao*, that does not naturally edit *ndhB*-291, should be more specific to the *ndhB*-291 site than the *Arabidopsis* QED1 (supplementary table S3, Supplementary Material online), but does not edit it. We, therefore, reasoned that the evolutionary changes that allow *Arabidopsis* QED1 to edit *ndhB*-291 are not predictable by the currently known rules of target recognition by PPR proteins. Consequently, we pursued an unbiased strategy based on the construction of chimeric versions of QED1 to determine the minimal requirements for editing of *ndhB*-291. *Theobroma cacao* QED1 was chosen as the closest non-Brassicaceae ortholog that has the identical PPR structure as *Arabidopsis* QED1 and naturally edits *matK*-214 and *rpoB*-811, but not *ndhB*-291 (fig. 1*B*). By introducing specific PPR motifs from the *Arabidopsis* protein, we successfully reprogrammed the cacao QED1 to target *ndhB*-291 in *Arabidopsis* and complement the editing defects of the *qed1-2* mutant (table 1 and supplementary table S4, Supplementary Material online). The large set of chimeric constructs tested by stable nuclear transformation indicate that PPR motifs 9, 10, and 11 (SS-P1-L1) are involved in recognition of *ndhB*-291 by *Arabidopsis* QED1 (table 1 and supplementary table S4, Supplementary Material online). Unfortunately, the QED1 variants could not be successfully purified for systematic biochemical assays that would allow precise quantification of protein-RNA interactions. Nonetheless, our genetic data show that a minimal set of three PPR motifs (SS-P1-L1, motifs 9–10–11) is sufficient to confer editing activity for *Arabidopsis ndhB*-291 to the cacao QED1 protein *in vivo*. Thus, our work has uncovered changes in the recognition motifs of PPR-type editing factors as an evolutionary mechanism that facilitates the gain of new RNA editing sites.

### Expression of a Heterologous PPR Editing Factor Affects Plant Growth by Targeting Essential Chloroplast Transcripts

*Arabidopsis* QED1 efficiently edits the *ndhB*-291 site in tobacco chloroplasts (fig. 4). As previously shown for the PPR protein LPA66 (Loiacono et al. 2019), the strength of the promoter used did not affect editing efficiency at the heterologous site, in that expression of *Arabidopsis* QED1 from the moderate *HPL* promoter is already sufficient to trigger full editing of *ndhB*-291 (fig. 4). However, the obtained transgenic plant lines display a range of mutant phenotypes indicating that the expression of *Arabidopsis* QED1 in tobacco affects chloroplast functionality in a dose-dependent manner. Although a few HPL lines showed a certain degree of leaf variegation, the vast majority of the lines appeared wild type-like and grew photoautotrophically in soil under standard environmental conditions (fig. 5). Detailed analysis of the photosynthetic apparatus revealed that the HPL::At-QED1 lines contain only between 50% and 75% of functional cytochrome *b_6_f* complex, depending on the individual transformant analyzed (fig. 6).

Phenotypic consequences are much more obvious when the *Arabidopsis* QED1 is expressed from the strong *UBQ* promoter. The UBQ lines display a pronounced leaf variegation phenotype and are incapable of growing autotrophically in standard greenhouse conditions (fig. 4). RNA-seq analysis revealed that *Arabidopsis* QED1 targets additional cytidines in the organellar transcriptomes of tobacco (table 2). Remarkably, the number of targets and their editing efficiency correlated with the expression of the *QED1 trans*-gene. A maximum of six off-target sites are edited in the HPL lines (table 2). In the UBQ lines, the expression of *Arabidopsis QED1* is between 9- and 13-fold higher than in the HPL plants (as measured by qRT-PCR; supplementary fig. S7, Supplementary Material online). As a result of the increase in *QED1* expression, more off-targets and higher editing efficiency was observed in the UBQ transgenic lines (table 2). It should be noted that individual plants differ in the editing efficiency of some off-target sites. However, it needs to be borne in mind that our RNA-seq analyses were designed to discover high-confidence off-target sites. The stringent criteria we applied for SNP discovery (see Materials and Methods) eliminate all editing sites with a conversion efficiency of less than 5%. Furthermore, libraries from UBQ::At-QED1#1, the most affected overexpression line, were sequenced with 2-fold depth to ensure the discovery of the maximum number of QED1 off-targets (see Materials and Methods). Therefore, the detection of numerous low efficient off-targets (close to our 5% cut-off) in UBQ::At-QED1#1 but not in UBQ::At-QED1#3 can likely be explained by differences in sequencing depth of the RNA libraries.

Unexpectedly, we discovered one off-target site edited by QED1 in a mitochondrial transcript. Editing at *ccmC*-83 was detected in one HPL line (HPL::At-QED1#1) and both UBQ lines analyzed (table 2 and supplementary fig. S10, Supplementary Material online). QED1 is not expected to be imported into mitochondria based on several localization prediction tools (supplementary table S10, Supplementary Material online). However, high levels of the QED1 protein in these lines could cause some mistargeting to mitochondria. Recent studies have revealed that dual targeting of proteins to mitochondria and chloroplasts occurs more frequently than previously thought (Duchene and Giege 2012; Carrie and Small 2013; Sharma et al. 2018a, b), and that many transit peptides are “ambiguous” in that they cannot be accurately distinguished by the import machineries of the two organelles. As previously observed for the transit peptide of the plastid-localized small subunit of RuBisCO (Tabatabaei et al. 2019), the QED1 transit peptide appears to cause mistargeting of transgenic proteins at low levels to mitochondria. Recently, the first dually localized PPR editing factor was characterized in *Arabidopsis* (AEF1; (Yap et al. 2015; Hein et al. 2020)), suggesting that editing factors can be functional in both organelles.

In its native context, QED1 edits five sites in *Arabidopsis* (Wagoner et al. 2015). When the newly identified tobacco off-target sites are considered, *Arabidopsis* QED1 can edit a total of 37 unique sites in the organelles. The binding consensus of *Arabidopsis* QED1 is highly degenerated and is comprised nearly exclusively of As and Us, when only the most frequently occurring nucleotides are considered (AUAUUCUUAUAAAUUAUAC, editing site underlined; table 2 and fig. 7). Since plastid genomes are highly AT-rich (Kusumi and Tachida 2005; Smith 2012), the bias towards the recognition of A and U may explain why QED1 has more targets than a typical chloroplast editing factor.

Additional non-PPR factors are known to be required for editing of multiple sites (e.g., MORF/RIP, OZ, and ORRM proteins; reviewed in Sun et al. (2016)), and, consequently, they are present in the organelles at higher abundance than PPR editing factors (Fuchs et al. 2020). Their availability seems not to be limiting in the editing of up to 27 sites by *Arabidopsis* QED1 in tobacco chloroplasts. This is evidenced by our finding that only small changes in editing efficiency at the endogenous tobacco chloroplast editing sites are detectable in UBQ::At-QED1#1, the overexpression line with the highest number of QED1 off-targets (supplementary fig. S5, Supplementary Material online). Based on our editotype data, we could also exclude the possibility that off-target editing by the *Arabidopsis* QED1 is primarily responsible for the observed mutant phenotypes. Instead, we identified a potential off-target binding effect that likely causes the defect in cytochrome *b_6_f* complex accumulation in the HPL lines. Ribosome profiling revealed a pronounced and specific reduction in translational efficiency of the *petA* mRNA (fig. 8) that encodes the essential cytochrome *f* subunit of the *b_6_f* complex (Monde et al. 2000). Defects in cytochrome *f* are known to affect assembly of the complex and, in this way, the stability of all other subunits (Monde et al. 2000), thus providing a straightforward explanation for the reduced amounts of functional cytochrome *b_6_f* complex in the transgenic plants (fig. 6). A putative QED1 binding site is present in the intergenic region between *ycf10* and *petA* (supplementary fig. S9, Supplementary Material online). We propose that *Arabidopsis* QED1 binds to the *ycf10*-*petA* intergenic region and prevents efficient translation initiation of the *petA* mRNA. Consistent with this hypothesis, the pattern of ribosome footprints mapped along the entire *petA* reading frame argues against ribosome stalling at a specific position and rather supports a defect in translation initiation (fig. 8). Binding of a DYW-PPR protein independent of editing activity was also reported for the chloroplast PPR protein CRR2 (Hashimoto et al. 2003; Ruwe et al. 2019).

The identification of the molecular basis of the growth defects and leaf variegation phenotype in the UBQ lines is more challenging. Overexpression of *Arabidopsis* QED1 results in up to 32 different off-target editing sites (table 2). It seems reasonable to assume that sites exclusively edited in the overexpression lines but not in the HPL plants are responsible for the strong mutant phenotype observed in UBQ::At-QED1. Also, additive effects of multiple off-targets cannot be excluded. As uncovered by analysis of our HPL lines, off-target binding and not only off-target editing can be deleterious, for example, by interfering with RNA processing, stability and/or translation. It is, however, possible to exclude the mitochondrial off-target *ccmC*-83 as a potential cause of the growth phenotype, because *ccmC*-83 is edited with similar efficiency (table 2) in the “weak” UBQ::At-QED1#3 line grown in soil in permissive conditions and the “strong” UBQ::At-QED1#2 line that can only grow heterotrophically (fig. 5).

It is intriguing that *Arabidopsis* can tolerate a rather promiscuous editing factor like QED1. From our results, it seems reasonable to assume that its low endogenous expression levels prevent QED1 from editing low-affinity targets (see below). The *Arabidopsis* QED1 represents an intriguing example of the tight co-evolution of the nucleus-encoded RNA editing factors and the organellar genome they act upon. It shows that the introduction of a foreign PPR editing factor can result in novel editing events that cause deleterious phenotypes. Recent reports have uncovered extensive off-target effects of native and synthetic PPR editing factors expressed *in planta* (Royan et al. 2021), in *E. coli* (Oldenkott et al. 2019; Bernath-Levin et al. 2021) and in human cells (Ichinose et al. 2022; Lesch et al. 2022). Our analyses of the QED1 protein demonstrate that a PPR editing factor can also edit numerous off-target sites *in planta*. Binding a highly AU-rich sequence (fig. 7), *Arabidopsis* QED1 is certainly unique in that it can target more sites than a typical chloroplast editing factor.

These results highlight the importance of the tight evolutionary co-adaptation of the nucleus and the DNA-containing organelles (Greiner and Bock 2013) and show that introduction of a foreign PPR protein can be sufficient to uncouple the finely balanced cooperation of the genetic compartments.

### The Editing Tie-breaker: Balance of PPR Expression and Abundance of Substrate RNA Molecules

Before the discovery of PPR proteins, the editing activity at a specific site was hypothesized to be dependent on the availability of at that time unknown proteinaceous *trans*-acting factors as well as the possible presence of competing similar target sites (Bock et al. 1996; Heller et al. 2008). Our data reported here ultimately demonstrate that the expression level of a PPR editing factor is in fact a crucial determinant of both the number of sites recognized and the editing efficiency. We demonstrated this by exploiting a previously generated transplastomic tobacco line, pRB58 (Bock et al. 1996), that ectopically overexpresses *ndhB*-291. When *Arabidopsis* QED1 was expressed (by nuclear supertransformation) in the pRB58 line, not only editing efficiency but also the number of sites targeted by QED1 was significantly reduced (fig. 9*A* and table 5). Interestingly, only the low-affinity off-targets were affected by the overexpression of the *ndhB*-291 site. The high-affinity sites (endogenous *ndhB*-291, *petB*_intr-mid, *rps2*-203, and *trnQ*_3as) were edited to a similar extent by *Arabidopsis* QED1 in the wild type and the pRB58 background. Consequently, high-affinity targets can be defined as sites that (i) are already recognized when QED1 is expressed at moderate levels, and (ii) are largely insensitive to the excessive presence of substrate RNAs. In the pRB58 background, the *Arabidopsis* QED1 appears to preferentially bind to the favored *ndhB*-291 site and the other high-affinity targets, but becomes limiting for editing at the low-affinity sites.

In conclusion, the expression of QED1 in tobacco confirmed the long-standing hypothesis that the expression level of PPR-type *trans*-acting editing factors is a crucial determinant of editing efficiency and the number of targets recognized, as proposed in earlier studies (Bock et al. 1996; Heller et al. 2008) and suggested by the recent reconstitution of editing in *E. coli* (Oldenkott et al. 2019). Conversely, overaccumulation of a high-affinity target limits the editing capacity of the PPR protein for low-affinity sites. Taken our data together, we propose that there is strong evolutionary pressure to maintain the fine balance of editing factor abundance, number of targets and editing efficiency.

## Materials and Methods

### Plant Material and Growth Conditions

Plant material used in this study is listed in supplementary table S11, Supplementary Material online. *Nicotiana tabacum* seeds were surface sterilized by treatment with 70% (v/v) ethanol for 7 min, followed by 7 min in 7% (v/v) hypochlorite. Sterilization of *Arabidopsis thaliana* seeds was carried out in 70% (v/v) ethanol and a drop of Tween®20 for 3 min and in 7% (v/v) hypochlorite for 15 min. Seeds were washed five times with sterile water before plating on Murashige and Skoog (MS) medium containing appropriate antibiotics (listed in supplementary table S11, Supplementary Material online for each line used). Half-strength MS medium supplemented with 1% (w/v) sucrose was used for the germination of *Arabidopsis* seeds. Tobacco MS medium was supplemented with 3% (w/v) sucrose, except for growth of plants used for photosynthetic measurements. Antibiotic concentrations used were: 50 mg/L kanamycin monosulphate (Duchefa), 10 mg/L glufosinate ammonium (PPT; Duchefa), 500 mg/L spectinomycin dihydrochloride pentahydrate (Duchefa). To synchronize germination, plated seeds were incubated at 4°C in the dark for two days and then transferred to controlled environment chambers (light intensity: 50 μmol photons/(m^2^ s), diurnal cycle: 16 h light at 24°C and 8 h dark at 22°C). *Arabidopsis thaliana* seedlings were transferred to soil 10–15 days after germination and grown under long-day conditions to maturity: 16 h light at 21°C and 8 h darkness at 19°C. 10–15 days old *N. tabacum* seedlings were transferred to Magenta boxes containing MS medium, 3% (w/v) sucrose and appropriate antibiotics, and grown in aseptic conditions until transferred to soil. Unless otherwise mentioned, tobacco plants were grown in standard greenhouse conditions: 16 h light at 25°C and 8 h darkness at 20°C. Plants used for photosynthetic measurements were grown in controlled environment chambers (York) at 120 μmol photons/(m^2^ s) light intensity (16 h light at 22°C, 75% relative humidity, and 8 h dark at 18°C, 70% relative humidity). After ∼3 weeks, plants were transferred to controlled environmental chambers with the actinic light intensity set to 350 *μ*E/m^2^ s^−1^ (Conviron). All other environmental parameters remained unaltered. Line UBQ::At-QED1#3 grew autotrophically only in permissive conditions (shaded from light, natural photoperiod, 20°C day temperature/18°C night temperature, 60% day humidity/50% night humidity). Pale non-photosynthetic mutants (Δ*atpB*, Δ*ycf3,* and WX7; see supplementary table S11, Supplementary Material online for a detailed description of these lines) were grown in aseptic conditions (MS medium, 3% sucrose) under very low light intensity (<10 μmol photons/(m^2^ s)).

### Cloning and Plant Transformation

PCR products were amplified using appropriate oligonucleotides (listed in supplementary table S12, Supplementary Material online) containing restriction sites or sequences overlapping the target vector at their 5´ and/or 3´ ends. For cloning, PCR products were ligated to the linearized vector of interest (digested with the appropriate restriction endonuclease) by T4 ligase (Promega) or Gibson Assembly® (New England Biolabs) following the manufacturer's recommendations. The cloning strategy for the generation of all vectors used in this work is described in supplementary table S11, Supplementary Material online. Stable nuclear transformation of *N. tabacum* was performed according to (Rosahl et al. 1987) using *A. tumefaciens* strain pGV2260. The floral dip method (Clough and Bent 1998) was applied to introduce chimeric QED1 constructs into the *Arabidopsis qed1-2* mutant using *A. tumefaciens* strain pGV3101. Transgenic tobacco lines were regenerated on RMOP medium (Svab et al. 1990) supplemented with appropriate antibiotics for selection (listed in supplementary table S11, Supplementary Material online). For selection and regeneration of nuclear transformants, the media were supplemented with 250 mg/L cefotaxime sodium salt (claforan) to prevent growth of bacteria. Transgenic *Arabidopsis* seeds were selected on half-strength MS medium supplemented with 1% (w/v) sucrose, 10 mg/L glufosinate ammonium (PPT; Duchefa), and 250 mg/L cefotaxime sodium salt (claforan; Duchefa) to prevent bacterial growth.

### Immunoblot Analysis and Spectroscopic Methods

Immunoblot analysis and spectroscopic measurements were conducted as described in Loiacono et al. (2019). For measurements and harvest of leaf material, plants were used that did not show any leaf variegation. All primary antibodies used were purchased from Agrisera.

### Ribosome Profiling

Ribosome profiling was performed and data were analyzed as described in Trösch et al. (2018). Leaf material used for ribosome profiling was harvested from three wild-type tobacco plants and three independent HPL lines (transformed with the HPL::At-QED1 construct) prior to photosynthetic measurements.

### Northern Blot Analysis

Total plant RNA was extracted using TRIzol® (Thermo Fisher Scientific) following the manufacturer´s protocol and quantified by optical density measurements using a NanoDrop 1,000 spectrophotometer (VWR International). RNA was size separated using an agarose gel electrophoresis system (neoLab). Denaturing 1% (w/v) agarose gels were prepared in 1× MOPS buffer [0.1 M 3-(*N*-morpholino) propanesulfonic acid, 0.3 M NaAc, 1 mM EDTA] in the presence of 16% (v/v) formaldehyde (Sigma). Samples of 5 μg cellular RNA (in 5 µl volume) and appropriate RNA size markers (Thermo Fisher Scientific) were denatured in 24 μl RNase-free RNA sample buffer [1× MOPS buffer (pH 7.0), 25% (v/v) formaldehyde, 1% (v/v) formamide] at 75°C for 15 min and quickly chilled on ice. Denatured RNA was mixed with 3 μl RNase-free RNA loading buffer [50% (w/v) glycerol, 10 mM EDTA (pH 8.0), 0.02% (w/v) bromophenol blue, 0.02% (w/v) xylene cyanol] and separated in denaturing gels in 1× MOPS buffer containing 10–15% (v/v) formaldehyde (Merck KGaA) as running buffer. The run was performed on ice and with continuous mixing of the running buffer by a magnetic stirrer. Separated RNAs were transferred onto Hybond-N nylon membranes (GE Healthcare) by overnight capillary transfer using 5× SSC buffer [0.75 M NaCl, 75 mM tri-sodium citrate dehydrate; final pH 7.0], and covalently cross-linked by UV light (0.120 J/cm^2^) using a BLX-254 UV-crosslinker (Vilber Lourmat). Nylon membranes were stained with 100 ml methylene blue staining solution [0.3 M NaOAc, 0.03% (w/v) methylene blue], washed in sterile water and scanned using EPSON Perfection V700 Photo. Radioactively labelled RNA probes were synthetized with the MAXIscript® Kit (Thermo Fisher Scientific) following the manufacturer's protocol and using [α-^32^P]-UTP (Hartmann Analytic GmbH). For hybridization, nylon membranes were pre-incubated in 25 ml Church buffer [1% (w/v) BSA, 0.5 M Na_2_HPO_4_ (pH 7.2), 7% (w/v) SDS, 1 mM EDTA (pH 8.0)] at 65°C for at least 1 h, and then hybridized to single-stranded RNA probes at 65°C for at least 4 h. Membranes were washed by pre-warmed Wash buffer I [1 × SSC buffer, 0.2% (w/v) SDS] for 5 min, Wash buffer II [0.5 × SSC buffer, 0.2% (w/v) SDS] for 20 min and Wash buffer III [0.2 × SSC buffer, 0.2% (w/v) SDS] for 20 min. Membranes were exposed to a storage phosphor screen (GE Healthcare) for at least 1 h, followed by radioactive signal detection using the Typhoon™ TRIO + scanner (GE Healthcare).

### Bulk Sequencing

One microgram of TRIzol®-extracted RNA was used for complementary DNA (cDNA) synthesis (QuantiTect Reverse Transcription Kit; Qiagen) following the manufacturer's instructions. Reactions were carried out using a 1:1 mixture of random hexamer (Qiagen) and oligo(dT)_18_ primers. cDNA fragments were amplified by PCR using specific oligonucleotides as primers (listed in supplementary table S12, Supplementary Material online) and following standard protocols (*Taq* DNA polymerase, ThermoFisher). PCR products were purified with the NucleoBond PCR Clean-up kit (Macherey-Nagel) and sequenced (Eurofins Genomics).

### qRT-PCR

Total RNA was extracted with the NucleoSpin® RNA plant kit (Macherey-Nagel) according to the manufacturer's protocol, including the recommended in-column DNase digestion step. 1 μg of RNA was reverse-transcribed using the QuantiTect Reverse Transcription Kit (QIAGEN) following the manufacturer´s instructions, including a second DNase digestion step to eliminate any traces of carryover DNA. The reaction was carried out using a 1:1 mixture of random hexamer (Qiagen) and oligo(dT)_18_ primers. Obtained cDNA was diluted 1:20 with nuclease-free water and analyzed in technical triplicates using the LightCycler 480 Real-Time PCR System and LightCycler SYBR Green (Roche Applied Science). Primers used are listed in supplementary table S12, Supplementary Material online. Because the target gene (*Arabidopsis QED1* or cacao *QED1*) is only present in the transgenic lines and absent from wild-type plants, relative expression values are reported (supplementary fig. S7, Supplementary Material online) as ΔΔCt fold changes relative to the transgenic line with the highest ΔCt value, hence, lowest expression (HPL::At-QED1#16-4 for *Arabidopsis* QED1 and HPL::Tc-QED1#6 for cacao QED1). ΔCt values are calculated using *ACTIN* as reference gene (Schmidt and Delaney 2010). Raw data and calculations are reported in supplementary table S7, Supplementary Material online.

### Analysis of RNA Editing by iPLEX/MassARRAY®

Total RNA was extracted with the NucleoSpin® RNA plant kit (Macherey-Nagel) according to the manufacturer's protocol including the recommended in-column DNase digestion step. 1 μg of RNA was reverse-transcribed using the QuantiTect Reverse Transcription Kit (QIAGEN) following the manufacturer´s instructions. The reaction was carried out using a mixture of gene-specific primers in equimolar concentration (0.2 μM each; oligonucleotides used for the priming of cDNA synthesis for each of the assays are listed in supplementary table S12, Supplementary Material online). Obtained cDNA was diluted in nuclease-free water 1:20 and analyzed by iPLEX/MassARRAY® (Agena) following the manufacturer´s instructions. Primers used in each step (amplification and extension reactions) of the assays designed in this work are listed in supplementary table S12, Supplementary Material online. The assays were set up based on the Assay Design Suite Software (Agena). For characterization of the complete tobacco chloroplast editotype, the 48 known tobacco editing sites were analyzed strand-specifically and redundantly. Eighty nine different reactions were designed to cover differently matured RNA species (e.g., spliced versus unspliced transcripts). Six heterologous unedited sites (including *ndhB*-291) were included as control (“Tobacco-editotype”; supplementary table S13, Supplementary Material online). In addition to the QED1-expressing tobacco lines, the complete chloroplast editotype was determined for wild type *N. tabacum* (*n* = 5) and three independent pale mutants generated previously (Δ*atpB* (Karcher and Bock 2002), Δ*ycf3* (Ruf et al. 1997) and WX7; see supplementary table S11, Supplementary Material online for a detailed description of these lines). A second assay was designed to specifically quantify QED1 off-targets in tobacco. It included 13 off-targets, five endogenous tobacco sites as control and *ndhB*-291 (“Tobacco-OFF-targets”; supplementary table S13, Supplementary Material online). For the pRB58 lines, an additional assay was designed to specifically detect editing at the ectopic *ndhB* sites (see Results and Bock et al. (1996) for details). This assay included three endogenous tobacco sites as control (“ndhB-291mini”; supplementary table S13, Supplementary Material online). The *Arabidopsis* assay included the five targets of QED1 and one control site (“Arabidopsis-QED1-targets”; supplementary table S13, Supplementary Material online).

### RNA Sequencing and SNP Discovery

For next-generation RNA sequencing (RNA-seq), leaf material was harvested from HPL::At-QED1 lines and wild-type tobacco plants prior to photosynthetic measurements. As the overexpression line UBQ::At-QED1#1 was severely variegated and retarded in growth (see Results), small green emerging leaves were harvested from several individuals (propagated vegetatively on sucrose-containing medium) and pooled for RNA extraction. Young leaves were harvested from line UBQ::At-QED1#3 grown in the greenhouse in permissive growth conditions (see above). Total RNA was extracted using the NucleoSpin® RNA plant kit (Macherey-Nagel) and depleted of contaminating DNA by two successive DNase I treatments (Macherey-Nagel). rRNA was removed with the Ribo-Zero™ Plant Leaf kit (Illumina). No poly(A) transcript enrichment was performed. Strand-specific paired-end libraries with an average insert size of 250 bp were generated, sequenced (2 × 150 nt) on an Illumina HiSeq3000, and the adapter sequences were trimmed by the Max Planck Genome Centre (Cologne, Germany). For lines HPL::At-QED1#1 and #6 and UBQ::At-QED1#3, a minimum of 8 Gb total reads was requested. The double amount of reads was requested for UBQ::At-QED1#1 (the most affected overexpression line; see Results) to ensure the discovery of the maximum number of QED1 off-targets in tobacco. The number of raw reads obtained from each strand and library is shown in supplementary table S6, Supplementary Material online. RNA-seq data have been deposited as fastq files representing the raw RNA sequencing data with the National Center for Biotechnology Information (NCBI) under BioProject accession number PRJNA629102. After trimming of the adapter sequences, reads were subjected to stringent quality filtering (QF). First, reads were quality filtered using FASTX (http://hannonlab.cshl.edu/fastx_toolkit/index.html). On average, 94% of the reads from each library passed quality filtering. In order to select organellar transcripts, reads were aligned by BLAT (Kent 2002) to customized tobacco plastid and mitochondrial reference sequences based on NC_001879 and NC_006581, respectively. BLAT parameters (tileSize = 10, minMatch = 3, maxIntron = 30,000) were chosen to accommodate also heavily edited reads and the *trans*-spliced *rps12* intron. In the case of the plastome, the customized reference was generated by removing the second copy of the IR except for 75 nt from the 5´ and 75 nt from the 3´ border. For both reference sequences, known editing sites (Sasaki et al. 2003; Grimes et al. 2014) were denoted as Y (C or T) or R residue (A or G) depending on encoding on the + or − strands, in order to avoid a bias in favor of or against editing. Selection of organellar read pairs was performed by a custom Python script. A read pair was selected if both reads were longer than 100 nt and at least one of them could be aligned to one of the references with at least 85% identity and greater than 50% of the read length. Data quality of organellar and non-organellar reads of all libraries was confirmed by FastQC (http://www.bioinformatics.babraham.ac.uk/projects/fastqc/). In order to detect editing sites, all selected reads from all transgenic QED1-expressing lines (HPL::At-QED1#1 and #6, and UBQ::At-QED1#1 and #3) were collapsed using FASTX and mapped to the organellar reference sequences using SeqMan NGen (DNASTAR®), followed by determination of single-nucleotide polymorphisms (SNPs). Reads obtained from the two wild-type samples were also aligned and SNPs determined in the same way. For SNP quantification, organellar reads from the six data sets were mapped and evaluated separately. Only SNPs with a rate ≥5% were considered. For the discovery of potential novel editing sites, SNPs were additionally filtered for the C-to-T or G-to-A type. SNPs occurring at known editing sites or in tRNAs or rRNAs were excluded. tRNAs and rRNAs are hypermodified by enzymes unrelated to mRNA editing enzymes, and unequivocally defining putative off-targets edited by QED1 in those RNA species is challenging. Editing conversion was calculated as T/C or A/G ratios at the SNP position. Lastly, putative off-targets of *Arabidopsis* QED1 in tobacco were defined as SNPs which occurred in at least one assembly of the QED1 mutants (but not in the wild type) with at least 5% editing efficiency. The maximum coverage for each identified SNP is reported in supplementary table S14, Supplementary Material online. QF reads that did not map to the organellar genomes were used to quantify the expression of the *Arabidopsis* QED1 transgene. Reads from each of the reverse (R1) libraries were mapped to the coding sequence of *Arabidopsis* QED1 (AT2G29760) and normalized to the total number of QF reads using the RPKM method (Mortazavi et al. 2008). The rate of *petB* splicing was calculated by mapping reads that had passed the quality control to either the spliced (covering the exon1–exon2 junction) or unspliced *petB* sequence (covering the exon1–intron or intron–exon2 junction) and normalized to the total number of mapped organellar reads (Mortazavi et al. 2008). The rate of splicing was calculated as ratio of normalized spliced to unspliced mapped *petB* transcripts.

### Phylogenetic and Bioinformatic Analyses

Chloroplast genomic sequences of the genes *matK* (3,822 sequence entries from 3,793 species), *rpoB* (3,782 entries, 3,770 species), and *ndhB* (7,183 entries, 3,686 species) were obtained from the CpGDB-database (http://www.gndu.ac.in/CpGDB/; (Singh et al. 2020)). For the three editing sites, the presence of genomic C or T alleles in the respective genomes were identified as exact blastn matches of the respective C/T-site +/15 nt, with *matK*-214: TTCTTATATAATTCT**C/T**ATGTATGTGAATACG, *rpoB*-811: TTCGTGTATATATTT**C/T**ACAGAAACGTGAAAT, *ndhB*-291: TTCCTTTTTATTTCT**C/T**ATCAAATGAATGGCA against the respective CpGDB gene sets. The blastn settings were: evalue = 1e-3, wordsize = 5, max_target_seqs = 500,000; otherwise, default parameters were used. Associated alleles in transcripts were identified using the same 31-mers as used in the genome searches and also filtering for exact matches in blastn outputs (parameters as given above), but against transcript sequence information downloaded from the OneKP transcriptome dataset using the pre-computed orthogroups (http://jlmwiki.plantbio.uga.edu/onekp/v2/, sequence sets for *matK*: ATCG00040_7545.fna, *rpoB*: ATCG00190_1646.fna, and for *ndhB*: ATCG00890_1572.fna).

Phylogenetic analysis was performed as in Oldenkott et al. (2020). Briefly, QED1 orthologs were identified by blastp searches using the protein sequence of *A. thaliana* QED1 (AT2G29760.1 as downloaded from TAIR, version 10; (Huala et al. 2001)) against NCBI's NR protein set (E-value < 1e-10; otherwise default blastp parameters were used, except for setting parameter “max_target_seqs” to a large enough value (500,000) to allow all hits to be reported, and only one HSP per sequence hit was processed (max_hsp = 1)). The initial blastp search returned 112,944 protein hit sequences (E-value < 1e-10). Further filtering by requiring an HSP length of greater than 738/2 (length of *Arabidopsis* QED1 = 738aa) left 93,925 high-confidence blastp hits as candidate orthologs. In cases of multiple candidate ortholog proteins in a given hit species, the one with the lowest E-value was taken as the prime-ortholog candidate of QED1 in that species. Among hits with identical E-values (E-value reported as zero), the hit sequence associated with longest alignment (high-scoring pair, HSP) was taken. This process yielded candidate QED1 orthologs in 582 species.

To identify genome-encoded “pre-edited” leucine codons corresponding to amino acid position 291 of NdhB, we searched for tblastn hits of the pre-edited version (S to L) of a 19 amino acid-peptide corresponding to *A. thaliana* NdhB centered on the editing site (“RIFDIPFYFLSNEWHLLLE”; leucine in question underlined) within the genomic sequences of *ndhB* in 3,683 species obtained from the CpGDB database (Singh et al. 2020) comprising 7,159 (2,351 unique) *ndhB* gene sequences. A relaxed E-value threshold was applied (E-value = 1) to allow for diverged sequences to be reported. Only four species (*Chimonanthus nitens*, *Carex neurocarpa*, *Carex siderosticta,* and *Lens culinaris*) encode in their plastid genomes a leucine codon at the site corresponding to *ndhB*-291 (supplementary table S1, Supplementary Material online).

The RNA secondary structure of Domain I of the intronic sequence of *rps12* (supplementary fig. S1, Supplementary Material online) was predicted with the RNAfold webserver (http://rna.tbi.univie.ac.at//cgi-bin/RNAWebSuite/RNAfold.cgi; (Hofacker 2009)) using standard settings.

Pairwise sequence similarity was calculated using Lola (Tillich et al. 2006). A sequence stretch of 100 nt (from −50 to +50) surrounding the 24 QED1 targets was considered for the analysis, and the window parameter was set to 14 nt. Position 0 (the editing site; [C] context) or position −1 to +1 ([nCn] context) were excluded to avoid potential biases towards particular edited codons. Only targets whose editing efficiency was ≥5% in RNA-seq were considered in the analyses. DNA and protein alignments were performed using the ClustalW option in BioEdit (http://www.mbio.ncsu.edu/BioEdit/bioedit.html). Heat maps and sequence logos were generated using standard parameters in Multiple Array Viewer (MeV) v4.9 (http://compbio.dfci.harvard.edu/compbio/tools/mev) and WebLogo (Crooks et al. 2004), respectively. MassARRAY® data were evaluated using the Typer software (Agena).

## Supplementary Material

msac222_Supplementary_Data

## Data Availability

RNA-seq data have been deposited as fastq files representing the raw RNA sequencing data with the National Center for Biotechnology Information (NCBI) under BioProject accession number PRJNA629102.
