# Supplementary material for: Emergence of Novel RNA-Editing Sites by Changes in the Binding Affinity of a Conserved PPR Protein
Source: Mol Biol Evol. 2022 Oct 13;39(12):msac222. doi: 10.1093/molbev/msac222 (PMC9750133; doi:10.1093/molbev/msac222)
Supplement: msac222_Supplementary_Data [file msac222_supplementary_data.zip › Bock_MBE-22-0655_Supplementary Materials_final.pdf]

SUPPLEMENTARY DATA

Supplementary Table S1. Species encoding “pre-edited” *ndhB*-291 leucine codon.

Supplementary Table S2. *QED1* orthologs and targets. (See Excel file)

Supplementary Table S3. PPR code prediction of QED1 proteins. (See Excel file)

Supplementary Table S4. Editing status of QED1 targets in the *qed1* mutant complemented by chimeric cacao-*Arabidopsis* constructs. (See Excel file)

Supplementary Table S5. Photosynthesis-related parameters of three selected HPL::At-QED1 lines and wild-type *N. tabacum* plants.

Supplementary Table S6. RNA-seq analysis of QED1-expressing tobacco mutants.

Supplementary Table S7. Quantitative real-time PCR (qRT-PCR) analysis. (See Excel file)

Supplementary Table S8. Analysis of the nucleotide sequences surrounding *Arabidopsis* QED1 targets.

Supplementary Table S9. Analyses of *petB* splicing by RNA-seq.

Supplementary Table S10. Localization prediction of QED1.

Supplementary Table S11. List of constructs and transgenic lines used in this study.

Supplementary Table S12. List of oligonucleotides used in this study.

Supplementary Table S13. iPLEX&MassARRAY® assays designed in this study. (See Excel file)

Supplementary Table S14. Coverage of identified SNPs in the RNA-seq libraries.

Supplementary Figure S1. *In silico* folding of Domain I of the *trans*-spliced *rps12* intron 1.

Supplementary Figure S2. Sequence alignment of the *accD* 3´ UTR from various species.

Supplementary Figure S3. Partial sequence alignment of *rps12* intron 1.

Supplementary Figure S4. Non-functional chimeric QED1 proteins.

Supplementary Figure S5. Chloroplast editotype of *Arabidopsis* QED1-expressing transgenic tobacco plants.

Supplementary Figure S6. Validation of selected QED1 off-targets by Sanger sequencing.

Supplementary Figure S7. *QED1* expression analysis by qRT-PCR.

Supplementary Figure S8. Northern blot analyses of *psbB* operon transcripts.

Supplementary Figure S9. An off-target binding site of *Arabidopsis* QED1 in the *ycf10-petA* intergenic region.

Supplementary Figure S10. Editing at *ccmC*-83 by the cacao QED1 protein.

Supplementary Figure S11. Variegated leaf phenotype of transplastomic pRB58 lines expressing the *Arabidopsis* QED1 protein.

Supplementary Dataset S1. Data from array-based ribosome profiling of HPL::At-QED1 lines and wild-type tobacco plants. See Excel file.

Supplementary Dataset S2. Sequences of chimeric QED1 variants shown in Table 1.

Supplementary Figure S1. *In silico* folding of Domain I of the *trans*-spliced *rps12* intron 1.
The RNA secondary structure of Domain I of the intronic sequence of *rps12* was predicted using the RNAfold webserver (http://rna.tbi.univie.ac.at//cgi-bin/RNAWebSuite/RNAfold.cgi; (Hofacker 2009)). The optimal secondary structure shown was predicted with a free energy of folding of -94.7 kcal/mol. The editing site is highlighted in red and resides within a predicted loop/non-basepaired region.

Supplementary Figure S2. Sequence alignment of the *accD* 3´ UTR from various species.
Alignment of the nucleotide sequence 200 bp downstream of the stop codon of *accD* from selected . Conserved residues are shaded in grey (identity threshold: 50%). The position corresponding to *Arabidopsis* editing site *accD*_3UTR (nucleotide position 58,642 in NC_000932.1) is marked by an asterisk. The box marks the predicted binding site of *Arabidopsis* QED1 (based on the sequence of the *Arabidopsis accD*_3UTR site).

Supplementary Figure S3. Partial sequence alignment of *rps12* intron 1.
Alignment of the nucleotide sequence 200 bp downstream of the first exon of *rps12* from selected . Conserved residues are shaded in grey (identity threshold: 50%). The position corresponding to *Arabidopsis* editing site *rps12*_i1 (nucleotide position 69,553 in NC_000932.1) is marked by an asterisk. The box marks the predicted binding site of *Arabidopsis* QED1 (based on the sequence of the *Arabidopsis rps12*_i1 site).

Supplementary Figure S4. Non-functional chimeric QED1 proteins.
Partial alignment of QED1 amino acid sequences from selected species. The last PPR motif (S2, motif no. 15) and the E1 domain annotated based on (Cheng, et al. 2016) are indicated by black horizontal lines. Black arrows indicate the first amino acid of the E1 domain based on the most recent annotation (Cheng, et al. 2016) (*Arabidopsis* residue 534) and of the first amino acid of the previously annotated E domain (Lurin, et al. 2004) (*Arabidopsis* residue 536), respectively. Note that amino acids at positions 535-536 of *Arabidopsis* and amino acids at positions 530-531 of *T. cacao* were included as part of the PPR tract in our constructs (Table 1). Cacao carries a negatively charged aspartate residue (D) while *Arabidopsis* carries a threonine (T) at position 534 (first amino acid of the E1 domain). An aspartate at the beginning of E1 is not found in any other QED1. Hence, it seems possible that this residue is incompatible with the C-terminus of *Arabidopsis* QED1, resulting in non-functional At_Cterm and At_PPR1-8+Cterm proteins (Table 1).

Supplementary Figure S5. Chloroplast editotype of *Arabidopsis* QED1-expressing transgenic tobacco plants.
The complete chloroplast editotypes of the QED1-expressing transgenic lines and wild-type tobacco plants were assessed using the iPLEX/MassARRAY® technology (see Methods). Pale, variegated (var) and green tissue from the UBQ::At-QED1 overexpression lines was separately harvested and analysed. Data are presented with Multiple Array Viewer (MeV). The color scale indicates the editing percentage (T/C signal ratio) from 0% (red) to 100% (green). Grey: failed assay. Values for the QED1 mutants represent averages of two technical replicates. Values for the wild type (WT) represent averages of five biological replicates. Three previously generated pale mutants (Δ*atpB*, Δ*ycf3* and WX7; see Material and Methods for a detailed description of the lines) were included as controls for the pale phenotype.

Supplementary Figure S6. Validation of selected QED1 off-targets by Sanger sequencing.
Seven plastid and one mitochondrial off-target sites of *Arabidopsis* QED1 in tobacco identified by RNA-seq in tobacco (Table 2) were validated by bulk sequencing of amplified cDNA in one HPL and one UBQ line. Asterisks indicate C-to-U conversions by off-target editing.

Supplementary Figure S7. *QED1* expression analysis by qRT-PCR.
Relative expression of transgenic tobacco lines expressing *Arabidopsis*-QED1 (At-QED1) quantified by real-time PCR (qRT-PCR). Because the target gene is only present in the transgenic lines and not in wild-type plants, relative expression values are reported as ΔΔCt fold changes to the transgenic line with the highest ΔCt value, hence, the lowest expression level (HPL::At-QED1#16-4 for *Arabidopsis* QED1). Raw data and calculations are provided in Supplementary Table S7.

Supplementary Figure S8. Northern blot analyses of *psbB* operon transcripts.
Single-stranded RNA probes were designed against the coding regions of *psbH*, *psbB*, *petB* and *petD*, and the intron sequence of *petD* (*petD-i*). An additional probe was generated for the intron sequence of *petB*, but yielded only low-intensity signals of poor quality.Young emerging green leaves were harvested from six week-old wild-type tobacco (WT) and HPL lines (HPL::At-QED1#1-1 and HPL::At-QED1#11-04-3 shown in Figure 5D and E). Samples of 5 µg total RNA were loaded. Sizes of marker bands are given in kb. Methylene blue (MB) staining of an rRNA-containing part of each blot is shown as loading control.

Supplementary Figure S9. An off-target binding site of *Arabidopsis* QED1 in the *ycf10-petA* intergenic region.
Nucleotide sequence alignment of the *ycf10-petA* region from *N. tabacum*, *A. thaliana* and *Z. mays*. The position corresponding to the off-target editing site *ycf10-petA* edited by *Arabidopsis* QED1 in tobacco (Table 2) is indicated by the asterisk. The predicted QED1 binding site is boxed. Note that no other potential QED1 binding sites was found, despite using a degenerated consensus in the search. In addition to the spacer sequence, the last 100 nt of *ycf10* and the first 100 nt of *petA* are included in the alighment. Residues differing between the three species are shaded in grey.

Supplementary Figure S10. Editing at *ccmC*-83 by the cacao QED1 protein.

Editing at the mitochondrial *ccmC*-83 off-target site by the cacao QED1 (Tc-QED1) was evaluated by bulk sequencing in two independent HPL and two independent UBQ lines. Note that lines UBQ::Tc-QED1#2 and #5 were analyzed by iPLEX/MassARRAY® for editing at chloroplast off-target sites (Table 5). The asterisk indicates the partial C-to-U conversion at site *ccmC*-83.

Supplementary Figure S11. Variegated leaf phenotype of transplastomic pRB58 lines expressing the *Arabidopsis* QED1 protein.

Close-up of the pRB58+UBQ::At-QED1 lines shown in Figure 9B. Overexpression of QED1 resulted in the appearance of pale and necrotic spots on leaves. Photograph was taken 16 weeks after sowing. Scale bar: 1 cm.

Supplementary Table S1. Species encoding a "pre-edited" *ndhB*-291 leucine codon. Leucine (L) codon in question is marked in bold red. See Materials and Methods for details.

ndbB-291 "pre-edited" L hits	Sequence
CHNI - Chimonanthus_nitens 141896..144128	RIFDIPFYF**L**SNEWHLLLE
CASD - Carex_siderosticta 114320..116542	RIFDIPF F**L**SNEWHLLLE
CANE - Carex_neurocarpa 160202..162424	RIFDIPF F**L**SNEWHLLLE
LECU - Lens_culinaris 90588..92751	RI DIPFYF**L**SNEWHLLLE

Supplementary Table S5. Photosynthesis-related parameters of three selected HPL::At-QED1 lines and wild-type *N. tabacum* plants.
Significant changes between wild type and mutants are marked in bold. Between mutant lines no significant differences were obtained except for the parameters indicated in italics. One-way ANOVA, Holm–Sidak method, $P \leq 0.05$.

Parameter	Wild Type	HPL::At-QED1#1	HPL::At-QED1#11	HPL::At-QED1#16
Chlorophyll *a/b*	4.53±0.11	**4.18±0.1**	**4.27±0.07**	4.39±0.14
Chlorophyll [mg m^{-2}]	481.6±63.7	551.2±30.6	463.8±35.4	474.9±53.2
Leaf absorptance (%)	86.6±2.6	87.3±0.6	86.8±0.6	84.8±0.5
F_v/F_m	0.81±0.01	0.79±0.01	***0.75±0.02***	0.8±0.01
Membrane conductivity [s^{-1}]	41.3±2	42.3±3.3	***52.5±7.1***	44.1±4
n	5	5	6	5

Supplementary Table S6. RNA-seq analysis of QED1-expressing tobacco mutants.
Summary of the reads obtained from RNA-seq analyses for each library and each strand. R1 and R2 correspond to reverse and forward reads, respectively. Reads that passed quality filtering (QF) and mapped to the chloroplast (cp) and mitochondrial (mt) genomes are indicated (see Methods for details). The analyzed plant lines are ordered based on the observed phenotype (from wild type-like to strong leaf variegation). Cytochrome b_6f complex contents (in %) were remeasured for HPL::At-QED1#1 and HPL::At-QED1#6.

		**Raw reads**			**QF reads**				**Organellar reads**			
**Genotype**	**Phenotype**	R1	R2	**total**	R1	R2	**total**	**% QF**	R1	R2	**total**	**% cp-mt**
WT #1	wild-type	29,614,426	29,614,426	59,228,852	29,253,789	26,622,814	55,876,603	94.3	17,872,809	16,300,706	34,173,515	61.2
WT #2	wild-type	26,669,508	26,669,508	53,339,016	26,269,251	23,785,040	50,054,291	93.8	16,205,628	14,688,283	30,893,911	61.7
HPL::At-QED1 #6	57.8% cyt-b_6f	26,910,172	26,910,172	53,820,344	26,556,504	23,783,868	50,340,372	93.5	16,636,677	14,930,476	31,567,153	62.7
HPL::At-QED1 #1	55.3% cyt-b_6f	29,067,639	29,067,639	58,135,278	28,669,045	25,678,578	54,347,623	93.5	17,842,202	15,999,956	33,842,158	62.3
UBQ::At-QED1 #3	sterile, slow growth	24,962,298	24,962,298	49,924,596	24,652,472	22,043,504	46,695,976	93.5	16,693,899	14,973,413	31,667,312	67.8
UBQ::At-QED1 #1	variegated, sugar-dependent	47,918,930	47,918,930	95,837,860	47,234,261	42,142,181	89,376,442	93.3	32,278,983	28,871,268	61,150,251	68.4

Supplementary Table S8. Analysis of the nucleotide sequences surrounding *Arabidopsis* QED1 targets.
The 24 targets of *Arabidopsis* QED1 that were edited above 10% in at least one RNA-seq library (Table 3) were analyzed with respect to the neighboring nucleotides (at positions -1 and +1; Context). Maximum editing (in %) refers to the highest SNP ratios detected in the RNA-seq libraries of the transgenic tobacco lines expressing the Arabidopsis QED1 protein (cf. Table 2).

Context	No. sequences	Max editing %
**ACA**	**8**	**72.2**
ACC	0	-
**ACG**	**2**	**32.9**
**ACU**	**1**	**15.0**
**CCA**	**1**	**87.4**
CCC	0	-
**CCG**	**1**	**20.3**
CCU	0	-
**GCA**	**2**	**26.8**
**GCC**	**1**	**13.7**
CCG	0	-
CCU	0	-
**UCA**	**8**	**91.8**
UCC	0	-
UCG	0	-
UCU	0	-
**nCA**	**19**	**91,8**
**nCC**	**1**	**13.7**
**nCG**	**3**	**32.9**
**nCU**	**1**	**15.0**
**ACn**	**11**	**72.2**
**CCn**	**2**	**87.4**
**GCn**	**3**	**26.8**
**UCn**	**8**	**91.8**
**YCn**	**10**	**91.8**
**RCn**	**14**	**72.2**
**nCY**	**2**	**15.0**
**nCR**	**22**	**91.8**

Supplementary Table S9. Analyses of *petB* splicing by RNA-seq.

The expression of *petB* in the HPL lines and wildtype (WT) tobacco was analyzed by mapping QF reads specifically to the exon1-exon2, exon1-intron or intron-exon2 junction. The mapped reads were then normalized to the total number of mapped organellar reads (estimated expression reported as mapped *petB* reads per mill organellar reads). The rate of *petB* splicing was calculated as ratio of spliced/unspliced reads.

***petB* expression**			**HPL::At-QED1**		**WT**	
			#1	#6	#1	#2
**spliced**	exon1-exon2	reads	3,557	3,195	4,241	3,364
		estimated expression	0.105	0.101	0.124	0.109
**unspliced**	exon1-intron	reads	1,255	1,075	1,488	1,299
		estimated expression	0.037	0.034	0.044	0.042
	intron-exon2	reads	1,560	1,323	1,405	1,200
		estimated expression	0.046	0.042	0.041	0.039
		**% spliced exon1**	**73.9**	**74.8**	**74.0**	**72.1**
		**% spliced exon2**	**69.5**	**70.7**	**75.1**	**73.7**

Supplementary Table S10. Localization prediction of QED1.

Localization Database	Reference	Predicted localization
TargetP - 2.0	Armenteros *et al.*, 2019 (doi: 10.26508/lsa.201900429)	Chloroplast
WoLF PSORT	Horton *et al.*, 2007 (doi: 10.1093/nar/gkm259)	Chloroplast
SignalP - 6.0	Teufel *et al.*, 2022 (doi: 10.1038/s41587-021-01156-3)	Other
SUBA	Hooper *et al.*, 2017 (doi: 10.1093/nar/gkw1041)	Plastid
Predotar	Small *et al.*, 2004 (doi: 10.1002/pmic.200300776)	Plastid

Supplementary Table S11. List of constructs and transgenic lines used in this study.

Construct	Construct name used in this work	Description	Transgene	Transgene promoter	Bacterial resistance	Plant resistance	Cloning strategy	Target for plant transformation	Name of transgenic lines
**Transgenic lines generated in this work**									
**Tobacco**									
**pVL2**	HPL::At-QED1	Full length *A. thaliana* Col-0 *QED1* CDS expressed from the *HPL* promoter	*At-QED1*	*HPL*	*nptII* (kanamycin)	*nptII* (kanamycin)	Full length CDS of *QED1* (AT2G29760) amplified from *A. thaliana* Col-0 genomic DNA using primers B11 and B12 and cloned as SpeI/KpnI fragment in pORE-E2	*N. tabacum* spp. Petit Havana<br>pRB58 (Bock *et al.*, 1996)	**HPL::At-QED1**<br>**pRB58+ HPL::At-**
**pVL4**	UBQ::At-QED1	Full length *A. thaliana* Col-0 *QED1* CDS expressed from the *UBQ* promoter		*UBQ*			The *UBIQUITIN 10* promoter was amplified from *Arabidopsis* Col-0 genomic DNA using primers P_UBQ10for and P_UBQ10rev and cloned as XhoI/EcoRI fragment in pVL2	*N. tabacum* spp. Petit Havana<br>pRB58 (Bock *et al.*, 1996)	**UBQ::At-QED1**<br>**pRB58+ UBQ::At-**
**pVL17**	HPL::Tc-QED1	Full length *T. cacao QED1* CDS expressed from the *HPL* promoter	*Tc-QED1*	*HPL*		*pat* (BASTA)	Cacao *QED1* (TC0009G29800) was amplified from *T. cacao* genomic DNA using primers oVL66 and oVL67 and cloned by Gibson Assembly® into pVL14 linearized with KpnI	*N. tabacum* spp. Petit Havana	**HPL::Tc-QED1**
**pVL27**	UBQ::Tc-QED1	Full length *T. cacao QED1* CDS expressed from the *UBQ* promoter		*UBQ*			Cacao *QED1* (TC0009G29800) was amplified from *T. cacao* genomic DNA using primers oVL66 and oVL67 and cloned by Gibson Assembly® into pVL15 linearized with KpnI		**UBQ::Tc-QED1**
Arabidopsis									
**pVL18**	HPL:: At_Cterm	*A. thaliana-T. cacao* QED1 chimera constructed based on PPR annotation from Lurin *et al.*, 2004: *RbcS* transit peptide, 3x glycine linker, *T. cacao* PPR tract from first weakly predicted motif (a.a. #21) to motif 14 (a.a. #530), *A. thaliana* C-terminus (from a.a. #536 to #739) expressed from the *HPL* promoter	*At_Cterm*	*HPL*	*nptII* (kanamycin)	*pat* (BASTA)	*P. sativum RbcS* transit peptide-encoding sequence was amplified from pIT42 using primers oVL80 and otHA6 (including 3x glycine linker); *T. cacao QED1* PPR tract (motifs 1-14) was amplified from *T. cacao* genomic DNA using primers Tc-09 and Tc-10; *A. thaliana QED1* C-terminus was amplified from *A. thaliana* Col-0 genomic DNA using primers ot-05 and oVL81. Fragments were assembled by Gibson Assembly® into pVL14, linearized with KpnI	*qed1*-2 (Wagoner *et al.*, 2015)	***qed1*-2 + HPL::At_Cterm**
**pVL23**	UBQ:: At_Cterm	*A. thaliana-T. cacao* QED1 chimera constructed based on PPR annotation from Lurin *et al.*, 2004: *RbcS* transit peptide, 3x glycine linker, *T. cacao* PPR tract from first weakly predicted motif (a.a. #21) to motif 14 (a.a. #530), *A. thaliana* C-terminus (from a.a. #536 to #739) expressed from the *UBQ* promoter		*UBQ*			*P. sativum RbcS* transit peptide-encoding sequence was amplified from pIT42 using primers oVL80 and otHA6 (including 3x glycine linker); *T. cacao QED1* PPR tract (motifs 1-14) was amplified from *T. cacao* genomic DNA using primers Tc-09 and Tc-10; *A. thaliana QED1* C-terminus was amplified from *A. thaliana* Col-0 genomic DNA using primers ot-05 and oVL81. Fragments were assembled by Gibson Assembly® into pVL15, linearized with KpnI		***qed1*-2 + UBQ::At_Cterm**
**pVL21**	HPL:: At_PPR1-15	*A. thaliana-T. cacao* QED1 chimera constructed based on PPR annotation from Lurin *et al.*, 2004: *RbcS* transit peptide, 3x glycine linker, *A. thaliana* PPR tract from first weakly predicted motif (a.a. #26) to motif 14 (a.a. #535), *T. cacao* C-terminus (from a.a. #531 to #734) expressed from the *HPL* promoter	*At_PPR1-15*	*HPL*			*P. sativum RbcS* transit peptide-encoding sequence was amplified from pIT42 using primers oVL80 and otHA7 (including 3x glycine linker); *A. thaliana QED1* PPR tract (motif 1-14) was amplified from *A. thaliana* Col-0 genomic DNA using primers ot-08 and ot-10, *T. cacao* C-terminus was amplified from *T. cacao* genomic DNA using primers Tc-13 and oVL67. Fragments were assembled by Gibson Assembly® into pVL14, linearized with KpnI		***qed1*-2 + HPL::At_PPR1-15**

Construct	Construct name used in this work	Description	Transgene	Transgene promoter	Bacterial resistance	Plant resistance	Cloning strategy	Target for plant transformation	Name of transgenic lines
**pVL26**	UBQ:: At_PPR1-15	*A. thaliana-T. cacao* QED1 chimera constructed based on PPR annotation from Lurin *et al.*, 2004: *RbcS* transit peptide, 3x glycine linker, *A. thaliana* PPR tract from first weakly predicted motif (a.a. #26) to motif 14 (a.a. #535), *T. cacao* C-terminus (from a.a. #531 to #734) expressed from the *UBQ* promoter	*At_PPR1-15*	*UBQ*	*nptII* (kanamycin)	*pat* (BASTA)	*P. sativum RbcS* transit peptide-encoding sequence was amplified from pIT42 using primers oVL80 and otHA7 (including 3x glycine linker); *A. thaliana QED1* PPR tract (motifs 1-14) was amplified from *A. thaliana* Col-0 genomic DNA using primers ot-08 and ot-10; *T. cacao* C-terminus was amplified from *T. cacao* genomic DNA using primers Tc-13 and oVL67. Fragments were assembled by Gibson Assembly® into pVL15, linearized with KpnI	*qed1*-2 (Wagoner *et al.*, 2015)	***qed1*-2 + UBQ::At_PPR1-15**
**pVL19**	HPL:: At_PPR9-15+Cterm	*A. thaliana-T. cacao* QED1 chimera constructed based on PPR annotation from Lurin *et al.*, 2004: *RbcS* transit peptide, 3x glycine linker, *T. cacao* PPR tract from first weakly predicted motif (a.a. #21) to motif 7 (a.a. #291), *A. thaliana* PPR tract from motif 8 to 14 and C-terminus (from a.a. #536 to #739) expressed from the *HPL* promoter	*At_PPR9-15+Cterm*	*HPL*			*P. sativum RbcS* transit peptide-encoding sequence was amplified from pIT42 using primers oVL80 and otHA6 (including 3x glycine linker); *T. cacao QED1* PPR tract (motifs 1-7) was amplified from *T. cacao* genomic DNA using primers Tc-09 and Tc-11; *A. thaliana QED1* PPR tract (from motif 8) and C-terminus was amplified from *A. thaliana* Col-0 genomic DNA using primers ot-07 and oVL81. Fragments were assembled by Gibson Assembly® into pVL14, linearized with KpnI		***qed1*-2 + HPL::At_PPR9-15+Cterm**
**pVL24**	UBQ::At_PPR9-15+Cterm	*A. thaliana-T. cacao* QED1 chimera constructed based on PPR annotation from Lurin *et al.*, 2004: *RbcS* transit peptide, 3x glycine linker, *T. cacao* PPR tract from first weakly predicted motif (a.a. #21) to motif 7 (a.a. #291), *A. thaliana* PPR tract from motif 8 to 14 and C-terminus (from a.a. #536 to #739) expressed from the *UBQ* promoter	*At_PPR9-15+Cterm*	*UBQ*			*P. sativum RbcS* transit peptide-encoding sequence was amplified from pIT42 using primers oVL80 and otHA6 (including 3x glycine linker); *T. cacao QED1* PPR tract (motifs 1-7) was amplified from *T. cacao* genomic DNA using primers Tc-09 and Tc-11; *A. thaliana QED1* PPR tract (from motif 8) and C-terminus was amplified from *A. thaliana* Col-0 genomic DNA using primers ot-07 and oVL81. Fragments were assembled by Gibson Assembly® into pVL15, linearized with KpnI		***qed1*-2 + UBQ::At_PPR9-15+Cterm**
**pVL20**	HPL::At_PPR1-8+Cterm	*A. thaliana-T. cacao* QED1 chimera constructed based on PPR annotation from Lurin *et al.*, 2004: *RbcS* transit peptide, 3x glycine linker, *A. thaliana* PPR tract from first weakly predicted motif (a.a. #26) to motif 7 (a.a. #296), *T. cacao* PPR tract from motif 8 to 14 (from a.a. #292 to a.a. #530), *A. thaliana* C-terminus (from a.a. #536 to #739) expressed from the *HPL* promoter	*At_PPR1-8+Cterm*	*HPL*			*P. sativum RbcS* transit peptide-encoding sequence was amplified from pIT42 using primers oVL80 and otHA7 (including 3x glycine linker); *A. thaliana QED1* PPR tract (motifs 1-7) was amplified from *A. thaliana* Col-0 genomic DNA using primers ot-08 and ot-09; *T. cacao QED1* PPR tract (motifs 8-14) was amplified from *T. cacao* genomic DNA using primers Tc-12 and Tc-10; *A. thaliana QED1* C-terminus was amplified from *A. thaliana* Col-0 genomic DNA using primers ot-05 and oVL81. Fragments were assembled by Gibson Assembly® into pVL14, linearized with KpnI		***qed1*-2 + HPL::At_PPR1-8+Cterm**

Construct	Construct name used in this work	Description	Transgene	Transgene promoter	Bacterial resistance	Plant resistance	Cloning strategy	Target for plant transformation	Name of transgenic lines
**pVL25**	UBQ::At_PPR1-8+Cterm	*A. thaliana-T. cacao* QED1 chimera constructed based on PPR annotation from Lurin *et al.*, 2004: *RbcS* transit peptide, 3x glycine linker, *A. thaliana* PPR tract from first weakly predicted motif (a.a. #26) to motif 7 (a.a. #296), *T. cacao* PPR tract from motif 8 to 14 (from a.a. #292 to a.a. #530), *A. thaliana* C-terminus (from a.a. #536 to #739) expressed from the *UBQ* promoter	*At_PPR1-8+Cterm*	*UBQ*	*nptII* (kanamycin)	*pat* (BASTA)	*P. sativum RbcS* transit peptide-encoding sequence was amplified from pIT42 using primers oVL80 and otHA7 (including 3x glycine linker); *A. thaliana QED1* PPR tract was amplified (motifs 1-7) from *A. thaliana* Col-0 genomic DNA using primers ot-08 and ot-09, *T. cacao QED1* PPR tract (motifs 8-14) was amplified from *T. cacao* genomic DNA using primers Tc-12 and Tc-10; *A. thaliana QED1* C-terminus was amplified from *A. thaliana* Col-0 genomic DNA using primers ot-05 and oVL81. Fragments were assembled by Gibson Assembly® into pVL15, linearized with KpnI	*qed1*-2 (Wagoner *et al.*, 2015)	***qed1*-2 + UBQ::At_PPR1-8+Cterm**
**pVL39**	HPL::At_PPR9-15	*A. thaliana-T. cacao* QED1 chimera constructed based on PPR annotation from Lurin *et al.*, 2004: *RbcS* transit peptide, 3x glycine linker, *T. cacao* PPR tract from first weakly predicted motif (a.a. #21) to motif 7 (a.a. #291), *A. thaliana* PPR tract from motif 8 (a.a. #297) to motif 14 (a.a. # 535), *T. cacao* C-terminus (from a.a. #531 to #734) expressed from the *HPL* promoter	*At_PPR9-15*	*HPL*	*nptII* (kanamycin)	*pat* (BASTA)	*P. sativum RbcS* transit peptide-encoding sequence was amplified from pIT42 using primers oVL80 and otHA6 (including 3x glycine linker); *T. cacao QED1* PPR tract (motif 1-7) was amplified from *T. cacao* genomic DNA using primers Tc-09 and Tc-11, *A. thaliana* PPR tract (motifs 8-14) was amplified from *A. thaliana* Col-0 genomic DNA using primers ot-07 and ot-10, *T. cacao* C-terminus was amplified from *T. cacao* genomic DNA using primers Tc-13 and oVL67. Fragments were assembled by Gibson Assembly® into pVL14, linearized with KpnI	*qed1*-2 (Wagoner *et al.*, 2015)	***qed1*-2 + HPL::At_PPR9-15**
**pVL40**	UBQ::At_PPR9-15	*A. thaliana-T. cacao* QED1 chimera constructed based on PPR annotation from Lurin *et al.*, 2004: *RbcS* transit peptide, 3x glycine linker, *T. cacao* PPR tract from first weakly predicted motif (a.a. #21) to motif 7 (a.a. #291), *A. thaliana* PPR tract from motif 8 (a.a. #297) to motif 14 (a.a. # 535), *T. cacao* C-terminus (from a.a. #531 to #734) expressed from the *UBQ* promoter	*At_PPR9-15*	*UBQ*	*nptII* (kanamycin)	*pat* (BASTA)	*P. sativum RbcS* transit peptide-encoding sequence was amplified from pIT42 using primers oVL80 and otHA6 (including 3x glycine linker); *T. cacao QED1* PPR tract (motif 1-7) was amplified from *T. cacao* genomic DNA using primers Tc-09 and Tc-11, *A. thaliana* PPR tract (motifs 8-14) was amplified from *A. thaliana* Col-0 genomic DNA using primers ot-07 and ot-10, *T. cacao* C-terminus was amplified from *T. cacao* genomic DNA using primers Tc-13 and oVL67. Fragments were assembled by Gibson Assembly® into pVL15, linearized with KpnI	*qed1*-2 (Wagoner *et al.*, 2015)	***qed1*-2 + UBQ::At_PPR9-15**
**pVL41**	HPL::At_PPR9-11	*A. thaliana-T. cacao* QED1 chimera constructed based on PPR annotation from Cheng *et al.*, 2016: *RbcS* transit peptide, 3x glycine linker, *T. cacao* PPR tract from first motif to motif 8, *A. thaliana* PPR motif 9, 10 and 11, *T. cacao* PPR tract from motif 12 to 15 and C-terminus expressed from the *HPL* promoter	*At_PPR9-11*	*HPL*	*nptII* (kanamycin)	*pat* (BASTA)	*P. sativum RbcS* transit peptide-encoding sequence was amplified from pIT42 using primers oVL80 and otHA6 (including 3x glycine linker); *T. cacao QED1* PPR tract (motif 1-8) was amplified from *T. cacao* genomic DNA using primers Tc-09 and Tc-11, *A. thaliana* PPR motifs 9 to 11 were amplified from *A. thaliana* Col-0 genomic DNA using primers ot-07 and oVL126, *T. cacao* PPR motifs 12 to 15 and C-terminus were amplified from *T. cacao* genomic DNA using primers oVL127 and oVL67. Fragments were assembled by Gibson Assembly® into pVL14, linearized with KpnI	*qed1*-2 (Wagoner *et al.*, 2015)	***qed1*-2 + HPL::At_PPR9-11**

<table>
<tr><th>Construct</th><th>Construct name used in this work</th><th>Description</th><th>Transgene</th><th>Transgene promoter</th><th>Bacterial resistance</th><th>Plant resistance</th><th>Cloning strategy</th><th>Target for plant transformation</th><th>Name of transgenic lines</th></tr>
<tr><td>pVL42</td><td>UBQ::At_PPR9-11</td><td>A. thaliana-T. cacao QED1 chimera constructed based on PPR annotation from Cheng et al., 2016: RbcS transit peptide, 3x glycine linker, T. cacao PPR tract from first motif to motif 8, A. thaliana PPR motif 9, 10 and 11, T. cacao PPR tract from motif 12 to 15 and C-terminus expressed from the UBQ promoter</td><td>At_PPR9-11</td><td>UBQ</td><td rowspan="4">nptII (kanamycin)</td><td rowspan="4">pat (BASTA)</td><td>P. sativum RbcS transit peptide-encoding sequence was amplified from pIT42 using primers oVL80 and otHA6 (including 3x glycine linker); T. cacao QED1 PPR tract (motif 1-8) was amplified from T. cacao genomic DNA using primers Tc-09 and Tc-11, A. thaliana PPR motifs 9 to 11 were amplified from A. thaliana Col-0 genomic DNA using primers ot-07 and oVL126, T. cacao PPR motifs 12 to 15 and C-terminus were amplified from T. cacao genomic DNA using primers oVL127 and oVL67. Fragments were assembled by Gibson Assembly® into pVL15, linearized with KpnI</td><td rowspan="4">qed1-2 (Wagoner et al., 2015)</td><td>qed1-2 + UBQ::At_PPR9-11</td></tr>
<tr><td>pVL49</td><td>HPL::At_PPR9-10</td><td>A. thaliana-T. cacao QED1 chimera constructed based on PPR annotation from Cheng et al., 2016: RbcS transit peptide, 3x glycine linker, T. cacao PPR tract from first motif to motif 8, A. thaliana PPR motif 9 and 10, T. cacao PPR tract from motif 11 to 15 and C-terminus expressed from the HPL promoter</td><td rowspan="2">At_PPR9-10</td><td>HPL</td><td>P. sativum RbcS transit peptide-encoding sequence was amplified from pIT42 using primers oVL80 and otHA6 (including 3x glycine linker); T. cacao QED1 PPR tract (motif 1-8) was amplified from T. cacao genomic DNA using primers Tc-09 and Tc-11, A. thaliana PPR motifs 9 to 10 were amplified from A. thaliana Col-0 genomic DNA using primers ot-07 and oVL132, T. cacao PPR motifs 11 to 15 and C-terminus were amplified from T. cacao genomic DNA using primers oVL133 and oVL67. Fragments were assembled by Gibson Assembly® into pVL14, linearized with KpnI</td><td>qed1-2 + HPL::At_PPR9-10</td></tr>
<tr><td>pVL50</td><td>UBQ::At_PPR9-10</td><td>A. thaliana-T. cacao QED1 chimera constructed based on PPR annotation from Cheng et al., 2016: RbcS transit peptide, 3x glycine linker, T. cacao PPR tract from first motif to motif 8, A. thaliana PPR motif 9 and 10, T. cacao PPR tract from motif 11 to 15 and C-terminus expressed from the UBQ promoter</td><td>UBQ</td><td>P. sativum RbcS transit peptide-encoding sequence was amplified from pIT42 using primers oVL80 and otHA6 (including 3x glycine linker); T. cacao QED1 PPR tract (motif 1-8) was amplified from T. cacao genomic DNA using primers Tc-09 and Tc-11, A. thaliana PPR motifs 9 to 10 were amplified from A. thaliana Col-0 genomic DNA using primers ot-07 and oVL132, T. cacao PPR motifs 11 to 15 and C-terminus were amplified from T. cacao genomic DNA using primers oVL133 and oVL67. Fragments were assembled by Gibson Assembly® into pVL15, linearized with KpnI</td><td>qed1-2 + UBQ::At_PPR9-10</td></tr>
<tr><td>pVL51</td><td>HPL::At_PPR10-11</td><td>A. thaliana-T. cacao QED1 chimera constructed based on PPR annotation from Cheng et al., 2016: RbcS transit peptide, 3x glycine linker, T. cacao PPR tract from first motif to motif 9, A. thaliana PPR motif 10 and 11, T. cacao PPR tract from motif 12 to 15 and C-terminus expressed from the HPL promoter</td><td>At_PPR10-11</td><td>HPL</td><td>P. sativum RbcS transit peptide-encoding sequence was amplified from pIT42 using primers oVL80 and otHA6 (including 3x glycine linker); T. cacao QED1 PPR tract (motif 1-9) was amplified from T. cacao genomic DNA using primers Tc-09 and oVL134, A. thaliana PPR motifs 10 to 11 were amplified from A. thaliana Col-0 genomic DNA using primers oVL135 and oVL132, T. cacao PPR motifs 12 to 15 and C-terminus were amplified from T. cacao genomic DNA using primers oVL133 and oVL67. Fragments were assembled by Gibson Assembly® into pVL14, linearized with KpnI</td><td>qed1-2 + HPL::At_PPR10-11</td></tr>
</table>

Construct	Construct name used in this work	Description	Transgene	Transgene promoter	Bacterial resistance	Plant resistance	Cloning strategy	Target for plant transformation	Name of transgenic lines
**pVL52**	UBQ::At_PPR10-11	*A. thaliana-T. cacao* QED1 chimera constructed based on PPR annotation from Cheng *et al.*, 2016: *RbcS* transit peptide, 3x glycine linker, *T. cacao* PPR tract from first motif to motif 9, *A. thaliana* PPR motif 10 and 11, *T. cacao* PPR tract from motif 12 to 15 and C-terminus expressed from the *UBQ* promoter	*At_PPR10-11*	*UBQ*			*P. sativum RbcS* transit peptide-encoding sequence was amplified from pIT42 using primers oVL80 and otHA6 (including 3x glycine linker); *T. cacao QED1* PPR tract (motif 1-9) was amplified from *T. cacao* genomic DNA using primers Tc-09 and oVL134, *A. thaliana* PPR motifs 10 to 11 were amplified from *A. thaliana* Col-0 genomic DNA using primers oVL135 and oVL132, *T. cacao* PPR motifs 12 to 15 and C-terminus were amplified from *T. cacao* genomic DNA using primers oVL133 and oVL67. Fragments were assembled by Gibson Assembly® into pVL15, linearized with KpnI		***qed1-2* + UBQ::At_PPR10-11**
**pVL53**	HPL::At_PPR9,11	*A. thaliana-T. cacao* QED1 chimera constructed based on PPR annotation from Cheng *et al.*, 2016: *RbcS* transit peptide, 3x glycine linker, *T. cacao* PPR tract from first motif to motif 8, *A. thaliana* PPR motif 9, *T. cacao* PPR motif 10, *A. thaliana* PPR motif 11, *T. cacao* PPR tract from motif 12 to 15 and C-terminus expressed from the *HPL* promoter	*At_PPR9,11*	*HPL*	*nptII* (kanamycin)	*pat* (BASTA)	*P. sativum RbcS* transit peptide-encoding sequence was amplified from pIT42 using primers oVL80 and otHA6 (including 3x glycine linker); *T. cacao QED1* PPR tract (motif 1-8) was amplified from *T. cacao* genomic DNA using primers Tc-09 and Tc-11, *A. thaliana* PPR motif 9 was amplified from *A. thaliana* Col-0 genomic DNA using primers ot-07 and oVL128, *T. cacao* PPR motif 10 was amplified from *T. cacao* genomic DNA using primers oVL129 and oVL130, *A. thaliana* PPR motif 11 was amplified from *A. thaliana* Col-0 genomic DNA using primers oVL131 and oVL126, *T. cacao* PPR motifs 12 to 15 and C-terminus were amplified from *T. cacao* genomic DNA using primers oVL127 and oVL67. Fragments were assembled by Gibson Assembly® into pVL14, linearized with KpnI	*qed1*-2 (Wagoner *et al.*, 2015)	***qed1*-2 + HPL::At_PPR9,11**
**pVL54**	UBQ::At_PPR9,11	*A. thaliana-T. cacao* QED1 chimera constructed based on PPR annotation from Cheng *et al.*, 2016: *RbcS* transit peptide, 3x glycine linker, *T. cacao* PPR tract from first motif to motif 8, *A. thaliana* PPR motif 9, *T. cacao* PPR motif 10, *A. thaliana* PPR motif 11, *T. cacao* PPR tract from motif 12 to 15 and C-terminus expressed from the *UBQ* promoter		*UBQ*			*P. sativum RbcS* transit peptide-encoding sequence was amplified from pIT42 using primers oVL80 and otHA6 (including 3x glycine linker); *T. cacao QED1* PPR tract (motif 1-8) was amplified from *T. cacao* genomic DNA using primers Tc-09 and Tc-11, *A. thaliana* PPR motif 9 was amplified from *A. thaliana* Col-0 genomic DNA using primers ot-07 and oVL128, *T. cacao* PPR motif 10 was amplified from *T. cacao* genomic DNA using primers oVL129 and oVL130, *A. thaliana* PPR motif 11 was amplified from *A. thaliana* Col-0 genomic DNA using primers oVL131 and oVL126, *T. cacao* PPR motifs 12 to 15 and C-terminus were amplified from *T. cacao* genomic DNA using primers oVL127 and oVL67. Fragments were assembled by Gibson Assembly® into pVL15, linearized with KpnI		***qed1*-2 + UBQ::At_PPR9,11**

Construct	Construct name used in this work	Description	Transgene	Transgene promoter	Bacterial resistance	Plant resistance	Cloning strategy	Target for plant transformation	Name of transgenic lines
**pVL43**	HPL::At_PPR9	*A. thaliana-T. cacao* QED1 chimera constructed based on PPR annotation from Cheng *et al.*, 2016: *RbcS* transit peptide, 3x glycine linker, *T. cacao* PPR tract from first motif to motif 8, *A. thaliana* PPR motif 9, *T. cacao* PPR tract from motif 10 to 15 and C-terminus expressed from the *HPL* promoter	*At_PPR9*	*HPL*	*nptII* (kanamycin)	*pat* (BASTA)	*P. sativum RbcS* transit peptide-encoding sequence was amplified from pIT42 using primers oVL80 and otHA6 (including 3x glycine linker); *T. cacao QED1* PPR tract (motif 1-8) was amplified from *T. cacao* genomic DNA using primers Tc-09 and Tc-11, *A. thaliana* PPR motifs 9 was amplified from *A. thaliana* Col-0 genomic DNA using primers ot-07 and oVL128, *T. cacao* PPR motifs 10 to 15 and C-terminus were amplified from *T. cacao* genomic DNA using primers oVL129 and oVL67. Fragments were assembled by Gibson Assembly® into pVL14, linearized with KpnI	*qed1*-2 (Wagoner *et al.*, 2015)	***qed1*-2 + HPL::At_PPR9**
**pVL44**	UBQ::At_PPR9	*A. thaliana-T. cacao* QED1 chimera constructed based on PPR annotation from Cheng *et al.*, 2016: *RbcS* transit peptide, 3x glycine linker, *T. cacao* PPR tract from first motif to motif 8, *A. thaliana* PPR motif 9, *T. cacao* PPR tract from motif 10 to 15 and C-terminus expressed from the *UBQ* promoter		*UBQ*			*P. sativum RbcS* transit peptide-encoding sequence was amplified from pIT42 using primers oVL80 and otHA6 (including 3x glycine linker); *T. cacao QED1* PPR tract (motif 1-8) was amplified from *T. cacao* genomic DNA using primers Tc-09 and Tc-11, *A. thaliana* PPR motifs 9 was amplified from *A. thaliana* Col-0 genomic DNA using primers ot-07 and oVL128, *T. cacao* PPR motifs 10 to 15 and C-terminus were amplified from *T. cacao* genomic DNA using primers oVL129 and oVL67. Fragments were assembled by Gibson Assembly® into pVL15, linearized with KpnI		***qed1*-2 + UBQ::At_PPR9**
**pVL45**	HPL::At_PPR10	*A. thaliana-T. cacao* QED1 chimera constructed based on PPR annotation from Cheng *et al.*, 2016: *RbcS* transit peptide, 3x glycine linker, *T. cacao* PPR tract from first motif to motif 9, *A. thaliana* PPR motif 10, *T. cacao* PPR tract from motif 11 to 15 and C-terminus expressed from the *HPL* promoter	*At_PPR10*	*HPL*			*P. sativum RbcS* transit peptide-encoding sequence was amplified from pIT42 using primers oVL80 and otHA6 (including 3x glycine linker); *T. cacao QED1* PPR tract (motif 1-9) was amplified from *T. cacao* genomic DNA using primers Tc-09 and oVL134, *A. thaliana* PPR motifs 10 was amplified from *A. thaliana* Col-0 genomic DNA using primers oVL135 and oVL132, *T. cacao* PPR motifs 11 to 15 and C-terminus were amplified from *T. cacao* genomic DNA using primers oVL133 and oVL67. Fragments were assembled by Gibson Assembly® into pVL14, linearized with KpnI		***qed1*-2 + HPL::At_PPR10**
**pVL46**	UBQ::At_PPR10	*A. thaliana-T. cacao* QED1 chimera constructed based on PPR annotation from Cheng *et al.*, 2016: *RbcS* transit peptide, 3x glycine linker, *T. cacao* PPR tract from first motif to motif 9, *A. thaliana* PPR motif 10, *T. cacao* PPR tract from motif 11 to 15 and C-terminus expressed from the *UBQ* promoter		*UBQ*			*P. sativum RbcS* transit peptide-encoding sequence was amplified from pIT42 using primers oVL80 and otHA6 (including 3x glycine linker); *T. cacao QED1* PPR tract (motif 1-9) was amplified from *T. cacao* genomic DNA using primers Tc-09 and oVL134, *A. thaliana* PPR motifs 10 was amplified from *A. thaliana* Col-0 genomic DNA using primers oVL135 and oVL132, *T. cacao* PPR motifs 11 to 15 and C-terminus were amplified from *T. cacao* genomic DNA using primers oVL133 and oVL67. Fragments were assembled by Gibson Assembly® into pVL15, linearized with KpnI		***qed1*-2 + UBQ::At_PPR10**

Construct	Construct name used in this work	Description	Transgene	Transgene promoter	Bacterial resistance	Plant resistance	Cloning strategy	Target for plant transformation	Name of transgenic lines
**pVL47**	HPL::At_PPR11	*A. thaliana-T. cacao* QED1 chimera constructed based on PPR annotation from Cheng *et al.*, 2016: *RbcS* transit peptide, 3x glycine linker, *T. cacao* PPR tract from first motif to motif 10, *A. thaliana* PPR motif 11, *T. cacao* PPR tract from motif 12 to 15 and C-terminus expressed from the *HPL* promoter	*At_PPR11*	*HPL*	*nptII* (kanamycin)	*pat* (BASTA)	*P. sativum RbcS* transit peptide-encoding sequence was amplified from pIT42 using primers oVL80 and otHA6 (including 3x glycine linker); *T. cacao QED1* PPR tract (motif 1-10) was amplified from *T. cacao* genomic DNA using primers Tc-09 and oVL130, *A. thaliana* PPR motifs 11 was amplified from *A. thaliana* Col-0 genomic DNA using primers oVL131 and oVL126, *T. cacao* PPR motifs 11 to 15 and C-terminus were amplified from *T. cacao* genomic DNA using primers oVL127 and oVL67. Fragments were assembled by Gibson Assembly® into pVL14, linearized with KpnI	*qed1*-2 (Wagoner *et al.*, 2015)	***qed1*-2 + HPL::At_PPR11**
**pVL48**	UBQ::At_PPR12	*A. thaliana-T. cacao* QED1 chimera constructed based on PPR annotation from Cheng *et al.*, 2016: *RbcS* transit peptide, 3x glycine linker, *T. cacao* PPR tract from first motif to motif 10, *A. thaliana* PPR motif 11, *T. cacao* PPR tract from motif 12 to 15 and C-terminus expressed from the *UBQ* promoter		*UBQ*			*P. sativum RbcS* transit peptide-encoding sequence was amplified from pIT42 using primers oVL80 and otHA6 (including 3x glycine linker); *T. cacao QED1* PPR tract (motif 1-10) was amplified from *T. cacao* genomic DNA using primers Tc-09 and oVL130, *A. thaliana* PPR motifs 11 was amplified from *A. thaliana* Col-0 genomic DNA using primers oVL131 and oVL126, *T. cacao* PPR motifs 11 to 15 and C-terminus were amplified from *T. cacao* genomic DNA using primers oVL127 and oVL67. Fragments were assembled by Gibson Assembly® into pVL15, linearized with KpnI		***qed1*-2 + UBQ::At_PR11**
**Previously generated transgenic lines used in this work**									
**pRB58**	pRB58	Transplastomic tobacco line described in Bock *et al.* (1996)	-	-	-	*aadA* (spectinomycin)	-	-	**pRB58**
**Δ*ycf3***	Δ*ycf3*	Transplastomic tobacco line described in Ruf *et al.* (1997)	-	-	-		-	-	**Δ*ycf3***
**Δ*atpB***	Δ*atpB*	Transplastomic tobacco line generated by Martin Hager (University of Freiburg)	-	P*rrn*	-		The region of the tobacco plastome corresponding to nucleotide positions 52383-58539 (NC_001879.2) was excised from a SalI ptDNA clone (provided by P. Maliga, Piscataway, NJ) as a HindIII/KpnI fragment and ligated into a HindIII/KpnI digested pUC19 vector (resulting in plasmid p*atpB*). Subsequently, this plasmid was digested with HpaI/SmaI to remove the nearly complete coding sequence of *atpB* and part of *atpE.* The digested plasmid was dephosphorylated and the *aadA* expression cassette, isolated as a Ecl136II/DraI fragment from plasmid pZS195 (Svab and Maliga, 1993), was inserted, resulting in the final transformation vector Δ*atpB*	*N. tabacum*	**Δ*atpB***
**WX7**	-	Spontaneous mutant isolated by Michael Tillich (LMU Munich)	-	-	-	-	Tobacco mutant isolated during an unrelated plastid transformation. The mutant regenerated on spectinomycin-containing RMPO plates but did not contain the *aadA* resistance cassette. A point mutation in *rrn16* was discovered by bulk sequencing	-	**WX7**

Construct	Construct name used in this work	Description	Transgene	Transgene promoter	Bacterial resistance	Plant resistance	Cloning strategy	Target for plant transformation	Name of transgenic lines
**Vector backbones used in this work**									
**pORE-E2**	-	Vector used for cloning (Coutu *et al*., 2007)	-	*HPL*	*nptII* (kanamycin)	*nptII* (kanamycin)	-	-	-
**pORE-E3**	-	Vector used for cloning (Coutu *et al*., 2007)	-	*ENTCUP2*		*pat* (BASTA)	-	-	-
**pIT42**	-	Vector used for amplifying *RbcS* transit peptide (Tabatabaei *et al.*, 2019)	-	-		-	-	-	-
**pVL14**	-	Modified pORE-E2 with *pat* gene for plant resistance	-	*HPL*		*pat* (BASTA)	*pat* gene from pORE-E3 cloned as FseI/AscI fragment in pORE-E2	-	-
**pVL15**	-	Modified pVL14 with UBQ promoter for transgene expression	-	*UBQ*			The *UBIQUITIN 10* promoter was amplified from *Arabidopsis* Col-0 genomic DNA using primers P_UBQ10for and P_UBQ10rev and cloned as a XhoI/EcoRI fragment in pVL14	-	-
**pVL22**	-	Modified pORE-E2 with UBQ promoter for transgene expression	-	*UBQ*		*nptII* (kanamycin)	The *UBIQUITIN 10* promoter was amplified from *Arabidopsis* Col-0 genomic DNA using primers P_UBQ10for and P_UBQ10rev and cloned as a XhoI/EcoRI fragment in pORE-E2	-	-

Supplementary Table S12. List of oligonucleotides used in this study. Uppercase: gene-specific sequence; Lowercase: overlapping region or restriction enzyme cut site used for cloning; Italics: glycine linker; Underlined: T7 promoter sequence.

Primer name	Sequence (5'->3')	Purpose
Oligo-dT	$(T)_{18}$	cDNA synthesis
Random hexamer	provided in QuantiTect Reverse Transcription Kit (Qiagen)	
MART-1tg5	TTTGAGCTGTGGATAGAATCGGACATTTGGCGATAAGCTTGC	cDNA synthesis for "Tobacco-editotype" iPLEX/MassARRAY® assay
MART-2tg7	ACTACAAGTCATGCCCTTGGATACACCAAGCACTACACTTAG	
MART-3tg8	CATGAATCCCAATAACGTCACCTGAATTTCGAATAGTATTCAAG	
MART-4tg9	AGGAGATTACGGATTCCGACATTCGCGAGATCATGCACCTC	
MART-5tg12	ATTGTTACGATGTGGTTCTGCTATTCGAGATGTGAGTAAAACTC	
MART-6tg91	CTTGTGTGACGGATAACACCGAACAAGGTGATCACCAAAAGG	
MART-7tg89	GGATACCAGAGATACGGATCCTGCGCTTCCACTATATCAAC	
MART-8tg88	CTTCCTACAGCACATGTACCCTGCGCTTCCACTATATCAAC	
MART-9tg87	AACGAACCTCAACGACACACGTAGTCTTTTACAGTGAAAGAAG	
MART-10tg62	AAATCTCTTTGTAGAGCCGACATAATAGGTAGGAGCATAAACTG	
MART-11tg61	TGCAGAATTGGCACGAATCGTTTCTTGAAGCTCAATCTCTCC	
MART-12+13tg65	AGCATCATCACGTATTTGCTGGAAAGAAAAGCAACGACTGGAG	
MART-14+15tg63	CGTCCCTTCATTAGCATTAACGAGGTAGGAATTTCTCGAACG	
MART-16+17tg58	TGTTGGGAGACAGAACGAAGCTTCGTATACGTCAGGAGTCC	
MART-18tg54	TCAGCCATCGATCAAACCTGTCCAGAAGAAGATGCCATTCG	
MART-19tg57	GAAATCCCAACCTGATTGTTCTCCAGAAGAAGATGCCATTCG	
MART-20tg55	CAGGATTTGCTTGTCACTCCTCCAGAAGAAGATGCCATTCG	
MART-21tg51	TAATGTGTGCCAGGACTAATGCCTGAGCAATTGCAATAATTGG	
MART-22tg68	CATCATATTGACAAGCAGGTCGCTCATTACTAAACTTGTTGAAG	
MART-23tg35	GTTGAAACCATCACTCCAATCTGCCAAAATAGGAACAAGGATAG	
MART-24tg75	GGTAACTTATGCCGGAACACATAGCTTGTATCCATAGAACATC	
MART-25tg94	CAGAAGATAGTTGCGGATGCATTAAGGAACCCGCAAATATTG	
MART-26tg83	ACCCGTTCGTAAATTGCAATGAAAAAGTAAAAGGTCCCGAGAC	
MART-27tg82	AACGCCCACCCTAAATTCTCCTAAAACCACAGGATATGACTG	
MART-28tg79	CTACAAGGTCTCTTGTTTAGGCATGTGTGTAGGGGGATAATG	
MART-29tg76	ACGCTAGCAATCAGTACGAGTTTTTTAAATTCCGTTGGCCAAG	
MART-30tg73	GTATTTGCTTGGAACGCGAGAAATAAGAACCATAATTCCAACC	
MART-31tg74	CTTGTTCAAGGGTTGTGGACGTAACTAATCCCAACATGGAAG	
MART-32tg85	AATGCCTTCTTTCTATCCAGGTCCAAATAATCCCGTACCATG	
MART-33tg86	TCTTTACGCTTAAGACGCATGCCTAGACCCCCTAATATAAGAC	
MART-34tg43	CTTTCAGAGAGAGTCGTAGGGCCCAGAAATACCTTGTTTACG	
MART-35tg36	TTCGTAGTCATGAACGGGTCAGATTGGGTCCCAGCGGATC	
MART-36tg40	TTGGAATCCGTAAGACGTGGCCTAAAACGATCTACTAAATTCATC	
MART-37tg39	TCAGAATGGAATGGTAGAACGGATGAACTGCATTGCTGATATTG	
MART-38tg37	AAATGGCTGTTGCTAATGCTCCAATTCAACATTTTGTTCGTTCG	
MART-39tg41	TTCCTATAAGGGTGGGCATCCAATTCAACATTTTGTTCGTTCG	
MART-40tg42	AGCGGTTGACGTTTGATACGTCGAAATCATATAAAGACAATTCC	
MART-41tg49	TGGAACACCGACTGACTATCGACCCAATGCTTTATTTCTGTC	
MART-42tg47	CATGATAGTCCAGTTTACGAGAGTGGAGACAGAATAAAGCGTC	
MART-43tg48	GTACCGGAGACATTAACACGTGGTAGAATATTCATGTGGGAC	
MART-44tg44	ATAATGAGGCTGTCTCGTCGGGCAATTATAGATCCTGGAAGG	
MART-45tg45	TTGGTAGGATAGCCCTTAATCTGGTAGAATATTCATGTGGGAC	
MART-46tg26	AGTACATATTCGGTGCCCAGCACAATTCTATATATTCCATTGAC	
MART-47tg27	CGCGATAACACAGAACTGACTCTCTTAGATTCAAACCCATAGC	
MART-48tg25	TCCAGTCGTTTACTTAGGAAGTACTTACACGAGCCCATATCC	
MART-49tg24	TAGGATGAGGAGGTTATTAGCTCTCTTAGATTCAAACCCATAGC	
MART-50tg22	CCATTGATACACGAGCTTCCAACGCTCGCTAATAAGTACTGC	
MART-51tg20	CTTTGAACCCTACAGCTTGGCCCAAGCACTTATTTGTTGAGG	
MART-52tg19	GTGTGACCTTGTACTCTTCCCTCGCAACAATCCAATAAGTTC	

MART-53tg70	GGAAACCTTGGTCTAACGAGCCTCTTACTAAGACTACAGAATG	cDNA synthesis for "Tobacco-editotype" iPLEX/MassARRAY® assay
MART-54tg69	ACCCTGTTTAGGCTCACATCATTTTACATATCCATACGATTGAG	
MART-55tg30	CTACGCACAGAGATCGAAACACGACGGAACCTTGCTTATTTC	
MART-56tg31	AGTTCTCCATGAGGTCTACGAGGCGTGTAGGTGCACTATTC	
MART-57tg29	AGTGGAAAGCTCTGTAGTCGAGGCGTGTAGGTGCACTATTC	
MART-58tg1	CTTATGTATGTACCGTGTCTCGTGTTTCCTTGTTCTGGGATC	
MART-59tg16	GGATTCTATATGCTCGACTAGAAAAAACGAGCAGTTCTAGTAAG	
MART-60tg17	GCAGTGCGGTTAAAGACTAGCACCGGGCCCTTATTGCAGC	
MART-61tg15	GGTAATGTTACGTGTGCTCGCACCGGGCCCTTATTGCAGC	
MART-62tg93	TAAGTTCACGAATACGGCTAGTTCAATCATAAACAATACTTCGC	
MART-63tg50	TAGCCGTCAACGCTTTAGTCTCTTGATGAGATCGATATTGATG	
MART-64tg32	ATAACCATGCGTTCCCTTGGCACCGCTGCTCAAGACTTTAG	
MART-65tg33	GTTTAAGAATCGTGCGGGAGATTACGTGCGACTATCTCCAC	
MART-PCR1	ACGTTGGATGTGCTCCTTATACAGGAGCAG	PCR primers for "Tobacco-editotype" iPLEX/MassARRAY® assay
MART-PCR2	ACGTTGGATGTTTGAGCTGTGGATAGAATCG	
MART-PCR3	ACGTTGGATGACTACAAGTCATGCCCTTGG	
MART-PCR4	ACGTTGGATGCATGAATCCCAATAACGTCAC	
MART-PCR5	ACGTTGGATGAGGAGATTACGGATTCCGAC	
MART-PCR6	ACGTTGGATGGGGAGTTTCGGGTTTAATAC	
MART-PCR7	ACGTTGGATGATTGTTACGATGTGGTTCTGC	
MART-PCR8	ACGTTGGATGCCATCTACAGGAGATACTCG	
MART-PCR9	ACGTTGGATGGGATACCAGAGATACGGATC	
MART-PCR10	ACGTTGGATGCTGAGGAGCCGTATGAGATG	
MART-PCR11	ACGTTGGATGAACGAACCTCAACGACACAC	
MART-PCR12	ACGTTGGATGAAATCTCTTTGTAGAGCCGAC	
MART-PCR13	ACGTTGGATGTGCAGAATTGGCACGAATCG	
MART-PCR14	ACGTTGGATGGTGGGGCAAGCTCTTCTATT	
MART-PCR15	ACGTTGGATGAGCATCATCACGTATTTGCTG	
MART-PCR16	ACGTTGGATGGGCAAGCTCTTCTATTCTGG	
MART-PCR17	ACGTTGGATGCGTCCCTTCATTAGCATTAAC	
MART-PCR18	ACGTTGGATGCAAATGTATAACTCCCCAGG	
MART-PCR19	ACGTTGGATGCACTCCAGTCGTTGCTTTTC	
MART-PCR20	ACGTTGGATGTCAGCCATCGATCAAACCTG	
MART-PCR21	ACGTTGGATGGACTCCTGACGTATACGAAG	
MART-PCR22	ACGTTGGATGGAAATCCCAACCTGATTGTTC	
MART-PCR23	ACGTTGGATGATCCTCTTTTCGACTCTGAC	
MART-PCR24	ACGTTGGATGCAGGATTTGCTTGTCACTCC	
MART-PCR25	ACGTTGGATGTAATGTGTGCCAGGACTAATG	
MART-PCR26	ACGTTGGATGCATCATATTGACAAGCAGGTC	
MART-PCR27	ACGTTGGATGCATTTGCTTCTCTTCGATGG	
MART-PCR28	ACGTTGGATGGTTGAAACCATCACTCCAATC	
MART-PCR29	ACGTTGGATGGGTAACTTATGCCGGAACAC	
MART-PCR30	ACGTTGGATGGGAATAATTACCGGCCAAAA	
MART-PCR31	ACGTTGGATGCAGAAGATAGTTGCGGATGC	
MART-PCR32	ACGTTGGATGTAGCTACTTTAGCGGCTTGG	
MART-PCR33	ACGTTGGATGTTTATTTTGTACACGGCGGG	
MART-PCR34	ACGTTGGATGCTACAAGGTCTCTTGTTTAGG	
MART-PCR35	ACGTTGGATGACGCTAGCAATCAGTACGAG	
MART-PCR36	ACGTTGGATGGTATTTGCTTGGAACGCGAG	
MART-PCR37	ACGTTGGATGCTTGTTCAAGGGTTGTGGAC	
MART-PCR38	ACGTTGGATGTCATCTTTGGACCGTAGGAG	
MART-PCR39	ACGTTGGATGTCTTTACGCTTAAGACGCATG	
MART-PCR40	ACGTTGGATGGCTAGTGTGGGACAATCTAC	

MART-PCR41	ACGTTGGATGTTCGTAGTCATGAACGGGTC	PCR primers for "Tobacco-editotype" iPLEX/MassARRAY® assay
MART-PCR42	ACGTTGGATGTGCCGTTAGTAGGCCTAGTC	
MART-PCR43	ACGTTGGATGTTGGAATCCGTAAGACGTGG	
MART-PCR44	ACGTTGGATGCGTTTTAGGAGGCCCTAATG	
MART-PCR45	ACGTTGGATGCCTACCGTCTTTTTTTTGGG	
MART-PCR46	ACGTTGGATGATTCCATTAATAACTGGCCG	
MART-PCR47	ACGTTGGATGTTCCTATAAGGGTGGGCATC	
MART-PCR48	ACGTTGGATGAGCGGTTGACGTTTGATACG	
MART-PCR49	ACGTTGGATGGGATGGAATCAAATATGCAG	
MART-PCR50	ACGTTGGATGTGGAACACCGACTGACTATC	
MART-PCR51	ACGTTGGATGCATGATAGTCCAGTTTACGAG	
MART-PCR52	ACGTTGGATGACTACAGTGGAAGTGTGTTG	
MART-PCR53	ACGTTGGATGCACACGGTTCCTTTATCCCC	
MART-PCR54	ACGTTGGATGTTGGTAGGATAGCCCTTAATC	
MART-PCR55	ACGTTGGATGAGTACATATTCGGTGCCCAG	
MART-PCR56	ACGTTGGATGTCCTCTAATGAATTCCCTGG	
MART-PCR57	ACGTTGGATGCGCGATAACACAGAACTGAC	
MART-PCR58	ACGTTGGATGTCCAGTCGTTTACTTAGGAAG	
MART-PCR59	ACGTTGGATGGGGAGGAAGATCAGAATTAG	
MART-PCR60	ACGTTGGATGCCATTGATACACGAGCTTCC	
MART-PCR61	ACGTTGGATGCTTTGAACCCTACAGCTTGG	
MART-PCR62	ACGTTGGATGGGGTGCAGATTCATAATAGG	
MART-PCR63	ACGTTGGATGGGAAACCTTGGTCTAACGAG	
MART-PCR64	ACGTTGGATGACCCTGTTTAGGCTCACATC	
MART-PCR65	ACGTTGGATGCTACGCACAGAGATCGAAAC	
MART-PCR66	ACGTTGGATGAGTTCTCCATGAGGTCTACG	
MART-PCR67	ACGTTGGATGTTCAGAGGGAGAAGAAGAGG	
MART-PCR68	ACGTTGGATGCATTCGATTCGTCGATCCTC	
MART-PCR69	ACGTTGGATGCTTATGTATGTACCGTGTCTC	
MART-PCR70	ACGTTGGATGGGATTCTATATGCTCGACTAG	
MART-PCR71	ACGTTGGATGGCAGTGCGGTTAAAGACTAG	
MART-PCR72	ACGTTGGATGTGCAAAGCGTAAGGGTATTC	
MART-PCR73	ACGTTGGATGTTTTGACGCAGCAAGTAGGG	
MART-PCR74	ACGTTGGATGGCAACCGTATCCACTTTTTG	
MART-PCR75	ACGTTGGATGTAGCCGTCAACGCTTTAGTC	
MART-PCR76	ACGTTGGATGATAACCATGCGTTCCCTTGG	
MART-PCR77	ACGTTGGATGCGATGCATATGTAGAAAGCC	
MART-PCR78	ACGTTGGATGTTTGAGCTGTGGATAGAATCG	
MART-PCR79	ACGTTGGATGTGCTCCTTATACAGGAGCAG	
MART-PCR80	ACGTTGGATGCTTTCGTTTCTTTGGGCCAC	
MART-PCR81	ACGTTGGATGGGGAGTTTCGGGTTTAATAC	
MART-PCR82	ACGTTGGATGAGGAGATTACGGATTCCGAC	
MART-PCR83	ACGTTGGATGCCAAGGTAAAAGAGCTCCTG	
MART-PCR84	ACGTTGGATGCTTGTGTGACGGATAACACC	
MART-PCR85	ACGTTGGATGTTTTTAGGTGGTCTACGGGC	
MART-PCR86	ACGTTGGATGCTTCCTACAGCACATGTACC	
MART-PCR87	ACGTTGGATGCTTTACCGAGGCTGAGAATG	
MART-PCR88	ACGTTGGATGTGGCTATAACAGAGTTTCTC	
MART-PCR89	ACGTTGGATGGATGTACGGTCTAATGAGGC	
MART-PCR90	ACGTTGGATGAGCATCATCACGTATTTGCTG	
MART-PCR91	ACGTTGGATGGTGGGGCAAGCTCTTCTATT	
MART-PCR92	ACGTTGGATGCGTCCCTTCATTAGCATTAAC	
MART-PCR93	ACGTTGGATGTGTTGGGAGACAGAACGAAG	
MART-PCR94	ACGTTGGATGTCAGCCATCGATCAAACCTG	

MART-PCR95	ACGTTGGATGCACTCCAGTCGTTGCTTTTC	
MART-PCR96	ACGTTGGATGGAAATCCCAACCTGATTGTTC	
MART-PCR97	ACGTTGGATGGACTCCTGACGTATACGAAG	
MART-PCR98	ACGTTGGATGCAGGATTTGCTTGTCACTCC	
MART-PCR99	ACGTTGGATGATCCTCTTTTCGACTCTGAC	
MART-PCR100	ACGTTGGATGCCTCACGTGCGAAATTATAG	
MART-PCR101	ACGTTGGATGCATTTGCTTCTCTTCGATGG	
MART-PCR102	ACGTTGGATGCATCATATTGACAAGCAGGTC	
MART-PCR103	ACGTTGGATGGGAGTGCGTTTATGTTTCTG	
MART-PCR104	ACGTTGGATGGGAATAATTACCGGCCAAAA	
MART-PCR105	ACGTTGGATGGGTAACTTATGCCGGAACAC	
MART-PCR106	ACGTTGGATGTTACCGACAAACCTGTGCTC	
MART-PCR107	ACGTTGGATGACCCGTTCGTAAATTGCAATG	
MART-PCR108	ACGTTGGATGAACGCCCACCCTAAATTCTC	
MART-PCR109	ACGTTGGATGTAATCAGTCATATCCTGTGG	
MART-PCR110	ACGTTGGATGGGAATTATTACCTCATGCCC	
MART-PCR111	ACGTTGGATGATGTATGGTCTTGGATCATC	
MART-PCR112	ACGTTGGATGTTGTTAAGCATAGTCATGG	
MART-PCR113	ACGTTGGATGAATGCCTTCTTTCTATCCAGG	
MART-PCR114	ACGTTGGATGGGATTTATCTGAACCAATAC	
MART-PCR115	ACGTTGGATGCTTTCAGAGAGAGTCGTAGG	
MART-PCR116	ACGTTGGATGTGCCGTTAGTAGGCCTAGTC	
MART-PCR117	ACGTTGGATGTTCGTAGTCATGAACGGGTC	
MART-PCR118	ACGTTGGATGCCGGTTTAGCTTACGATGTG	
MART-PCR119	ACGTTGGATGTCAGAATGGAATGGTAGAACG	
MART-PCR120	ACGTTGGATGAAATGGCTGTTGCTAATGCTC	
MART-PCR121	ACGTTGGATGTTCCTATAAGGGTGGGCATC	PCR primers for "Tobacco-editotype" iPLEX/MassARRAY® assay
MART-PCR122	ACGTTGGATGATTCCATTAATAACTGGCCG	
MART-PCR123	ACGTTGGATGGCGAAAGGGGAGTATCCTAT	
MART-PCR124	ACGTTGGATGTGGAACACCGACTGACTATC	
MART-PCR125	ACGTTGGATGGGATGGAATCAAATATGCAG	
MART-PCR126	ACGTTGGATGGGTTCGAGAGAAAGTAACAG	
MART-PCR127	ACGTTGGATGGTACCGGAGACATTAACACG	
MART-PCR128	ACGTTGGATGATAATGAGGCTGTCTCGTCG	
MART-PCR129	ACGTTGGATGCCACTTATGAAAGGCCAAGC	
MART-PCR130	ACGTTGGATGGTATCCGCGGGATTAATTTG	
MART-PCR131	ACGTTGGATGCGCGATAACACAGAACTGAC	
MART-PCR132	ACGTTGGATGTCCTCTAATGAATTCCCTGG	
MART-PCR133	ACGTTGGATGGGACCATAACGGAATTTCGG	
MART-PCR134	ACGTTGGATGTAGGATGAGGAGGTTATTAGC	
MART-PCR135	ACGTTGGATGGGGACAAATTTTAGCGGATG	
MART-PCR136	ACGTTGGATGTATAAACATCAACAGCTCCG	
MART-PCR137	ACGTTGGATGGTGTGACCTTGTACTCTTCC	
MART-PCR138	ACGTTGGATGCTGCCTTACGTAAAGTTGCC	
MART-PCR139	ACGTTGGATGTTTTAGAACAGGTCCGAGTC	
MART-PCR140	ACGTTGGATGGTTTGATTCAGAGGGAGAAG	
MART-PCR141	ACGTTGGATGTTCAGAGGGAGAAGAAGAGG	
MART-PCR142	ACGTTGGATGAGTTCTCCATGAGGTCTACG	
MART-PCR143	ACGTTGGATGAGTGGAAAGCTCTGTAGTCG	
MART-PCR144	ACGTTGGATGATCCATCGTCTGTAGGATTC	
MART-PCR145	ACGTTGGATGGGAGGCAGGAGTTCATTTTG	
MART-PCR146	ACGTTGGATGTGCAAAGCGTAAGGGTATTC	
MART-PCR147	ACGTTGGATGGCAGTGCGGTTAAAGACTAG	
MART-PCR148	ACGTTGGATGGGTAATGTTACGTGTGCTCG	

MART-PCR149	ACGTTGGATGTAAGTTCACGAATACGGCTAG	PCR primers for "Tobacco-editotype" iPLEX/MassARRAY® assay
MART-PCR150	ACGTTGGATGGCCCATTCATCCTAGTTAGC	
MART-PCR151	ACGTTGGATGGCGTACTTATGTCATGATGG	
MART-PCR152	ACGTTGGATGGTTTAAGAATCGTGCGGGAG	
MART-E1	CGTACCATGACGTATCT	Extension primers for "Tobacco-editotype" iPLEX/MassARRAY® assay
MART-E2	AAAGGCGTTTACTGTCT	
MART-E3	ATTGATGAAAAGGGGCT	
MART-E4	TGGAAAGGGAGTGTGTG	
MART-E5	AAGCTCTTCTATTCTGGTT	
MART-E6	TTCTCTTGGCTATATGGTT	
MART-E7	CTTATTGCAGCCCACTCTACT	
MART-E8	CCGATATTTTAGCAACAAATC	
MART-E9	GGATAGGTTCGATCTATAGTC	
MART-E10	AATATTCATGTGGGACTTTCTCA	
MART-E11	CTAATTCTTCTTCTGATGATCGATT	
MART-E12	TCCATTAATAACTGGCCGTTTTGAT	
MART-E13	TCAAACCCATAGCTGAT	
MART-E14	CGAAAATTCGAGTGGCT	
MART-E15	AGATTGGGGAGGAAGAT	
MART-E16	ATGAATTCCCTGGGAACTT	
MART-E17	TCCACTATATCAACTGTACTT	
MART-E18	GCTGATATTGAACCCAAAAAA	
MART-E19	TATCATTCGATTCGTCGATCCT	
MART-E20	TGTAGGTGCACTATTCCGTGGT	
MART-E21	TCGACCCACTTACTTCTATTATGT	
MART-E22	TTGGCGATAAGCTTGCGCTTGTTT	
MART-E23	TTGTTCGTTCGGGTTTGATTGTGTC	
MART-E24	GATGCCATTCGTTTGAT	
MART-E25	ATGTATCACACGTGTAAAAT	
MART-E26	GTTATAACGGAAAAGGGTAC	
MART-E27	ACCCACTTACTTCTATTATGT	
MART-E28	AAGCGTAAGGGTATTCATATTA	
MART-E29	AACCCAGGGCCCATATTCTTCGC	
MART-E30	GTGCGACTATCTCCACTATAGAAA	
MART-E31	CGTGTAGGTGCACTATTCCGTGGT	
MART-E32	GGCCCTTATTGCAGCCCACTCTACT	
MART-E33	GTATACGTCAGGAGTCCATTGATGA	
MART-E34	ATCGAACCTATCCAATTTTTACAGTA	
MART-E35	CAATCTCTCCCCCGGAT	
MART-E36	AGATGCCATTCGTTTGAT	
MART-E37	GTCAGGAGTCCATTGATGA	
MART-E38	TCTATTATTGACAACCTCGT	
MART-E39	TATTGCTTTTGCTGTCAAAT	
MART-E40	TCAATATGTATGGTCTTGGAT	
MART-E41	TTAATAACTATCTTTGTAGCCC	
MART-E42	TGTTACTTCGAAAGTAGCTGCTT	
MART-E43	GTATGTGTGATAGCATCTACTATAC	
MART-E44	AAAAGCACCGAAGTAATGTCTAAACC	
MART-E45	GGAGTGTTAAGTGATTTATTAGATAA	
MART-E46	TTGCAATGGCTTCTTTAT	
MART-E47	AAGGTCCCGAGACGAAAAT	
MART-E48	TCTTTTAGTCTTTCTAGGGT	
MART-E49	GCCGTGGGAGGGTTACAATT	

MART-E50	CCAAGTGTATCTTGTCTTTACTA	
MART-E51	AATACAAATAATCTATGCAGCTT	
MART-E52	GAAGAAGATGCCATTCGTTTGAT	
MART-E53	CCAATGCTTTATTTCTGTCCTAGTT	
MART-E54	TCTGTTACTTCGAAAGTAGCTGCTT	
MART-E55	CAGAAATACCTTGTTTACGTATCATT	
MART-E56	ACAGCTCCGAATTGGAT	
MART-E57	CCGGTATTGGCCATAATT	
MART-E58	TCCAATAAGTTCTCCGGGT	
MART-E59	ATGATTTTTTTTGGGCATTT	
MART-E60	GGAAGTGTGTTGAATCAAGAA	
MART-E61	CACAGGATATGACTGATTAACT	
MART-E62	CTTTTTACACTATTTCGGAAACC	
MART-E63	AATACTTCGCTAGAAAATTTGAAA	
MART-E64	TCAATATCTCTACTATCTAACAGTT	
MART-E65	CACACTTTAATCATTTATGATGATC	
MART-E66	TGGAATGATATTAACTCCTATTTATT	
MART-E67	TCCTGGAAGGCAATTCT	
MART-E68	CAGGAGTCCATTGATGA	Extension primers for "Tobacco-editotype" iPLEX/MassARRAY® assay
MART-E69	TCCATAGAACATCTGGCGT	
MART-E70	GTTCGGGTTTGATTGTGTC	
MART-E71	AGCGTAAGGGTATTCATATTA	
MART-E72	TTACTAAACTTGTTGAAGAGAT	
MART-E73	AAATACTTGCACAAATAGCTATAT	
MART-E74	AGGAATTAACTTATGTAATAGAGT	
MART-E75	CTAAACAATCTTATTTTTTTGAACAT	
MART-E76	GTGGGGCAAGCTCTTCTATTCTGGTT	
MART-E77	TTCGATTCGTCGATCCT	
MART-E78	CTTCGAAAGTAGCTGCTT	
MART-E79	AGTTTTTGACGCAGCAAG	
MART-E80	TAATAACTGGCCGTTTTGAT	
MART-E81	TGCTGATATTGAACCCAAAAAA	
MART-E82	ATTGGGGAAAAATCAATATACTT	
MART-E83	CTCTAATGAATTCCCTGGGAACTT	
MART-E84	AAGGTGATCACCAAAAGGAATTACT	
MART-E85	AGGAATATCGAAAATTCGAGTGGCT	
MART-E86	AAGATTGTCACTATCATCAATATCGT	
MA17	ACTACAAGTCATGCCCTTGGAACTCACTCTTGTTGATAAAGGC	
MA18	AGCTTTACACCAGTGTTGTTGGTAACTCGTTGTTCAAACAACTA	
MA19	TTTGAGCTGTGGATAGAATCGGTAACTCGTTGTTCAAACAACTA	
MA20	TGTTGGGAGACAGAACGAAGCTCCCGAAAGGATTCCTATGG	
MA21	CAGGATTTGCTTGTCACTCCGTCGCTAGTATAGTTCCCTAGCC	
MA22	CATGATAGTCCAGTTTACGAGCCCCTAGAGTAGCTGTTAATACG	
MA23	ACCCTCTATGTAGTCAGATTGATTTATCAATATTAGAATCTAG	
MA24	CTTGTGTGACGGATAACACCCAATATGAACAACAATTCAACCG	cDNA synthesis for "Tobacco-OFF-targets" iPLEX/MassARRAY®
MA25	ATAACCATGCGTTCCCTTGGAAACATTTATCTATCCAGCCATA	
MA26	CTACAAGGTCTCTTGTTTAGGGGTTGAAAGAAAAGAATCTTTCG	
MA27	AGTTCTCCATGAGGTCTACGCATACCTTTAGCCAATTTAGCTC	
MA28	AAATGGCTGTTGCTAATGCTCTTCCCATGTGGCAGGCATATATC	
MA29	GATAACATGGAACCTTTCGACGTCCGGCCCTCCTCAGTATCACC	
MA30	GACTGGTTATTGAGAGCTTAGATAGCGTACTTAGTACTGGTGCC	
MA31	GGAAACCTTGGTCTAACGAGCTCCACATTGGATCAAGAACAGG	
MA32	CATCATATTGACAAGCAGGTCAGAGTATATCTTTCCACGGATCG	

MA33	TATCGCTCCCTGTTTGTTGCTTCGAGTTCCTTTTGGATTTCCTTC	cDNA synthesis for "Tobacco-OFF-targets" iPLEX/MassARRAY®
MA34	TAGCCGTCAACGCTTTAGTCTGTGTGAAAATTGCATAATACTGG	
MA35	ACGCTAGCAATCAGTACGAGTTCCTTAATTGAGTATAGAACGG	
MA36	ATTGTTACGATGTGGTTCTGCGGGTTTAGACATTACTTCGGTGC	
MA37	TGCAGAATTGGCACGAATCGTAGAACGACCCTCACAAATTGCG	
MA38	TTCCTATAAGGGTGGGCATCTAGATTATTAGCCCCGGGACACC	
MA39	ATAATGAGGCTGTCTCGTCGATGAATTATTCATTAGAATCGCC	
MA40	GAAATCCCAACCTGATTGTTCTTCGAATCCTTCCGTCCCAGAAC	
MA41	GGATACCAGAGATACGGATCATGGAAGCTGAACTTCCCTTTGG	
MA42	CTTGTTCAAGGGTTGTGGACTTGGCAGTAAAAATCACTACACG	
MA43	CAGAGTCAGTGTATTGAAGAGGATCGATTCTTTGGGATCCTTCC	
MA44	CGATTAGCGTGCTCCAAATGAGGTTTTCTTCTTGATGAGATCG	
MA45	AGTGGAAAGCTCTGTAGTCGCCCTTTGACATCTCTTCATCTGC	
MA46	TCCAGTCGTTTACTTAGGAAGATTCTTCGATTTCTTCCGGAAAC	
40	ACGTTGGATGACTACAAGTCATGCCCTTGG	PCR primers for "Tobacco-OFF-targets" iPLEX/MassARRAY®
41	ACGTTGGATGTTGTTCAATAGCCCCTCCAC	
42	ACGTTGGATGAGTTGTTCAATAGCCCCTCC	
43	ACGTTGGATGTGTTGGGAGACAGAACGAAG	
44	ACGTTGGATGGAATGCTTGGTACTTTGTAG	
45	ACGTTGGATGCATGATAGTCCAGTTTACGAG	
46	ACGTTGGATGACCCTCTATGTAGTCAGATTG	
47	ACGTTGGATGCTTGTGTGACGGATAACACC	
48	ACGTTGGATGATAACCATGCGTTCCCTTGG	
49	ACGTTGGATGGAGTATGAGTGTGTGACTTG	
50	ACGTTGGATGCATGGATTTGGATATTTCCC	
51	ACGTTGGATGTGGACTCGACTGGAGTAATG	
52	ACGTTGGATGGACTGGTTATTGAGAGCTTAG	
53	ACGTTGGATGGGAAACCTTGGTCTAACGAG	
54	ACGTTGGATGGTAGATTGTAGATTTCGATG	
55	ACGTTGGATGGAAGCCGTACCAAATATGCC	
56	ACGTTGGATGGTTCAACTAATTTCCTTTC	
57	ACGTTGGATGATCGTCGAGATTGAGGAAGC	
58	ACGTTGGATGTCCAATTCGAGCAAGTTTCC	
59	ACGTTGGATGTGCAGAATTGGCACGAATCG	
60	ACGTTGGATGGGGAATTCCTTTCTTGTCCA	
61	ACGTTGGATGATAATGAGGCTGTCTCGTCG	
62	ACGTTGGATGGAAATCCCAACCTGATTGTTC	
63	ACGTTGGATGAGACTACTTTTGATAAGCC	
64	ACGTTGGATGGCTCCTAATGGGACAACATC	
65	ACGTTGGATGCAGAGTCAGTGTATTGAAGAG	
66	ACGTTGGATGCGATTAGCGTGCTCCAAATG	
67	ACGTTGGATGAGTGGAAAGCTCTGTAGTCG	
68	ACGTTGGATGTCCAGTCGTTTACTTAGGAAG	
69	ACGTTGGATGGGCGCTTTCTTTGGTGATAG	
70	ACGTTGGATGGGGAGTTTCGGGTTTAATAC	
71	ACGTTGGATGGCGTGAGAGCCAAATGAATC	
72	ACGTTGGATGCATTTCGAAAAAACAGACAGC	
73	ACGTTGGATGCAGGATTTGCTTGTCACTCC	
74	ACGTTGGATGGGCCTAATTCTTCTTCTGATG	
75	ACGTTGGATGTGTTATAGCTATTGCAGCCG	
76	ACGTTGGATGTGCAATTGCTGTAGCTCGTC	

77	ACGTTGGATGATGGCTAGGCCCTTTTATGG	PCR primers for "Tobacco-OFF-targets" iPLEX/MassARRAY®
78	ACGTTGGATGCTACAAGGTCTCTTGTTTAGG	
79	ACGTTGGATGAGTTCTCCATGAGGTCTACG	
80	ACGTTGGATGAAATGGCTGTTGCTAATGCTC	
81	ACGTTGGATGGATAACATGGAACCTTTCGAC	
82	ACGTTGGATGAGGAATTCTGTGTCCAACTC	
83	ACGTTGGATGATGGGTTTGCCTTGGTATCG	
84	ACGTTGGATGCATCATATTGACAAGCAGGTC	
85	ACGTTGGATGTATCGCTCCCTGTTTGTTGC	
86	ACGTTGGATGTAGCCGTCAACGCTTTAGTC	
87	ACGTTGGATGACGCTAGCAATCAGTACGAG	
88	ACGTTGGATGATTGTTACGATGTGGTTCTGC	
89	ACGTTGGATGGCCCTGCGAGAGTGTATTAC	
90	ACGTTGGATGTTCCTATAAGGGTGGGCATC	
91	ACGTTGGATGACGGACCAATCATTCCCTGC	
92	ACGTTGGATGTCTTTCGCATCTCGATTCCG	
93	ACGTTGGATGGGATACCAGAGATACGGATC	
94	ACGTTGGATGCTTGTTCAAGGGTTGTGGAC	
95	ACGTTGGATGCTTCGATTTCTTCCGGAAAC	
96	ACGTTGGATGATCGGTCTATTTCCGGCATC	
97	ACGTTGGATGCAGTACCAGACACGAGATAG	
98	ACGTTGGATGTCGATTCTTTGGGATCCTTC	
E32	ACCTATATTCCTGATTACGAAT	Extension primers for "Tobacco-OFF-targets" iPLEX/MassARRAY®
E33	ACAGAGGATCTTGAATACTATT	
E34	CACAGAGGATCTTGAATACTATT	
E35	GCAATGGTTTTTTAAACGAGT	
E36	GCTATTTGCTGGAATAATCTAG	
E37	TCTTCTTCTGATGATCGATT	
E38	TCAAAAAGATATACCTTGGTTATATTT	
E39	AGTTGAACATAGTAAAATAAGAAATT	
E40	AAATCAACTCGTATCAACCAAT	
E41	GGTCAATCTAGTACCAATATATTT	
E42	ACCCCTTTCTTCTATATTTTT	
E43	TGACAGACCCATTCTCT	
E44	CCGGACACGAGGTCT	
E45	CCCCGTTTAGTTGACAT	
E46	GGCCGTTGATTGGAATA	
E47	GATGTATTATCTATTCCAACAAATAATA	
E48	TTGCTTTCTGTTCATATAATGCATA	
E49	GGCATGAAGCCACGATCT	
E50	CCCGTTGTCACGTTT	
E51	TAGAATTTGCTAATCGGCT	
E52	GTTTAGTTACAAGAGTTCAATTC	
E53	CAACATACCCCTTTTGC	
E54	CCCGATCTCGCAGATATTT	
E55	CCTATACCAAAAGCTCAGT	
E56	cCCCTGCAAAAATCCTACT	
E57	cATCACATTCCAGCTAACATTT	
E58	TTTTATAACTCTAAAAGTTTTCAAAG	
E59	TGTTGTTCCAATTCGGT	
E60	AAAGGCATTTCGGGAAT	
E61	ATCACTAGCATCAAGATTGT	
E62	CCACTCTTTTTCCTATTCAAAGAT	

E63<br>E64<br>E65	GGATCCTTCCTTTCTTCAAA<br>AATCAGGCCGATTCC<br>CATTGAGGAATATCCAGAAA	Extension primers for "Tobacco-OFF-targets" iPLEX/MassARRAY®
MART16<br>MA4<br>MA5<br>MART26<br>MART28	TGTTGGGAGACAGAACGAAGCTTCGTATACGTCAGGAGTCC<br>GTCAGGGTAACACGACATTGTAGCACCCTCTTGATAGAAC<br>CGCGATAACACAGAACTGACTTATAATCCTCCACTAGTTGG<br>ACCCGTTCGTAAATTGCAATGAAAAAGTAAAAGGTCCCGAGAC<br>CTACAAGGTCTCTTGTTTAGGCATGTGTGTAGGGGGATAATG	cDNA synthesis for tobacco "ndhB-291mini" iPLEX/MassARRAY® assay
6<br>14<br>15<br>16<br>17<br>33<br>34<br>35<br>36<br>37	ACGTTGGATGCGCGATAACACAGAACTGAC<br>ACGTTGGATGCAAATGTATAACTCCCCAGG<br>ACGTTGGATGGTCAGGGTAACACGACATTG<br>ACGTTGGATGACCCGTTCGTAAATTGCAATG<br>ACGTTGGATGCTACAAGGTCTCTTGTTTAGG<br>ACGTTGGATGTGTTGGGAGACAGAACGAAG<br>ACGTTGGATGCTAGACAGTCGTTGCTTTTC<br>ACGTTGGATGGGTCCAAGTGTATCTTGTCT<br>ACGTTGGATGTAGCTACTTTAGCGGCTTGG<br>ACGTTGGATGTAATCAGTCATATCCTGTGG	PCR primers for tobacco "ndhB-291mini" iPLEX/MassARRAY® assay
E19<br>E20<br>E21<br>E22<br>E23<br>E24<br>E25	GTCAGGAGTCCATTGATGA<br>GGAGTCGAAAGTAGCTGCTT<br>TCGAAAATTCGAGTGGCT<br>CCAGGATCGGATCCTTTGAT<br>CAAGTGTATCTTGTCTTTACTA<br>TTTACAGTGGTCAAATTGGAT<br>TGCTTTTGCTGTCAAAT	Extension primers for tobacco "ndhB-291mini" iPLEX/MassARRAY® assay
MA9<br>MA10<br>MA11<br>MA12<br>MA13<br>MA14<br>MA15<br>MA16	TACCGCAGGATCAACTAGTCGAAATTTAATGGGATCCGCAG<br>AGCTTTACACCAGTGTTGTTGTTTTTAGCTTGTTGATAGAGG<br>AGTATCACCAACATGACAGTCTCCATAGAATACAATTCGCTC<br>GTACCGGAGACATTAACACGATCCGATTTGACCTATGGACG<br>CAGGATTTGCTTGTCACTCCAATAAGGTCCGCCTGTCTAGG<br>CTAAATATAGCCTGGTAGACCTTCAGGGTAGCAAACATTCTC<br>AGGACTTGCTTTGAAGCACCTCTTGCAAATAAGGCATATCC<br>GAAATCCCAACCTGATTGTTCAATGTAAACCATAAGTTTCAG	cDNA synthesis for "Arabidopsis-QED1-targets" iPLEX/MassARRAY® assay
1<br>2<br>3<br>4<br>5<br>6<br>7<br>8<br>21<br>22<br>23<br>24<br>25<br>26<br>27<br>28	ACGTTGGATGCAATGCGACAATTGTTATGG<br>ACGTTGGATGGAGCGAGTTATTTCAGCTCC<br>ACGTTGGATGTTCGGTTCTGTTTATACGAG<br>ACGTTGGATGGTACCGGAGACATTAACACG<br>ACGTTGGATGCCCATACTTGATCGAACAAC<br>ACGTTGGATGCGCGATAACACAGAACTGAC<br>ACGTTGGATGTGGGTTCAGAAAAAGGGTGG<br>ACGTTGGATGGCGACTCGTTTAGATCATAG<br>ACGTTGGATGTACCGCAGGATCAACTAGTC<br>ACGTTGGATGAGCTTTACACCAGTGTTGTTG<br>ACGTTGGATGAGTATCACCAACATGACAGTC<br>ACGTTGGATGCCAGTCGTTGCTTTTCTTTC<br>ACGTTGGATGCAGGATTTGCTTGTCACTCC<br>ACGTTGGATGCCTTTAATGAATTCCCTTG<br>ACGTTGGATGAGGACTTGCTTTGAAGCACC<br>ACGTTGGATGGAAATCCCAACCTGATTGTTC	PCR primers for "Arabidopsis-QED1-targets" iPLEX/MassARRAY® assay
E1<br>E2<br>E3<br>E4	TCAAGTACCCGGATCAATC<br>GCTTGTTGATAGAGGTTTACT<br>TGGATTCGTATTCACATACAT<br>TCGAAAGTAGCTGCTT	Extension primers for "Arabidopsis-QED1-targets" iPLEX/MassARRAY® assay

E5	AGCGAAAATTCGAGTGGCT	Extension primers for "Arabidopsis-QED1-targets" iPLEX/MassARRAY® assay
E6	TTTTCGATATTCCTTTTTATTTCT	
E7	CCATAGGCTGGAAAGTCTT	
E8	TGAATTCCCTTGGAACTT	
E9	AACAAAAAATATCTATTCTAGTTCTAT	
E10	ACTTTTATTTCACGTTTCTGT	
E11	TTCGTCATAGGTACTGATCCGT	
P12<br>nb11	TTCATGCTTGTTTGAGTAATAGC<br>GGTCTAATGAGGCTACTATG	Sequencing of *ndhB* (div. species)
oDL172<br>Paada25a	TAGCACCCTCTTGATAGAAC<br>AGATCACCAAGGTAGTCGGCAA	Sequencing of *ndhB*-291mini (pRB58)
accD_for<br>accD_rev	CAGTACCCGAAGGTTCACAAGC<br>AACTCACTCTTGTTGATAAAGGC	Sequencing of tobacco *accD* 3´ UTR
oVL180<br>oVL181	CTACGCGCAAATTCTCATTGG<br>CAGGTTGATGCAATGTATTCCACC	Sequencing of tobacco *ccmC*
oVL59<br>oVL60	AGAATTATGTAATTAAGGGACTCTTC<br>GCCCGCTCTTGTATTTGTAATAAC	Sequencing of tobacco *ccsA*
oDL123<br>oDL125	GGTCCAAGTGTATCTTGTCTTTAC<br>CCCACATGATGAAAAAAAGTAAAAG	Sequencing of tobacco *ndhD*
oVL140<br>oVL141	CTATGGATTGATCACAAGTCG<br>ATTTATCAATATTAGAATCTAGATG	Sequencing of tobacco *ndhE*
oVL36<br>oVL52	TTTGTTTCTGTATTTCCGGAGTATG<br>CGATGATTCAATAAAAAAACCTAACC	Sequencing of tobacco *petB* intron
oVL142<br>oVL143	GTGTGGGGAAGAAGTGGACTC<br>GCATAGTATCTCGTTAGACAACC	Sequencing of tobacco *petN* 3´ UTR
oVL138<br>oVL139	TATCTATATTATATTAAGTAGATTG<br>CAGAGTATATCTTTCCACGG	Sequencing of tobacco *psbD* 5´ UTR
A1a<br>A2a	GCCCTAATGACTATAGATCGAACC<br>ACCCCCAGTAGAGACTGGTACG	Sequencing of tobacco *psbEFLJ*
rps12_for<br>rps12_rev	CAACTTATTAGAAATACAAGACAGC<br>ATCCCATTCACCGGTACTGATC	Sequencing of tobacco *rps12* intron
At_E5<br>At_E6	TTATTACGTAAGGGCTTATTGG<br>GTTTGTCTAGTCTAATTTGAACTTC	Sequencing of *Arabidopsis accD*
At_E7<br>At_E8	CGTTACCGGGTAAAAGATGC<br>AGCGGCGTATCCTTTGTTGC	Sequencing of *Arabidopsis matK*
At_E1<br>At_E2	GAGGTGGGTTCAGAAAAAGG<br>TATCTGTCCTACATTCATGCG	Sequencing of *Arabidopsis rpoB*
At_E3<br>At_E4	TACAAGACAGCCAATCCGAAAC<br>GTTGATTGGATTTGCACCAATG	Sequencing of *Arabidopsis rps12*
Tc_E7<br>Tc_E8	CTGAGTGAGTTAGTTCAGCTCCACG<br>TTTATTTGTCTCGCCAAATTCG	Sequencing of cacao *accD*
Tc_E1<br>Tc_E2	TAGAAATCTTGGTTCAAGCTCTTCG<br>CCACCTATTCATCAGAAGAGACGTATC	Sequencing of cacao *matK*
Tc_E3<br>Tc_E4	ACCTATAGGTGGGAGGGGTCG<br>AGCGAGCATTCAAATATCTGTCC	Sequencing of cacao *rpoB*
Tc_E5<br>Tc_E6	AATGTCACGAAATCCCCTGCTC<br>CGCTTAATGGATAAGCATTTGC	Sequencing of cacao *rps12*
Gh_E7<br>Gh_E8	GAGTGAGTTAGTTCAGCTCCACGG<br>ATTCGAATTTCATTTCACAGAAGG	Sequencing of cotton *accD*
Gh_E1<br>Gh_E2	CTTCGCTACTGGGTAAAAGATGC<br>CAGAAGAGACGTATCCTTTGAGGC	Sequencing of cotton *matK*
Gh_E3<br>Gh_E4	GAGTCATCGATGTGAGATGGGTCC<br>TCTGTCCTACATTCATTCGTGAAGG	Sequencing of cotton *rpoB*

Gh_E5	CCTGCTCTTGGGGGATGTCCT	Sequencing of cotton *rps12*
Gh_E6	GCATTTGCTACCAATGGGGAAT	
Cp_E7	TCACGGTTTTTTTCCTTTGAATC	Sequencing of papaya *accD*
Cp_E8	ATTTTTATTAATAAGCATGGAATTG	
Cp_E1	CCCTTCGGTACTGGATAAAAGATAC	Sequencing of papaya *matK*
Cp_E2	TCCATTTATTCATCAGAAGAGGCG	
Cp_E3	GGTCGGGTTATTGATGTAAGATGG	Sequencing of papaya *rpoB*
Cp_E4	TCAAATAGCTGTCCTACATTCATTCG	
Cp_E5	CGCTCTTCGGGGATGTCCTC	Sequencing of papaya *rps12*
Cp_E6	GGTGATTGGATTTGCACCAATGG	
Sl_E5	CACGCCTTTTTTCCTTTGAATC	Sequencing of tomato *accD*
Sl_E6	ATTTTACTAATTTCACGAAAGGAAG	
Sl_E1	GAAATCTTGGTTCAAACTCTTCGC	Sequencing of tomato *matK*
Sl_E2	TTATTCATCCAAGGAAACGTCCC	
Sl_E3	TGCCCTCAGCGTCGAGGAAC	Sequencing of tomato *rps12*
Sl_E4	TGGATTTACACCAACGGAAACC	
oDL206	GATATTGGGAAATCTCATTGC	Generation of the *ndhB* probe
oDL207	GTAATACGACTCACTATAGGGTAGAAACAACGCTTGTAAGG	
oK77	GGTCCTAATGATGATCCTGC	Generation of the *petB* exon probe
oK78	TAATACGACTCACTATAGGGCCCAGAAATACCTTGTTTAC	
K79	GACTTGAATGATCCTGTATTAAGAGC	Generation of the *petD* exon probe
oVL125	TAATACGACTCACTATAGATGTTGCACCAATGCCCAACC	
oVL57	AGGGATCTAAAGAAGAAGAGAGGC	Generation of the *petD* intron probe
oVL124	TAATACGACTCACTATAGATCCCCCGAGGGAACTGGAC	
oK75	CGTCAGTTATAGTGATCCTGCTAC	Generation of the *psbB* probe
oK76	TAATACGACTCACTATAGGGAATGCTCCAAATTCGAC	
oK71	ATGGCTACACAAACTGTTGAAAAC	Generation of the *psbH* probe
oK72	TAATACGACTCACTATAGGGCTAATTCATTGAAATTCCATCC	
P_UBQ10for	ttctcgagCCGACGAGTCAGTAATAAAC	Cloning of *A. thaliana UBIQUITIN 10* promoter
P_UBQ10rev	ttgaattCAGTGTTAATCAGAAAAACTC	
B11	agttcgGGTACCATGGCTATCTTCTCCACAGCACA	Cloning of *A. thaliana* QED1
B12	tcagtaACTAGTTCACCAGAAATCGTTACAGGAACA	
oVL66	cgatactgcaggagctcggtacATGGAAACTCTAGGCACCCGTCTC	Cloning of *T. cacao* QED1
oVL67	agggatatcactagtaaaaggtacTCACCAATAATCCTTACATGAACAATG	
ot-05	aatgcctatagttcctagtgattctGTCTGGGGAGCGCTTCTAGGAGC	Cloning of *Arabidopsis*-cacao chimeras
ot-07	ttgtttgatatgatggaggagaagGATAACGTTACATGGACAACAATGCTTG	
ot-08	aagtgcatgtctagaGGAGGAGGAACCAATAACGAACGCTCCCGC	
ot-09	catagttgtccacgtgacaatatcTTTCTCCTCCATTGCATCAAACAGTC	
ot-10	gctccaagaagagccccccatacAGAAGTGCTTGGAGGAATCGGCA	
otHA6	aaactgggtcgtcatggtcgttatt*TCCTCCTCC*TCTAGACATGCACTTTACTCTTCC	
otHA7	gtggcgggagcgttcgttattggt*TCCTCCTCC*TCTAGACATGCACTTTACTCTTCC	
oVL126	atgagtgaggttgtaagatgacaATTCATCCTAATACCGTGCTTTTTG	
oVL127	aaaagcacggtattaggatgaatTGTCATCTTACAACCTCACTCA	
oVL128	atgagagcattccatgcagcaatATCTTTCTGAGGCATAGAATTAAG	
oVL129	ttaattctatgcctcagaaagatATTGCTGCATGGAATGCTCTC	
oVL130	aaagtacttaccagtgtgatctgATCAGGTTTAGCAATCTTGCTCAG	
oVL131	tgagcaagattgctaaacctgatCAGATCACACTGGTAAGTACTTTATCAGC	
oVL132	agagtgctaacaagagtgatctcGTTCAGCTTCATGTTCTTCTG	
oVL133	tcagaagaacatgaagctgaacGAGATCACTCTTGTTAGCACTCTATC	
oVL134	aagagcattccaagcaacaatATCTTGCCTTGGCATGATATCAAGAAC	
oVL135	tgatatcatgccaaggcaagatATTGTTGCTTGGAATGCTCTTAT	
oVL67	agggatatcactagtaaaaggtacTCACCAATAATCCTTACATGAACAATG	
oVL80	cgatactgcaggagctcggtacATGGCTTCTATGATATCCTCTTC	

oVL81	agggatatcactagtaaaaggtacTCACCAGAAATCGTTACAGGAACACTG	Cloning of *Arabidopsis*-cacao chimeras
Tc-09	gtaaagtgcatgtctaga*GGAGGAGGA*AATAACGACCATGACGACCCAGTTT	
Tc-10	aagctcctagaagcgctccccagacAGAATCACTAGGAACTATAGGCATTTTC	
Tc-11	cattgttgtccatgtaacgttatcCTTCTCCTCCATCATATCAAACAATCTT	
Tc-12	ctgtttgatgcaatggaggagaaaGATATTGTCACGTGGACAACTATGCTTG	
Tc-13	gccgattcctccaagcacttctGTATGGGGGGCTCTTCTTGGAG	
qTac9actin_f	CCTGAGGTCCTTTTCCAACCA	qRT-PCR, Housekeeping tobacco gene (Schmidt and Delaney 2010)
qTac9actin_r	GGATTCCGGCAGCTTCCATT	
oVL147	TGTTCTCGGCCGCTCGGG	qRT-PCR, *A. thaliana* QED1
oVL148	AGGAATCGGCATCGCCTCTATG	
oVL155	GCGGGAGATAACCGCCATCC	qRT-PCR, *T. cacao* QED1
oVL156	AACCAACTGATTTTAATCTCGCCAC	

Supplementary Table S14. Coverage of identified SNPs in the RNA-seq libraries.
List of putative sites targeted by Arabidopsis QED1 in tobacco chloroplasts (cp) and mitochondria (mt) identified by RNA-seq. SNP positions are given based on the tobacco chloroplast (NC_001879) or mitochondrial (NC_006581) genomes. Maximum coverage is given as number of mapped reads spanning the position where the SNP was detected. Maximum detected editing efficiency (%) is given as T/C or A/G ratios at the SNP position. Location of the identified SNPs is indicated. CDS: coding region; UTR: untranslated region; as: antisense; intr: intron. If editing occurs in a coding region, the change in the encoded amino acid is indicated (Impact).

	SNP coordinate	Max coverage	Max editing %	Site	Location	Impact
cp	5800	3,491	9.5	***rps16*_intr**	intron	-
	7359	984	32.9	***trnQ*_as**	as	-
	12570	9,511	20.3	***atpF*-65**	CDS	R->STOP
	16335	1,273	72.2	***rps2*-203**	CDS	S->L
	29881	5,935	15.0	***petN*_3UTR**	3´ UTR	-
	31530	1,310	36.3	***trnD-psbM*_intergenic**	intergenic	-
	32653	26	7.7	***trnE*_intergenic-upstream**	intergenic	-
	33880	3,250	37.9	***psbD*_5UTR**	5´ UTR	-
	61515	1,179	5.0	***accD*_3UTR**	3´ UTR	-
	64242	1,465	13.7	***ycf10-petA*_intergenic**	intergenic	-
	69550	4,117	11.5	***psaJ*_5UTR**	5´ UTR	-
	74956	11,182	11.8	***psbB*-27**	CDS	T->I
	77495	4,953	34.0	***petB*_intr**	intron	-
	77612	4,541	87.4	***petB*_intr-mid**	intron	-
	78993	10,740	17.6	***petB-petD*_intergenic**	intergenic	-
	83340	1,973	9.7	***rpl14*-69**	CDS	synonymous
	90203	448	5.4	***ycf2*-440**	CDS	T->M
	90230	473	5.1	***ycf2*_as**	as	-
	90236	459	5.4	***ycf2*-451**	CDS	P->L
	90244	447	5.1	***ycf2*-454**	CDS	L->F
	93937	397	6.8	***ycf2*_as2**	as	-
	95383	812	6.7	***ycf2*-2167**	CDS	Q->STOP
	98961	4,175	26.6	***ndhB*-101**	CDS	Q->STOP
	114743	1,612	14.7	***rpl32*_5UTR**	5´ UTR	-
	115955	40	5.0	***rpl32-trnL*_intergenic-as**	intergenic, as	-
	116881	1,588	26.8	***ccsA-182***	CDS	S->L
	119671	3,529	44.2	***ndhE*-96**	CDS	S->L
	120048	3,372	5.6	***ndhG-ndhE*_intergenic**	intergenic	-
	124713	2,678	14.9	***ndhH*-136**	CDS	R->STOP
	128607	97	5.2	***ycf1_as***	as	-
	129614	1,043	14.9	***ycf1*-661**	CDS	synonymous
mt	19520	3,638	46.7	***ccmC*-83**	CDS	H->Y

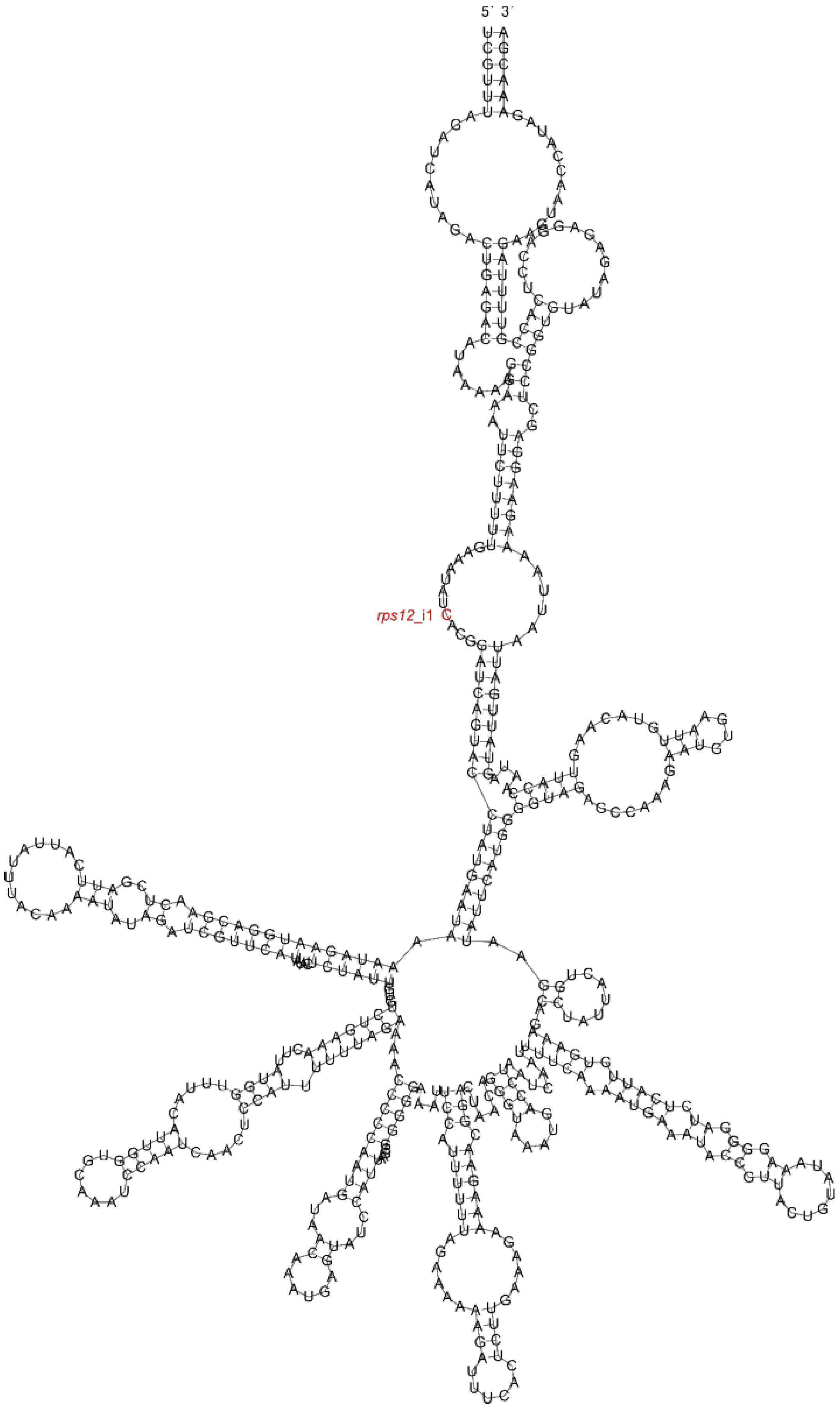

Supplementary Figure S1. *In silico* folding of Domain I of the *trans*-spliced *rps12* intron 1.
The RNA secondary structure of Domain I of the intronic sequence of *rps12* was predicted using the RNAfold webserver (http://rna.tbi.univie.ac.at//cgi-bin/RNAWebSuite/RNAfold.cgi; (Hofacker 2009)). The optimal secondary structure shown was predicted with a free energy of folding of -94.7kcal/mol. The editing site is highlighted in red and resides within a predicted loop/non-basepaired region.

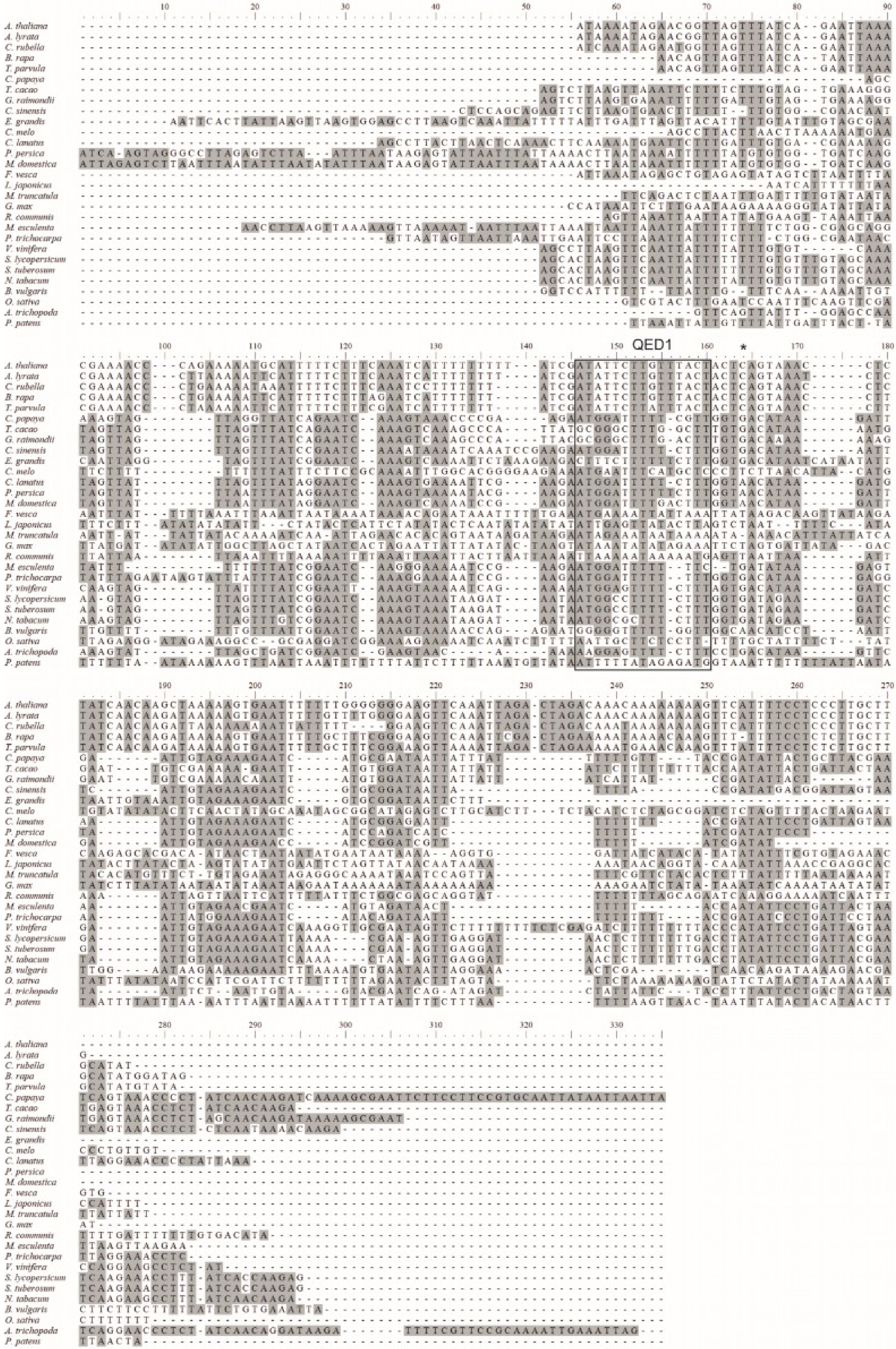
QED1
A. thaliana
A. lyrata
C. rubella
B. rapa
T. parvula
C. papaya
T. cacao
G. raimondii
C. sinensis
E. grandis
C. melo
C. lanatus
P. persica
M. domestica
F. vesca
L. japonicus
M. truncatula
G. max
R. communis
M. esculenta
P. trichocarpa
V. vinifera
S. lycopersicum
S. tuberosum
N. tabacum
B. vulgaris
O. sativa
A. trichopoda
P. patens

Supplementary Figure S2. Sequence alignment of the *accD* 3´ UTR from various species.

Alignment of the nucleotide sequence 200 bp downstream of the stop codon of *accD* from the species in Figure 1B. Conserved residues are shaded in grey (identity threshold: 50%). The position corresponding to *Arabidopsis* editing site *accD*_3UTR (nucleotide position 58,642 in NC_000932.1) is marked by an asterisk. The box marks the predicted binding site of *Arabidopsis* QED1 (based on the sequence of the *Arabidopsis accD*_3UTR site).

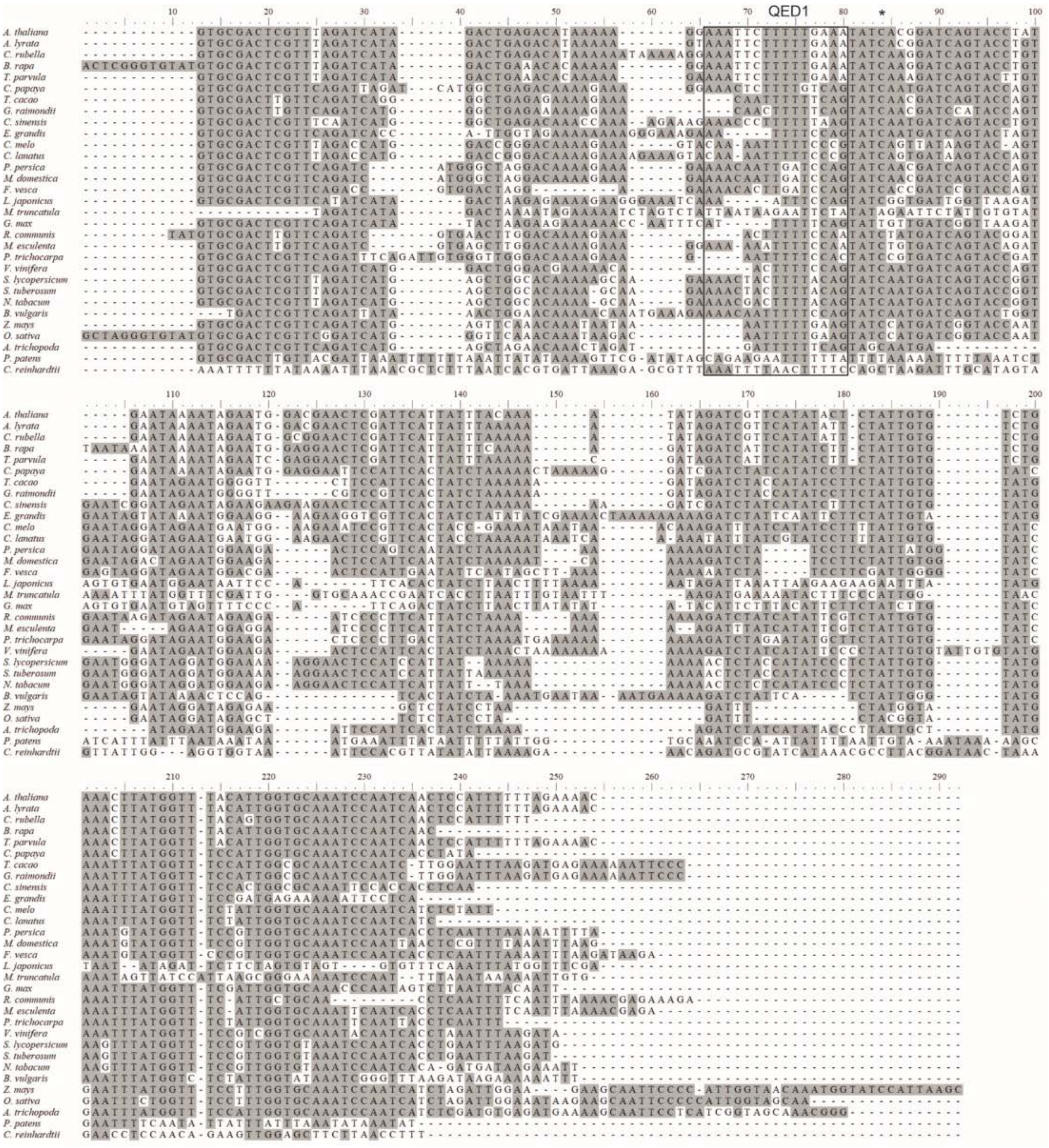

Supplementary Figure S3. Partial sequence alignment of *rps12* intron 1.

Alignment of the nucleotide sequence 200 bp downstream of the first exon of *rps12* from the species in Figure 1B. Conserved residues are shaded in grey (identity threshold: 50%). The position corresponding to *Arabidopsis* editing site *rps12*_i1 (nucleotide position 69,553 in NC_000932.1) is marked by an asterisk. The box marks the predicted binding site of *Arabidopsis* QED1 (based on the sequence of the *Arabidopsis rps12*_i1 site).

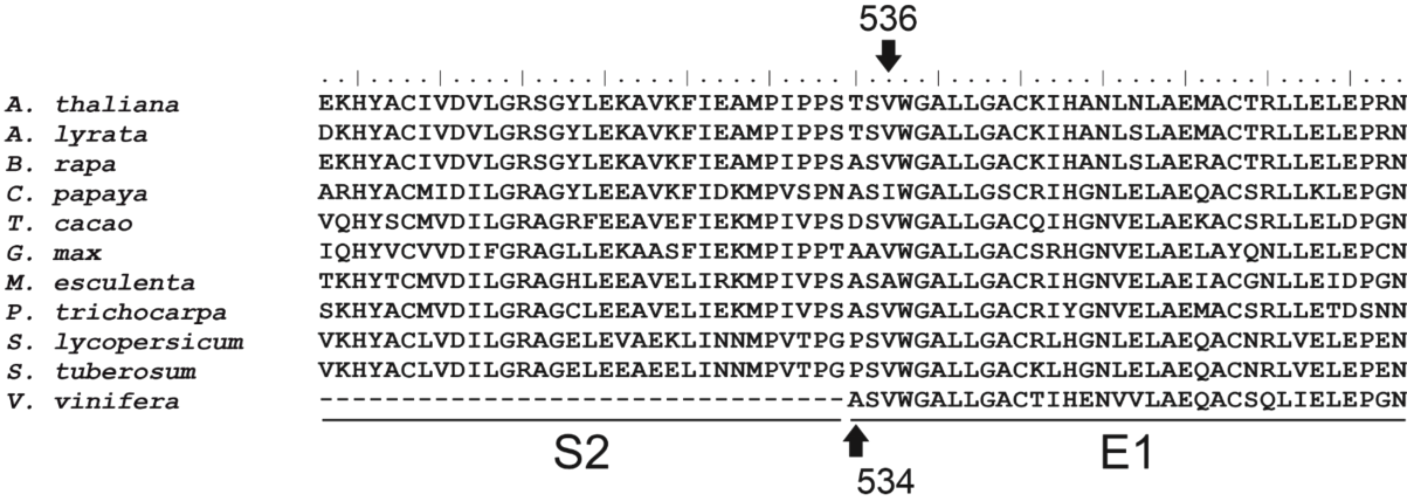

Supplementary Figure S4. Non-functional chimeric QED1 proteins.

Partial alignment of QED1 amino acid sequences from selected species. The last PPR motif (S2, motif no. 15) and the E1 domain annotated based on (Cheng, et al. 2016) are indicated by black horizontal lines. Black arrows indicate the first amino acid of the E1 domain based on the most recent annotation (Cheng, et al. 2016) (*Arabidopsis* residue 534) and of the first amino acid of the previously annotated E domain (Lurin, et al. 2004) (*Arabidopsis* residue 536), respectively. Note that amino acids at positions 535-536 of *Arabidopsis* and amino acids at positions 530-531 of *T. cacao* were included as part of the PPR tract in our constructs (Table 1). Cacao carries a negatively charged aspartate residue (D) while *Arabidopsis* carries a threonine (T) at position 534 (first amino acid of the E1 domain). An aspartate at the beginning of E1 is not found in any other QED1. Hence, it seems possible that this residue is incompatible with the C-terminus of *Arabidopsis* QED1, resulting in non-functional At_Cterm and At_PPR1-8+Cterm proteins (Table 1). Full species names are reported in Figure 1.

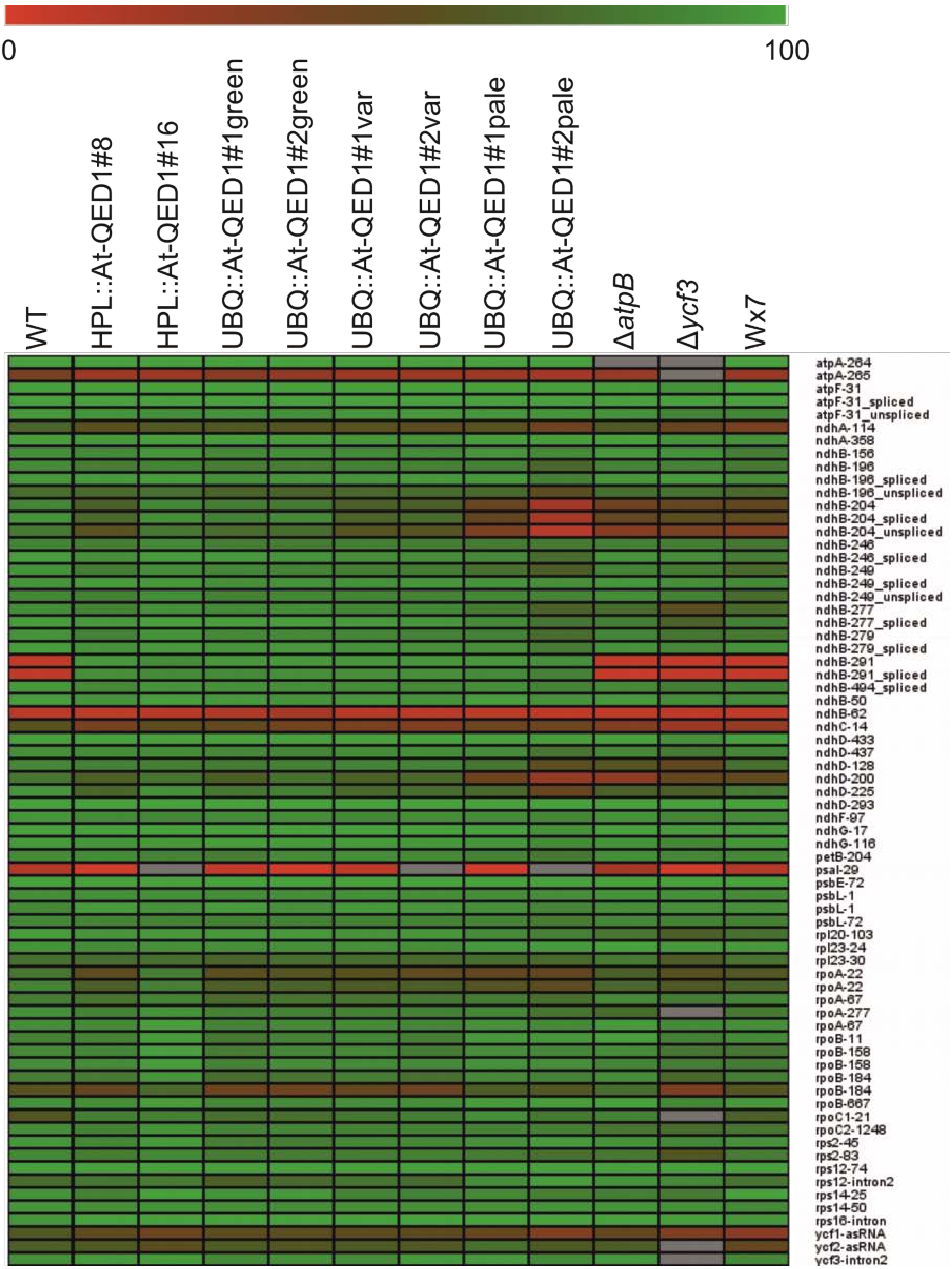
0
100
WT
HPL::At-QED1#8
HPL::At-QED1#16
UBQ::At-QED1#1green
UBQ::At-QED1#2green
UBQ::At-QED1#1var
UBQ::At-QED1#2var
UBQ::At-QED1#1pale
UBQ::At-QED1#2pale
ΔatpB
Δycf3
Wx7
atpA-264
atpA-265
atpF-31
atpF-31_spliced
atpF-31_unspliced
ndhA-114
ndhA-358
ndhB-156
ndhB-196
ndhB-196_spliced
ndhB-196_unspliced
ndhB-204
ndhB-204_spliced
ndhB-204_unspliced
ndhB-246
ndhB-246_spliced
ndhB-249
ndhB-249_spliced
ndhB-249_unspliced
ndhB-277
ndhB-277_spliced
ndhB-279
ndhB-279_spliced
ndhB-291
ndhB-291_spliced
ndhB-494_spliced
ndhB-50
ndhB-62
ndhC-14
ndhD-433
ndhD-437
ndhD-128
ndhD-200
ndhD-225
ndhD-293
ndhF-97
ndhG-17
ndhG-116
petB-204
psaI-29
psbE-72
psbL-1
psbL-1
psbL-72
rpl20-103
rpl23-24
rpl23-30
rpoA-22
rpoA-22
rpoA-67
rpoA-277
rpoA-67
rpoB-11
rpoB-158
rpoB-158
rpoB-184
rpoB-184
rpoB-667
rpoC1-21
rpoC2-1248
rps2-45
rps2-83
rps12-74
rps12-intron2
rps14-25
rps14-50
rps16-intron
ycf1-asRNA
ycf2-asRNA
ycf3-intron2

Supplementary Figure S5. Chloroplast editotype of *Arabidopsis* QED1-expressing transgenic tobacco plants.
The complete chloroplast editotypes of the QED1-expressing transgenic lines and wild-type tobacco plants were assessed using the iPLEX/MassARRAY® technology (see Material and Methods). Pale, variegated (var) and green tissue from the UBQ::At-QED1 overexpression lines was separately harvested and analysed. Data are presented with Multiple Array Viewer (MeV). The color scale indicates the editing percentage (T/C signal ratio) from 0% (red) to 100% (green). Grey: failed assay. Values for the QED1 mutants represent averages of two technical replicates. Values for the wild type (WT) represent averages of five biological replicates. Three previously generated pale mutants (Δ*atpB*, Δ*ycf3* and WX7; see Material and Methods for a detailed description of the lines) were included as controls for the pale phenotype.

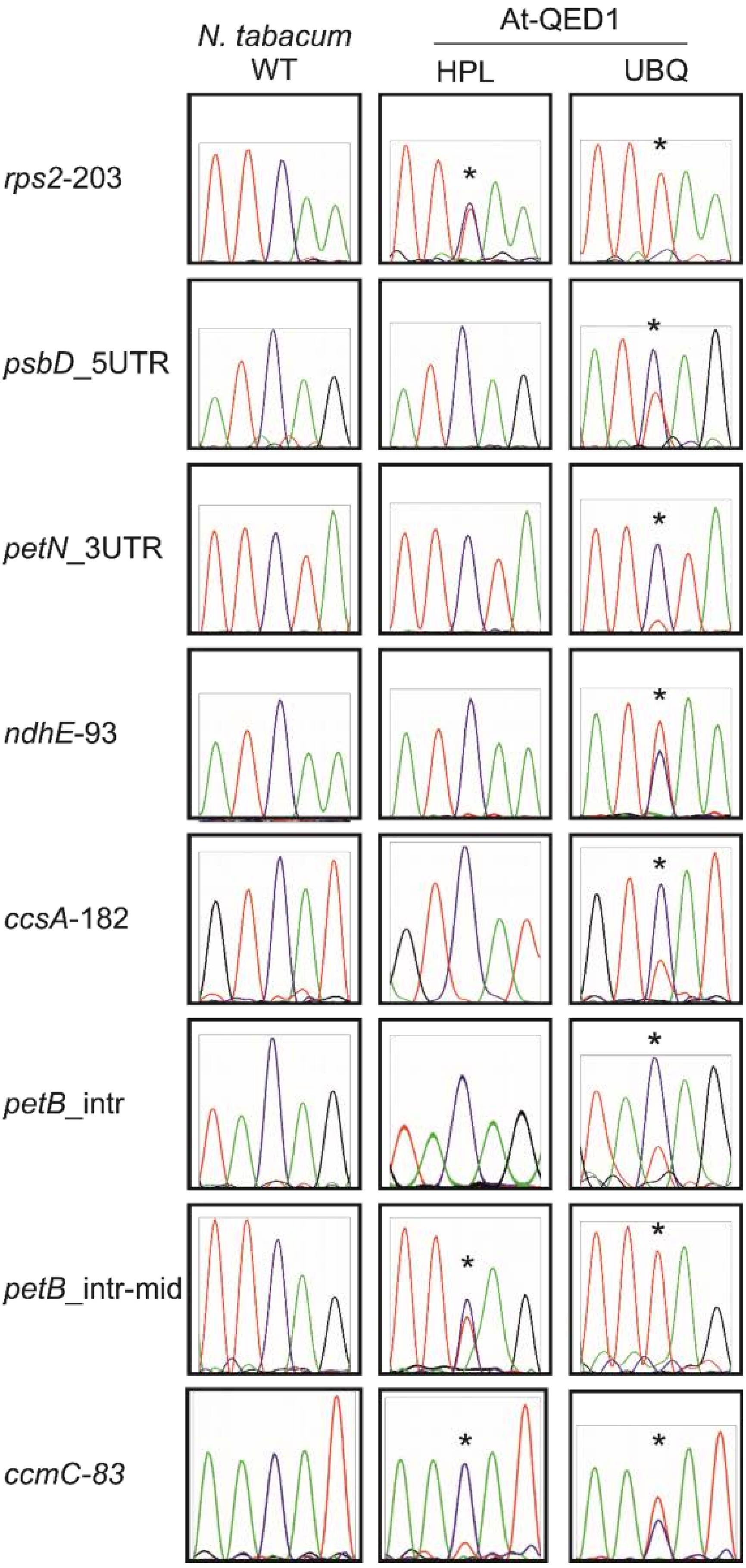

Supplementary Figure S6. Validation of selected QED1 off-targets by Sanger sequencing.
Seven plastid and one mitochondrial off-target sites of *Arabidopsis* QED1 in tobacco identified by RNA-seq in tobacco (Table 2) were validated by bulk sequencing of amplified cDNA in one HPL and one UBQ line. Asterisks indicate C-to-U conversions by off-target editing.

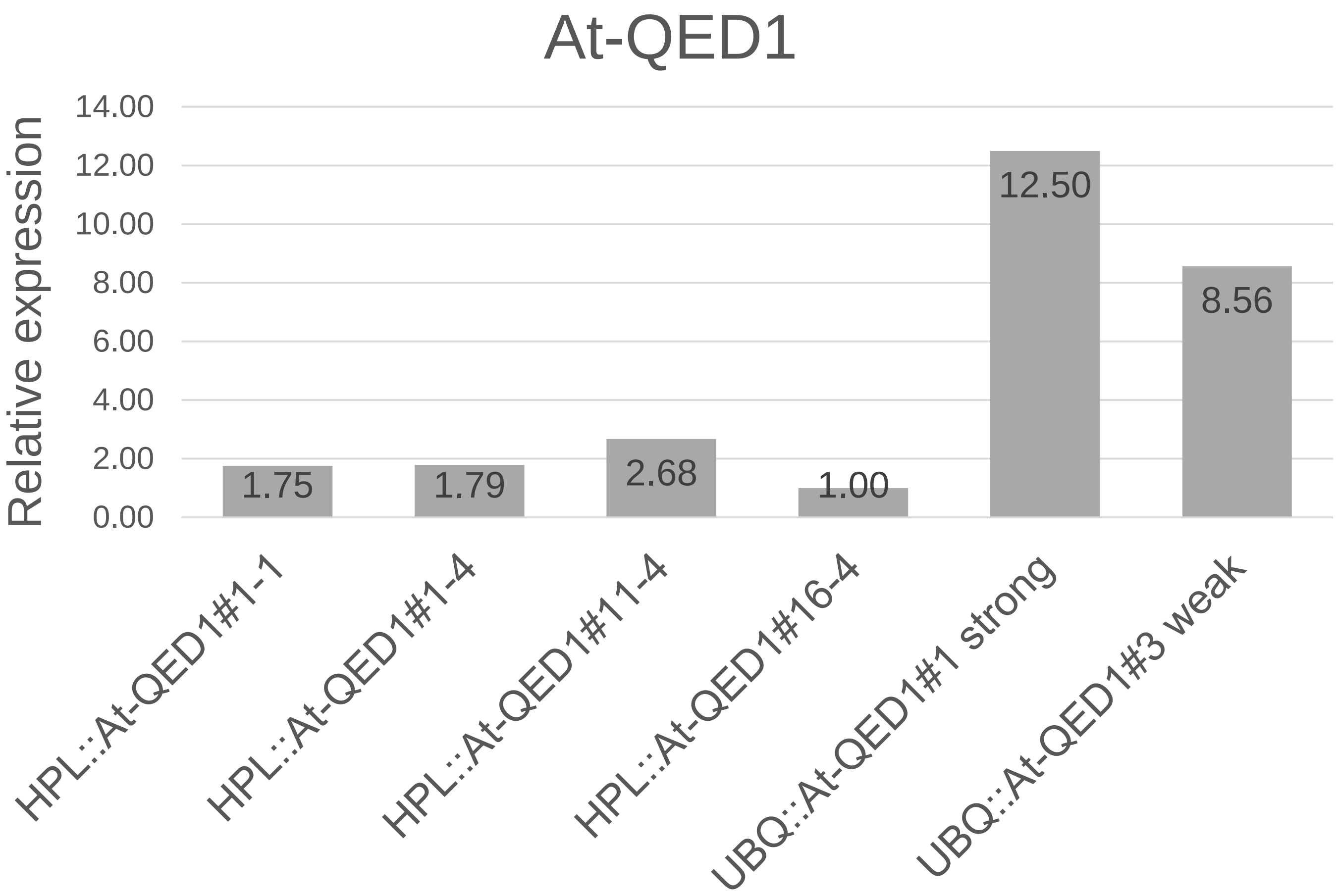

Supplementary Figure S7. *QED1* expression analysis by qRT-PCR.
Relative expression of transgenic tobacco lines expressing *Arabidopsis*-QED1 (At-QED1) quantified by real-time PCR (qRT-PCR). Because the target gene is only present in the transgenic lines and not in wild-type plants, relative expression values are reported as ΔΔCt fold changes to the transgenic line with the highest ΔCt value, hence, the lowest expression level (HPL::At-QED1#16-4 for *Arabidopsis* QED1). Raw data and calculations are provided in Supplementary Table S7.

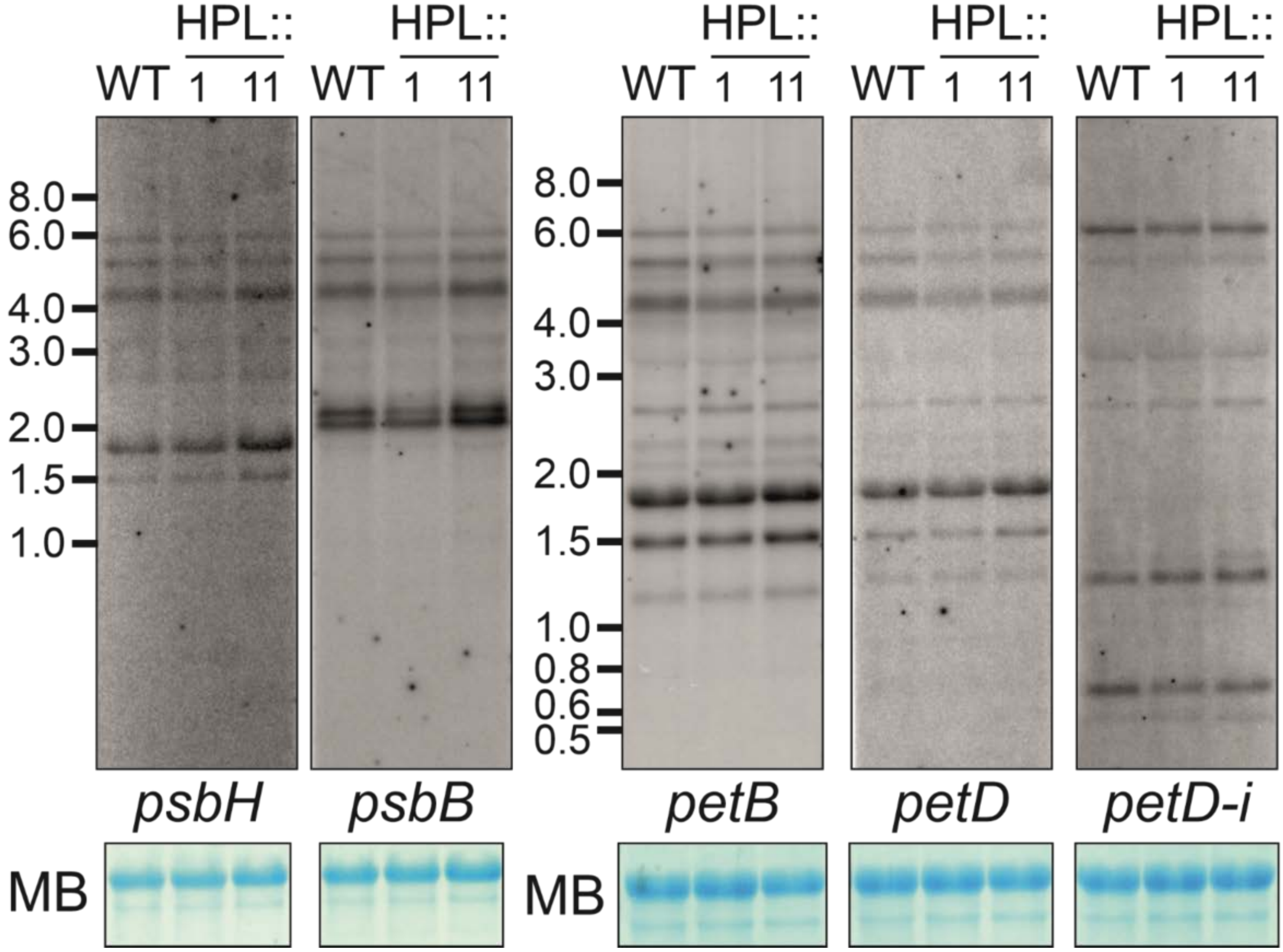

Supplementary Figure S8. Northern blot analyses of *psbB* operon transcripts.
Single-stranded RNA probes were designed against the coding regions of *psbH*, *psbB*, *petB* and *petD*, and the intron sequence of *petD* (*petD-i*). An additional probe was generated for the intron sequence of *petB*, but yielded only low-intensity signals of poor quality.Young emerging green leaves were harvested from six week-old wild-type tobacco (WT) and HPL lines (HPL::At-QED1#1-1 and HPL::At-QED1#11-04-3 shown in Figure 5D and E). Samples of 5 µg total RNA were loaded. Sizes of marker bands are given in kb. Methylene blue (MB) staining of an rRNA-containing part of each blot is shown as loading control.

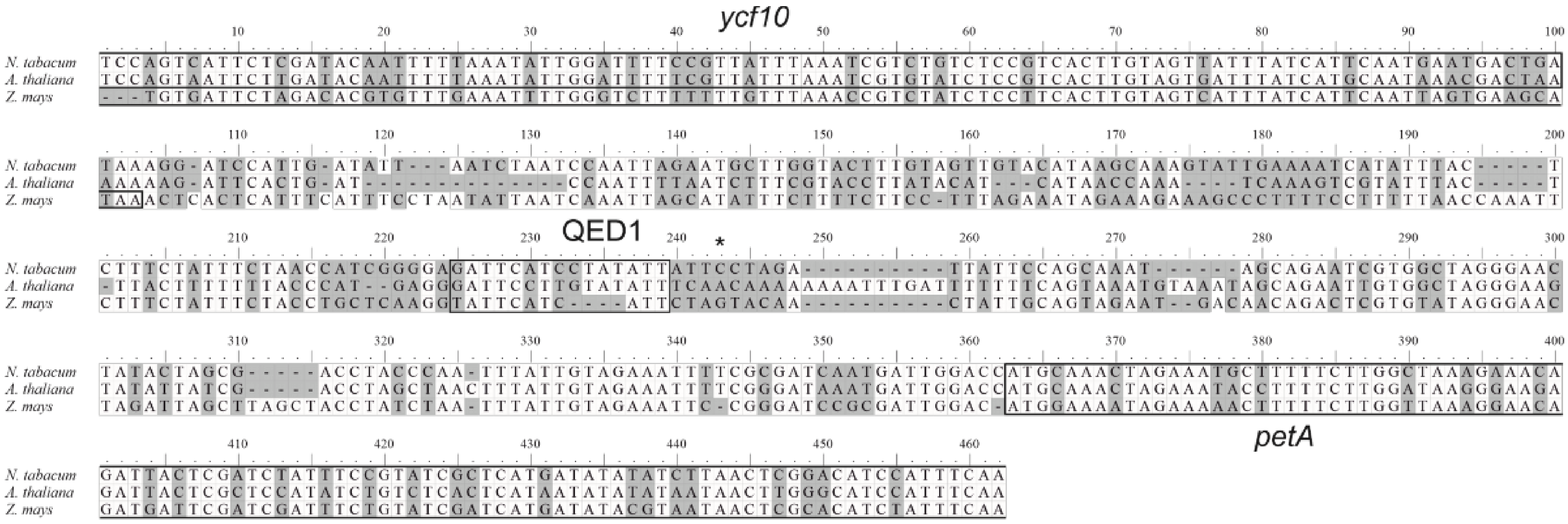

Supplementary Figure S9. An off-target binding site of *Arabidopsis* QED1 in the *ycf10-petA* intergenic region.
Nucleotide sequence alignment of the *ycf10-petA* region from *N. tabacum*, *A. thaliana* and *Z. mays*. The position corresponding to the off-target editing site *ycf10-petA* edited by *Arabidopsis* QED1 in tobacco (Table 2) is indicated by the asterisk. The predicted QED1 binding site is boxed. Note that no other potential QED1 binding sites were found, despite using a degenerated consensus in the search. In addition to the spacer sequence, the last 100 nt of *ycf10* and the first 100 nt of *petA* are included in the alighment. Residues differing between the three species are shaded in grey.

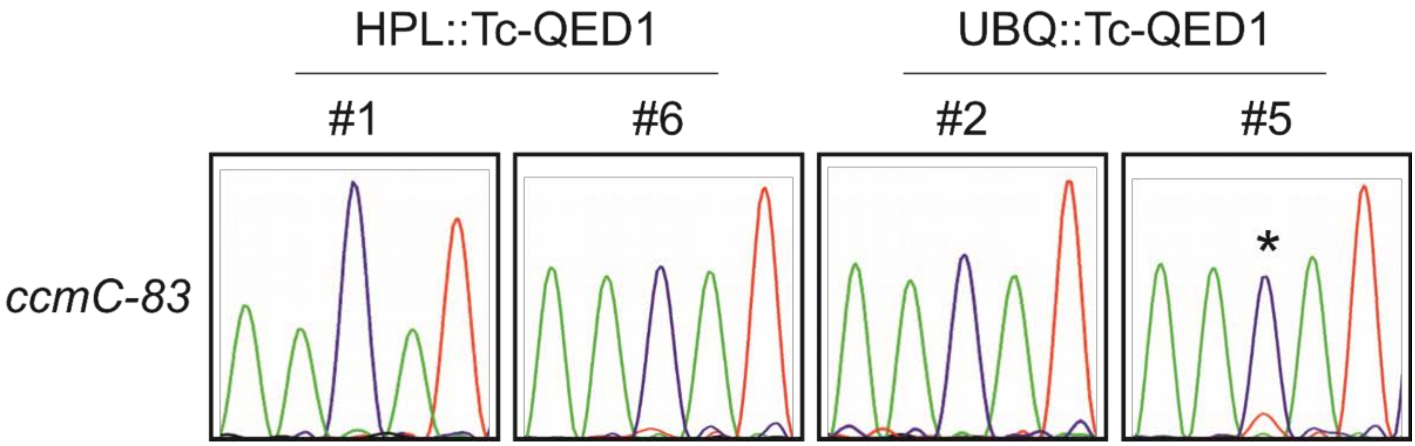

Supplementary Figure S10. Editing at *ccmC*-83 by the cacao QED1 protein.
Editing at the mitochondrial *ccmC*-83 off-target site by the cacao QED1 (Tc-QED1) was evaluated by bulk sequencing in two independent HPL and two independent UBQ lines. Note that lines UBQ::Tc-QED1#2 and #5 were analyzed by iPLEX/MassARRAY® for editing at chloroplast off-target sites (Table 5). The asterisk indicates the partial C-to-U conversion at site *ccmC*-83.

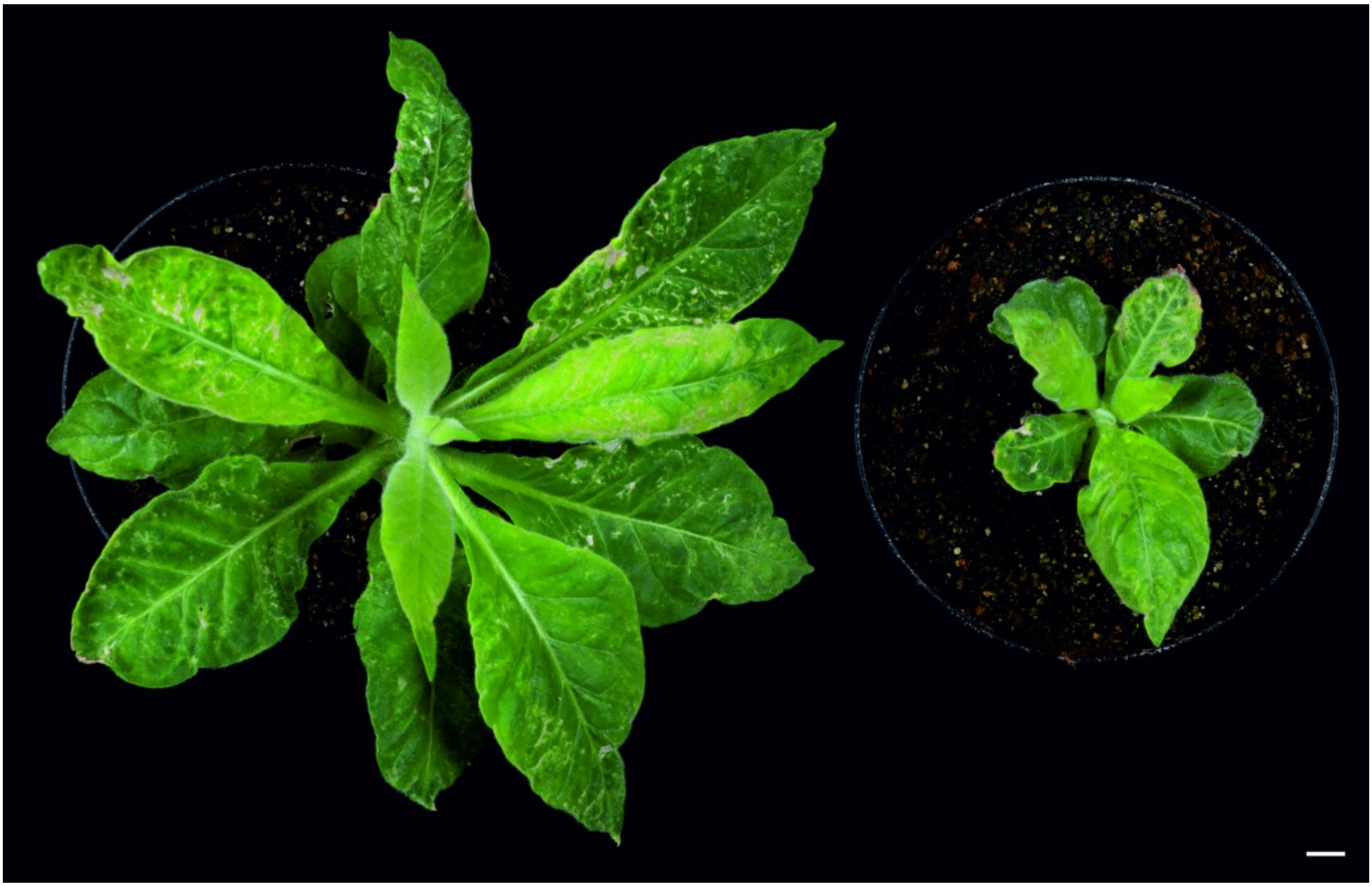

Supplementary Figure S11. Variegated leaf phenotype of transplastomic pRB58 lines expressing the *Arabidopsis* QED1 protein
Close-up of the pRB58+UBQ::At-QED1 lines shown in Figure 9B. Overexpression of QED1 resulted in the appearance of pale and necrotic spots on leaves. Photograph was taken 16 weeks after sowing. Scale bar: 1 cm.

References

Cheng S, Gutmann B, Zhong X, Ye Y, Fisher MF, Bai F, Castleden I, Song Y, Song B, Huang J, et al. 2016. Redefining the structural motifs that determine RNA binding and RNA editing by pentatricopeptide repeat proteins in land plants. Plant J 85:532-547.

Hofacker IL. 2009. RNA secondary structure analysis using the Vienna RNA package. Curr Protoc Bioinformatics Chapter 12:Unit12 12.

Lurin C, Andres C, Aubourg S, Bellaoui M, Bitton F, Bruyere C, Caboche M, Debast C, Gualberto J, Hoffmann B, et al. 2004. Genome-wide analysis of Arabidopsis pentatricopeptide repeat proteins reveals their essential role in organelle biogenesis. Plant Cell 16:2089-2103.

Schmidt GW, Delaney SK. 2010. Stable internal reference genes for normalization of real-time RT-PCR in tobacco (Nicotiana tabacum) during development and abiotic stress. Mol Genet Genomics 283:233-241.
